# Benchmarking urinary cell transcriptomes for noninvasive differentiation of BK polyomavirus–associated nephropathy from T cell–mediated rejection

**DOI:** 10.1172/jci.insight.201060

**Published:** 2026-06-08

**Authors:** Franco B. Mueller, Carol Li, Darshana M. Dadhania, Surya V. Seshan, Thalia Salinas, Vijay K. Sharma, Jenny Z. Xiang, Hans H. Hirsch, Thangamani Muthukumar, Manikkam Suthanthiran

**Affiliations:** 1 Division of Nephrology and Hypertension, Department of Medicine, Weill Cornell Medicine, New York, New York, USA.; 2 Department of Transplantation Medicine, NewYork-Presbyterian Hospital, New York, New York, USA.; 3 Division of Renal Pathology, Department of Pathology and Laboratory Medicine, Weill Cornell Medicine, New York, New York, USA.; 4 Genomics Resources Core Facility, Department of Microbiology and Immunology, Weill Cornell Medical College, New York, New York, USA.; 5 Transplantation and Clinical Virology, Department of Biomedicine, University of Basel, Basel, Switzerland.

**Keywords:** Immunology, Nephrology, T cells, Transcriptomics, Transplantation

## Abstract

BK polyomavirus–associated nephropathy (BKVN) adversely impacts kidney allograft survival and often mimics acute T cell–mediated rejection (TCMR), confounding diagnosis and management. To address this conundrum, we performed unbiased RNA sequencing of urinary cells matched to biopsies classified as BKVN with intragraft inflammation (BKVN-P), BKVN without inflammation (BKVN-N), TCMR, or no rejection (NR). BKVN-N displayed dominant host DNA replication, cell cycle, and repair programs, while BKVN-P samples exhibited expansive innate immune activation, antigen presentation, chemokine upregulation, and epithelial injury. Both BKVN subtypes shared signatures of T cell exhaustion and mature and tolerogenic dendritic cell activation but differed in immune orientation — Th1 predominance in BKVN-N versus Treg and CD8 enrichment in BKVN-P. Compared with TCMR samples, BKVN-P lacked robust TCR/CD28 signaling and was enriched for viral and innate modules; BKVN-N lacked alloimmune activation. B cell exhaustion characterized BKVN-N, while BKVN-P displayed robust B cell activation with metabolic downregulation. A ratiometric urinary cell biomarker, CXCL10 mRNA/CD3E mRNA, distinguished both BKVN subtypes from TCMR with diagnostic accuracy, replicated by quantitative reverse transcription PCR for clinical translation, and confirmed in an independent cohort. These findings demonstrate the utility of urinary cell transcriptomics for resolving viral injury from alloimmunity, enabling precision diagnostics and targeted immunomodulation in kidney transplantation.

## Abstract 2

BK polyomavirus infection, rivaling rejection and complicating its diagnosis, undermines kidney transplantation. Urinary cell molecular profiling reveals mechanisms enabling targeted therapeutics and noninvasive differential diagnosis.

## Graphical abstract

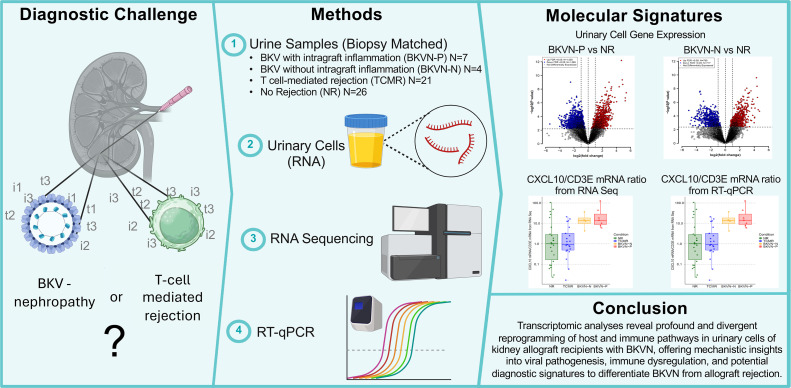

## Introduction

BK polyomavirus, a double-stranded DNA virus of the *Polyomaviridae* family, infects most of the human population during early childhood and establishes lifelong persistence in the genitourinary tract (1, 2). In immunocompetent individuals, BK polyomavirus remains clinically quiescent; however, in kidney allograft recipients, immunosuppressive therapy can precipitate viral reactivation, leading to BK polyomavirus–associated nephropathy (BKVN) (3).

BKVN is often a destructive tubulointerstitial disease characterized by viral cytopathic changes and intragraft inflammation that often mimics acute T cell–mediated rejection (TCMR), precipitating a critical diagnostic and clinical dilemma (4, 5). With current estimates of graft failure ranging from 30% to 50%, unequivocal diagnosis and effective management of BKVN are a pivotal challenge in kidney transplantation, which is exacerbated in patients at high risk of TCMR (6, 7).

Despite its clinical importance, the molecular mechanisms underlying BKVN replication and its impact on tissue injury remain largely unknown (8). Current diagnostic approaches — centered on plasma and urine viral load and invasive kidney allograft biopsy — are limited in their ability to resolve molecular events or provide mechanistic insights to guide rational therapy. No approved antiviral therapies exist for BK polyomavirus replication and disease, and the current strategy of reducing immunosuppression is associated with increased risk of donor-specific anti-HLA antibody formation and allograft rejection, likely contributors to graft failure following biopsy-confirmed BKVN (9).

RNA sequencing (RNA-seq) has revolutionized transcriptomic analysis by enabling unbiased characterization of gene expression with single-base resolution, minimal background noise, and broad dynamic range (10–12) and enabling detection of cellular heterogeneity, identification of transcriptional programs, and discovery of novel biomarkers (13–15).

We previously developed urinary cell mRNA profiling as a noninvasive method to interrogate human kidney allograft status (16–19). We performed urinary cell transcriptomics on urine samples matched to kidney allograft biopsies and identified distinct and overlapping signatures of TCMR and active antibody-mediated rejection (13). Our urinary cell mRNA profiling validated urine as an excellent surrogate for kidney allograft biopsy and demonstrated its utility in capturing biologically and clinically relevant intragraft molecular events (20).

In the present investigation, we applied RNA-seq to urine samples matched to BKVN biopsies representing a spectrum of intragraft inflammation, and prototypical TCMR biopsies and biopsies with no rejection (NR). Our in-depth transcriptomic analyses reveal profound and divergent reprogramming of host and immune pathways in urinary cells of kidney allograft recipients with BKVN, offering mechanistic insights into viral pathogenesis, immune dysregulation, and potential diagnostic signatures to differentiate BKVN from TCMR. These findings set the stage for new molecular approaches to noninvasive diagnosis, risk stratification, and tailored therapy in the care of kidney transplant recipients.

## Results

Additional details are provided in Supplemental Methods and Results (supplemental material available online with this article; https://doi.org/10.1172/jci.insight.201060DS1).

### Study design.

We investigated urinary cell gene expression in urine samples matched to kidney allograft biopsies, selected to represent 3 diagnostic categories: BKVN, TCMR, and no rejection (NR). Demographic and clinical details, biopsy findings, and total RNA characteristics are summarized in Supplemental Table 1, A–E.

**Figure 1:**
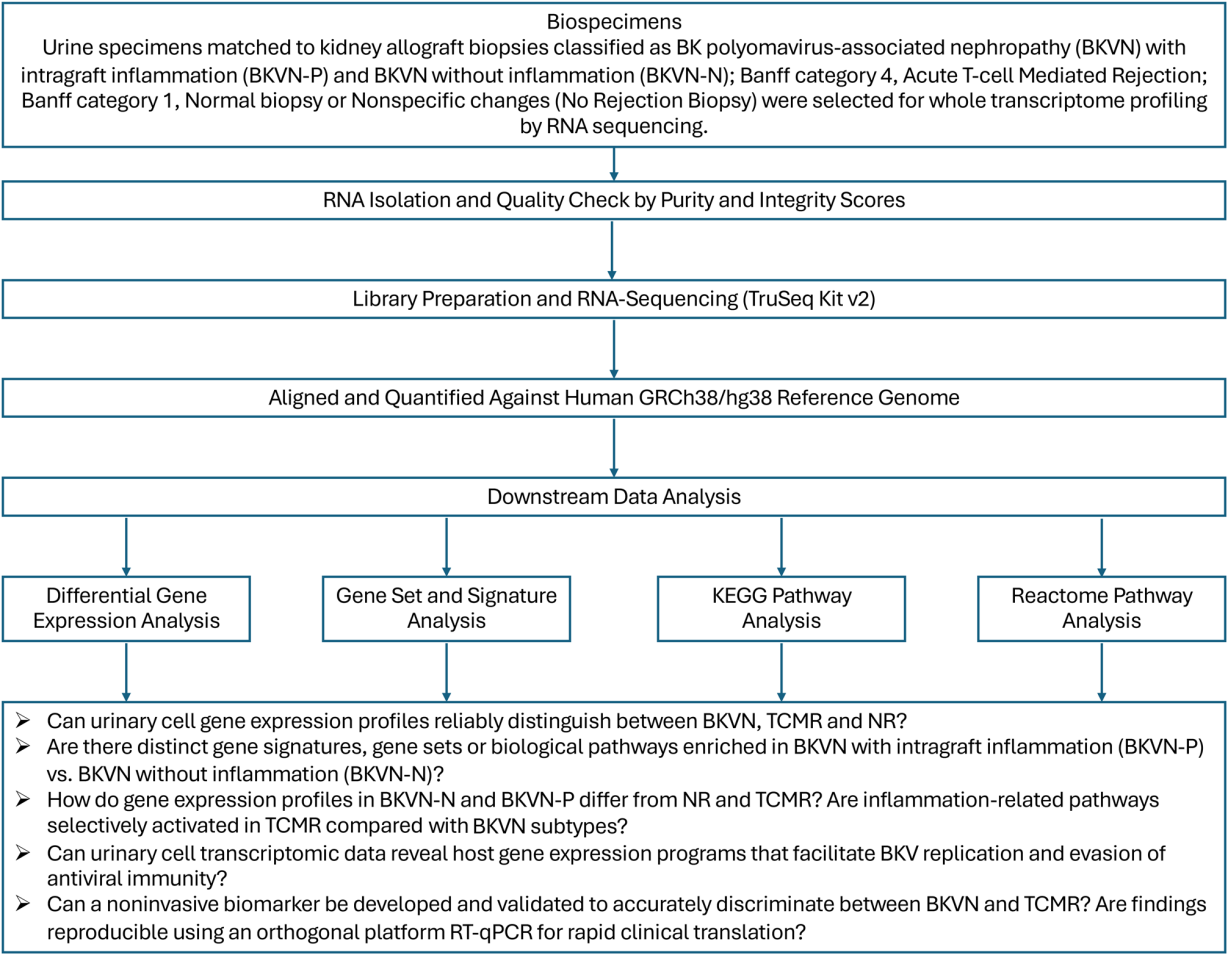
Workflow for RNA-seq of urine samples matched to kidney allograft biopsies. Urine samples matched to kidney allograft biopsies were selected from the Weill Cornell biorepository to include BK polyomavirus–associated nephropathy (BKVN) with intragraft inflammation (BKVN-P); BKVN without intragraft inflammation (BKVN-N); Banff category 4, acute T cell–mediated rejection (TCMR); or Banff category 1, normal biopsy or nonspecific changes (designated as no rejection). Urine was centrifuged, and total RNA was isolated from the sedimented urinary cell pellet. RNA quantity, purity, and integrity were assessed, and ribosomal RNA (rRNA) was depleted. cDNA libraries were prepared using the TruSeq Sample Preparation kit v2 (Illumina), followed by RNA-seq on an Illumina platform. Sequence reads were stored in FASTQ format and aligned to the human reference genome GRCh38/hg38 using the STAR aligner. Gene-level quantification was performed using CuffLinks (v2.2.1) and HTSeq to obtain raw counts and fragments per kilobase of transcript per million mapped reads (FPKM) values, which were subsequently converted to transcripts per million (TPM). Genes with counts per million greater than 1 in at least 2 samples were retained. Differential gene expression analysis was conducted using robust statistical methods at the individual gene level, gene set level, and pathway level (KEGG and Reactome) to address the biological questions listed in the bottom panel of the figure.

**Figure 2:**
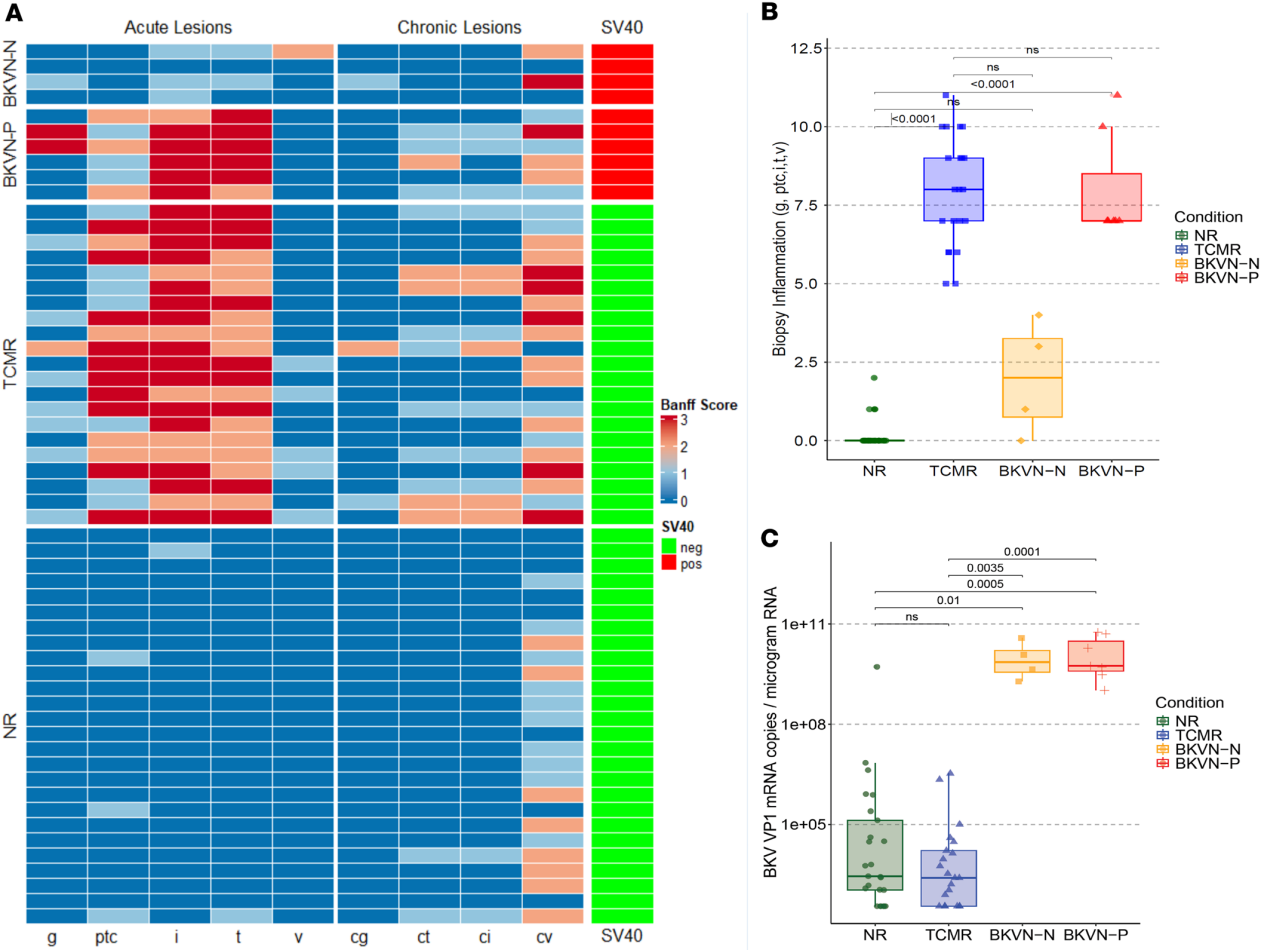
Banff lesion scores in kidney allograft biopsies and BKVN-VP1 mRNA copy numbers in biopsy-matched urines. (**A**) Heatmap showing Banff acute lesion scores — glomerulitis (g), peritubular capillaritis (ptc), interstitial infiltration (i), tubulitis (t), intimal arteritis (v) — and chronic lesion scores — chronic glomerulopathy based on glomerular basement double contours (cg), tubular atrophy (ct), interstitial fibrosis (ci), and vascular intimal thickening (cv) — in biopsies classified as BKVN-N (*n* = 4), BKVN-P (*n* = 6), TCMR (*n* = 21), or NR (*n* = 26). Lesion scores, 0 to 3, are color-coded as indicated. SV40/BK polyomavirus large T antigen immunostaining using PAb416 monoclonal antibody is shown. Red indicates positive staining; green, negative staining. (**B**) Box-and-whisker plots of summed acute Banff lesion scores (g + ptc + i + t + v) across diagnostic categories. Each point represents the summed score in an individual biopsy. Boxes indicate the interquartile range (IQR), with the horizontal line representing the median; whiskers denote the 10th and 90th percentiles. Statistical comparisons were performed using the Kruskal-Wallis test, followed by Dunn’s post hoc test. *P* values from Dunn’s test are shown at the top (Supplemental Table 1D). (**C**) Box-and-whisker plots of BK polyomavirus VP1 mRNA copy numbers in biopsy-matched urine samples, quantified by a customized RT-qPCR assay. Plot structure and statistical analysis are the same as in **B**. BK polyomavirus VP1 mRNA copies in urines matched to BKVN-N and BKVN-P biopsies exceeded the BKVN diagnostic threshold in every instance, while BK polyomavirus VP1 mRNA copies in urines matched to NR or TCMR biopsies did not exceed the diagnostic threshold for BKVN. *P* values from Dunn’s post hoc test are shown at the top (Supplemental Table 1E).

A total of 58 urine samples, matched to 57 kidney allograft biopsies from 53 patients, underwent ensemble RNA-seq. Figure 1 illustrates the study workflow designed to address critical unmet questions in clinical kidney transplantation. Figure 2A is a heatmap of Banff acute and chronic lesion scores of the 57 biopsies. Figure 2B, box-and-whisker plots of summed acute lesion scores, illustrates that the distribution of scores differed significantly among the diagnostic groups (Kruskal-Wallis test, *P* < 0.0001). Figure 2C, box-and-whisker plots of urinary cell BK polyomavirus VP1 mRNA copy numbers, shows that the copy number significantly differed across the diagnostic groups (*P* < 0.0001) and that BK polyomavirus VP1 mRNA copy number in urines matched to BKVN biopsies consistently exceeded the diagnostic threshold for BKVN (21, 22).

The BKVN biopsies were selected to represent a range of intragraft inflammation. Six exhibited considerable inflammation, while 4 had minimal or none. Based on Banff acute lesion scores, biopsies were grouped for analysis: BKVN-P (with inflammation), 7 urines matched to 6 biopsies with interstitial inflammation (i > 2) and tubulitis (t > 2) consistent with TCMR; BKVN-N (without inflammation), 4 urines matched to 4 biopsies with combined i + t scores less than 2.

### Differential gene expression analysis of urinary cell transcriptomes.

We compared urinary cell transcriptomes matched to kidney allograft biopsies using multigroup linear modeling with the limma R package (23), which applies Bayes moderation to improve variance estimates, particularly for low-expression genes.

### BKVN-P versus NR.

**Figure 3:**
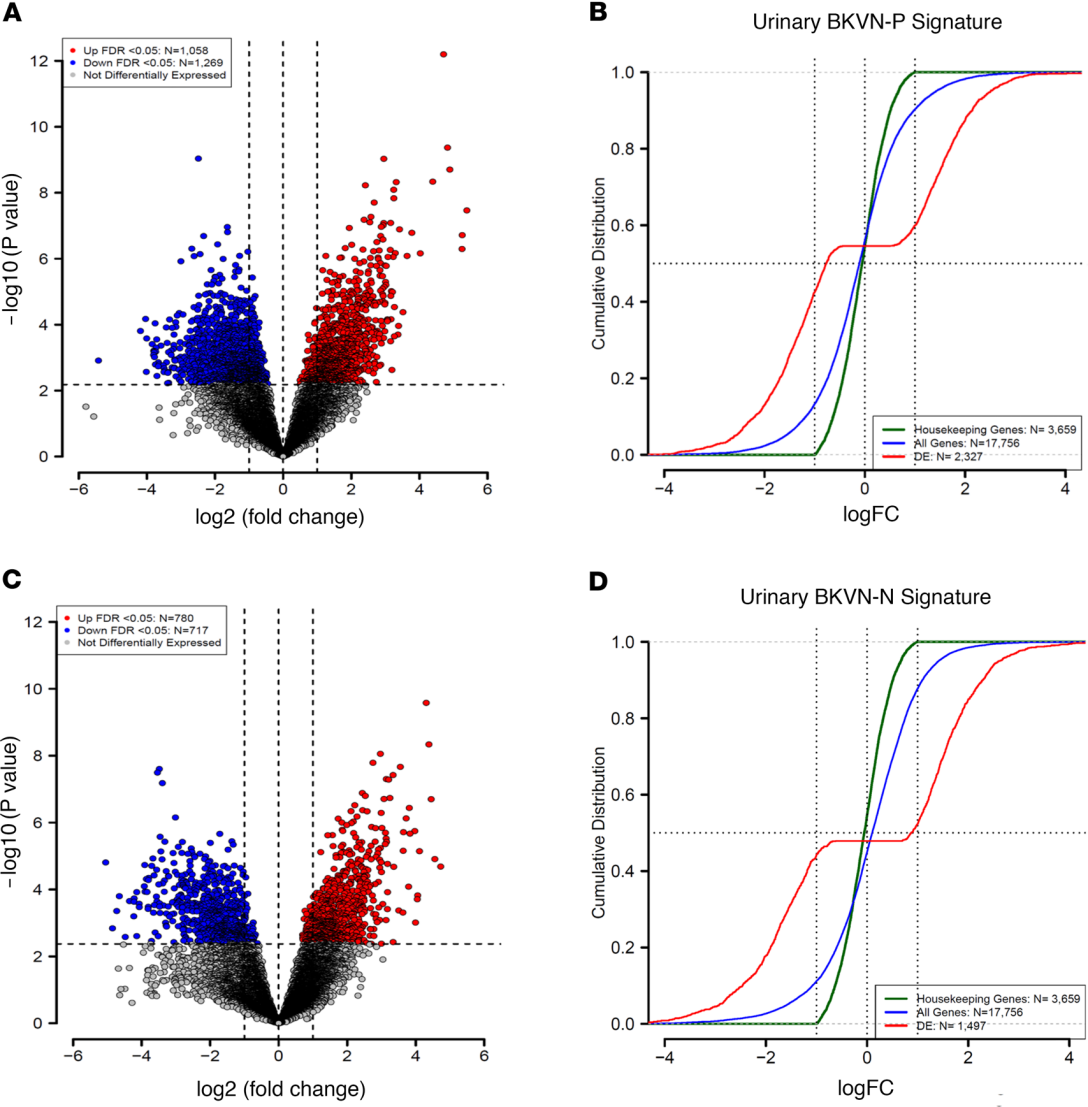
Differential gene expression analysis of urinary cell transcriptomes. (**A**) Volcano plot of urinary cell gene expression in 7 urines matched to 6 BKVN-P biopsies versus 26 urines matched to 26 NR biopsies. (**B**) CDF plot of gene expression ratios in 7 urines matched to 6 BKVN-P biopsies versus 26 urines matched to 26 NR biopsies. (**C**) Volcano plot of urinary cell gene expression in 4 urines matched to 4 BKVN-N biopsies versus 26 urines matched to 26 NR biopsies. (**D**) CDF plot of gene expression ratios in 4 urines matched to 4 BKVN biopsies versus 26 urines matched to 26 NR biopsies. A total of 17,756 genes were analyzed. In volcano plots, each red dot represents an overexpressed gene, and each blue dot represents an underexpressed gene, based on FDR-adjusted *P* value < 0.05. Gray/black dots indicate genes that are not differentially expressed. The *x* axis shows the log_2_ fold change (FC), calculated as the ratio of mRNA counts between the 2 groups being compared. The *y* axis represents the –log_10_ of the *P* values. In the CDF plots, the blue lines represent all 17,756 genes, the red lines represent DEGs, and the green lines represent 3,659 ubiquitously expressed housekeeping genes (24). The shift of the red curve relative to the respective green curve is statistically significant based on the Kolmogorov-Smirnov test. Supplemental Tables 2 and 3 list the BKVN-P versus NR DEGs shown in **A** and **B**. Supplemental Tables 4 and 5 list the BKVN-N versus NR DEGs shown in **C** and **D**.

Among the 17,756 protein-coding genes, 1,058 genes (5.95%) were significantly overexpressed (adjusted *P* < 0.05) and 1,269 genes (7.14%) were significantly underexpressed (adjusted *P* < 0.05) in the BKVN-P group (Figure 3A; differentially expressed genes (DEGs) listed in Supplemental Tables 2 and 3, respectively).

Cumulative distribution function (CDF) plots (Figure 3B) show a significant shift in DEGs (red curve) compared with housekeeping genes (green curve) (24), indicating a distinct transcription profile (Kolmogorov-Smirnov [K-S] test, *D* = 0.452, *P* < 2.2 × 10^–16^).

### BKVN-N versus NR.

780 genes (4.39%) were significantly overexpressed and 717 (4.03%) were significantly underexpressed in BKVN-N samples (Figure 3C; DEGs listed in Supplemental Tables 4 and 5, respectively).

CDF plots (Figure 3D) show a similar significant shift (K-S test, *D* = 0.494, *P* < 2.2 × 10^–16^), highlighting a unique transcriptional signature in BKVN-N.

### Host cell genes involved in replication.

Of the 22 SV40-related genes (25, 26), 14 were overexpressed in BKVN-N, 5 in BKVN-P, and 4 in TCMR, compared with NR samples (Supplemental Table 6A, panel A). These in vivo findings are consistent with prior in vitro data showing upregulation of these genes in kidney epithelial cells by SV40 (26, 27). Pairwise comparison of fold change revealed significantly higher expression in BKVN-N compared with BKVN-P (*P* = 0.0008) and TCMR (*P* < 0.0001) but not between BKVN-P and TCMR (Supplemental Table 6A, panel B).

### Host cell genes involved in cell cycle progression.

BK polyomavirus hijacks host cell cycle pathways to enhance its replication (28). Genes promoting cell cycle progression were upregulated in BKVN-N, BKVN-P, and TCMR, compared with NR (Supplemental Table 6B, panel A). The expression pattern aligned with the effects of viral large T antigen on pRB and TP53 (27, 29). Pairwise comparisons showed higher fold change in BKVN-N compared with BKVN-P (*P* < 0.0004) and with TCMR (*P* < 0.0001) (Supplemental Table 6B, panel B).

### Host cell genes involved in DNA replication.

Cell cycle gene activation during viral infection is tightly linked to dysregulation of host DNA replication (30). Compared with NR, multiple DNA replication genes were upregulated in BKVN-N and BKVN-P (Supplemental Table 6C, panel A). Fold change was higher in BKVN-N compared with BKVN-P (*P* = 0.0453) and with TCMR (*P* < 0.001) (Supplemental Table 6C, panel B).

### Host cell genes involved in repair pathways.

Both BKVN groups and TCMR showed increased expression of homologous-recombination repair genes, reflecting persistent DNA damage from viral replication and oxidative stress (31) (Supplemental Table 6D, panel A).

Fold change was higher in BKVN-N compared with BKVN-P (*P* = 0.0093) and with TCMR (*P* < 0.0001) (Supplemental Table 6D, panel B). Conversely, POLR1D, POLR2M, and CUL4B were downregulated in BKVN-N, suggesting transcriptional shutdown, viral manipulation of host transcriptional machinery, and tubular epithelial cell injury or dedifferentiation (Supplemental Table 6D, panel C). In BKVN-P, reduced ERCC2, POLR2M, and EME2 expression pointed to impaired DNA repair and transcriptional fidelity, and potential cell cycle arrest or apoptosis (Supplemental Table 6D, panel C). ERCC2 was the only gene underexpressed in TCMR. However, fold-change differences among BKVN-N, BKVN-P, and TCMR samples were not different (Supplemental Table 6D, panel D).

### Host cell genes involved in microtubule dynamics.

In BKVN-N, upregulated mitotic kinesins together with γ-tubulin complex genes (Supplemental Table 6E, panel A) indicated a proliferative epithelial program with reinforced centrosomes and mitotic spindles, while the downregulated genes (Supplemental Table 6E, panel B) suggested attenuation of RAB11-mediated apical recycling and long-range endosomal transport. This shift redirects microtubule usage away from polarity maintenance toward spindle assembly and perinuclear viral trafficking (32).

In BKVN-P, overexpression of DNAH1 indicated activation of a motile cilia/axonemal transcriptional module in injured proximal tubules (Supplemental Table 6E, panel A), whereas downregulation of multiple cytoplasmic dynein-dynactin adaptors and Rab11 axis genes (Supplemental Table 6E, panel B) suggested weakened cytoplasmic dynein cargo transport and impaired RAB11-dependent apical recycling. This pattern was consistent with dedifferentiated or regenerating tubular cells following severe injury and loss of epithelial polarity, and re-engagement of repair pathways.

In TCMR, the gene expression pattern (Supplemental Table 6, panels A and B) suggested highly reconfigurable microtubules optimized for vesicle transport, cell migration, and immune synapse formation, loss of ciliary programs, and weakened RAB11-driven epithelial polarity.

Supplemental Table 6E, panel C, shows that fold change is higher in BKVN-N compared with BKVN-P (*P* = 0.0017) and with TCMR (*P* < 0.0001). Supplemental Table 6E, panel D, shows that fold change is significantly higher in TCMR than in BKVN-P.

### Mitochondrial gene expression.

BK polyomavirus may exploit mitochondrial pathways to support its replication and immune evasion (33). Differential expression analysis showed significant overexpression in urine samples matched to BKVN-N biopsies compared with BKVN-P (Dunn’s *P* < 0.0001) and TCMR (*P* < 0.001) (Supplemental Table 6F, panel A), but not between BKVN-P and TCMR samples (Supplemental Table 6F, panel B). These findings support the presence of a BK polyomavirus–associated mitochondrial transcriptome stress pattern.

### Antigen presentation in BKVN.

HLA class II genes, key to CD4^+^ T cell activation (34), were upregulated in both BKVN-P and TCMR, indicating active antigen presentation (Supplemental Table 7). In BKVN-N, only HLA-DMB, which facilitates peptide loading by removing CLIP from the MHC class II groove (35), was upregulated compared with NR, indicating a more targeted antigen presentation response.

### Pattern recognition receptors in BKVN.

Pattern recognition receptors detect viral components and initiate innate immune responses (36, 37). Their expression patterns in BKVN-N, BKVN-P, and TCMR are detailed in Supplemental Table 8. DHX58, MB21D1, CD209, and NOD1 were overexpressed in BKVN-N. BKVN-P showed broader upregulation compared with BKVN-N or TCMR, indicating stronger immune activation.

### Chemokine and chemokine receptors.

Multiple chemokine and receptor genes were overexpressed in BKVN-P versus NR, indicating active immune cell recruitment (Supplemental Table 9A). Conversely, CCL17 (Treg recruiter) and CXCL14 (antiviral defense and tissue homeostasis) were downregulated in BKVN-P, suggesting impaired immune regulation and homeostasis. CXC3CL1 was markedly overexpressed in BKVN-N, highlighting a distinct inflammatory profile. These findings reflect heightened inflammation with reduced immune regulation and disrupted homeostasis.

### Cytokines and cytokine receptors.

Cytokine and receptor genes were more broadly elevated in BKVN-P than in BKVN-N (Supplemental Table 9B). The reduced LIFR and CTF1 in BKVN-P suggested compromised tissue repair and increased injury.

### IFNs and TNF/receptor superfamily.

Genes in these families were predominantly expressed in BKVN-P versus BKVN-N, reinforcing heightened inflammation in BKVN-P (Supplemental Table 9C). Concurrent downregulation of ACVR2B, BMPR2, and EGF in BKVN-P indicates a shift away from repair and regulation toward a proinflammatory, injury-prone state.

### T cell signaling in BKVN.

T cells play a central role in immune responses (38). Of 115 T cell signaling genes (Supplemental Table 10A), 51 were differentially expressed across BKVN-N, BKVN-P, or TCMR versus NR (Supplemental Table 10B). In BKVN-N, CTLA4 and IL-10 mRNA was upregulated, alongside downregulation of genes reflecting a suppressed or exhausted T cell phenotype.

BKVN-P also showed dysregulated T cell responses, with increased TNF, IFNG, NFATC1, PDCD1, and CTLA4, indicating activation and potential exhaustion (39, 40). Downregulation of phosphatases pointed to impaired T cell signaling.

TCMR samples showed upregulation of genes associated with T cell activation (Supplemental Table 10C). In contrast, BKVN-N showed increased CTLA4 alone, while BKVN-P showed elevated CD4, CTLA4, and PDCD1, consistent with initial activation followed by exhaustion, potentially facilitating viral persistence.

### Expression of T cell exhaustion and anergy genes in BKVN.

**Figure 4:**
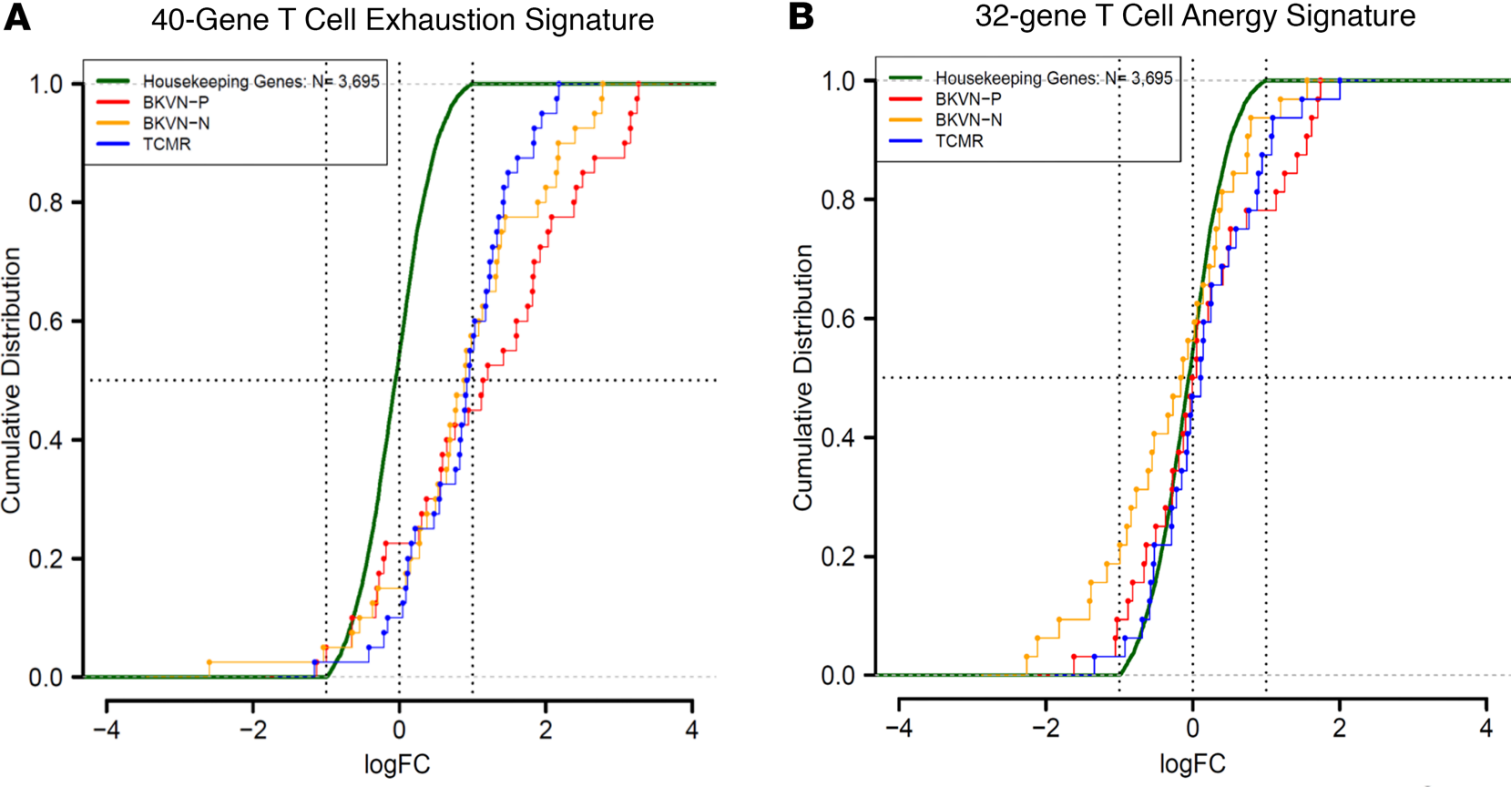
Cumulative distribution plots of the T cell exhaustion and T cell anergy signatures. (**A**) Gene list and gene set analyses for the 40-gene T cell exhaustion signature are provided in Supplemental Table 11, panels A and B, respectively. (**B**) Gene list and gene set analyses for the 32-gene list for the anergy gene signature are provided in Supplemental Table 12, panels A and B, respectively. The green curves represent the expression ratios (TCMR vs. NR) of 3,695 housekeeping genes. The blue, orange, and red curves correspond to expression ratios for TCMR/NR, BKVN-N/NR, and BKVN-P /NR, respectively. Significant rightward shifts of the gene expression curves, relative to housekeeping genes, were assessed using the Kolmogorov-Smirnov test, with *D* and *P* values reported in the Results section.

A 40-gene exhaustion signature (41–43) (Supplemental Table 11, panel A) was upregulated in BKVN-P (K-S test, *D =* 0.634, *P* = 3.15 × 10^–14^), BKVN-N (*D =* 0.640, *P* = 1.60 × 10^–14^), and TCMR (*D =* 0.658, *P* = 2.55 × 10^–15^) versus NR (Figure 4A), indicating robust enrichment.

In contrast, the 32-gene anergy signature (44) (Supplemental Table 12, panel A) showed modest downregulation in BKVN-N (K-S test, *D =* 0.264, *P* = 2.41 × 10^–02^), with non-significant shifts in BKVN-P (*D =* 0.224, *P* = 8.33 × 10^–2^) and TCMR (*D =* 0.229, *P* = 7.25 × 10^–2^) (Figure 4B).

ROAST (rotation gene set test) (45) and CAMERA (correlation-adjusted mean rank gene set test) (46) confirmed strong enrichment of exhaustion genes in BKVN subtypes and TCMR (Supplemental Table 11, panel B), while anergy genes showed limited enrichment in BKVN-N and none in BKVN-P (Supplemental Table 12, panel B). These findings support a model of chronic antigen-driven T cell exhaustion, likely contributing to immune evasion in BKVN.

### CD4^+^ T cells in BKVN.

Given CD4 overexpression in BKVN-P, we examined cytotoxic CD4^+^ T cell–associated genes. Several genes related to CD4^+^ cytotoxic T lymphocyte activity were upregulated in BKVN-P (Supplemental Table 13).

**Figure 5:**
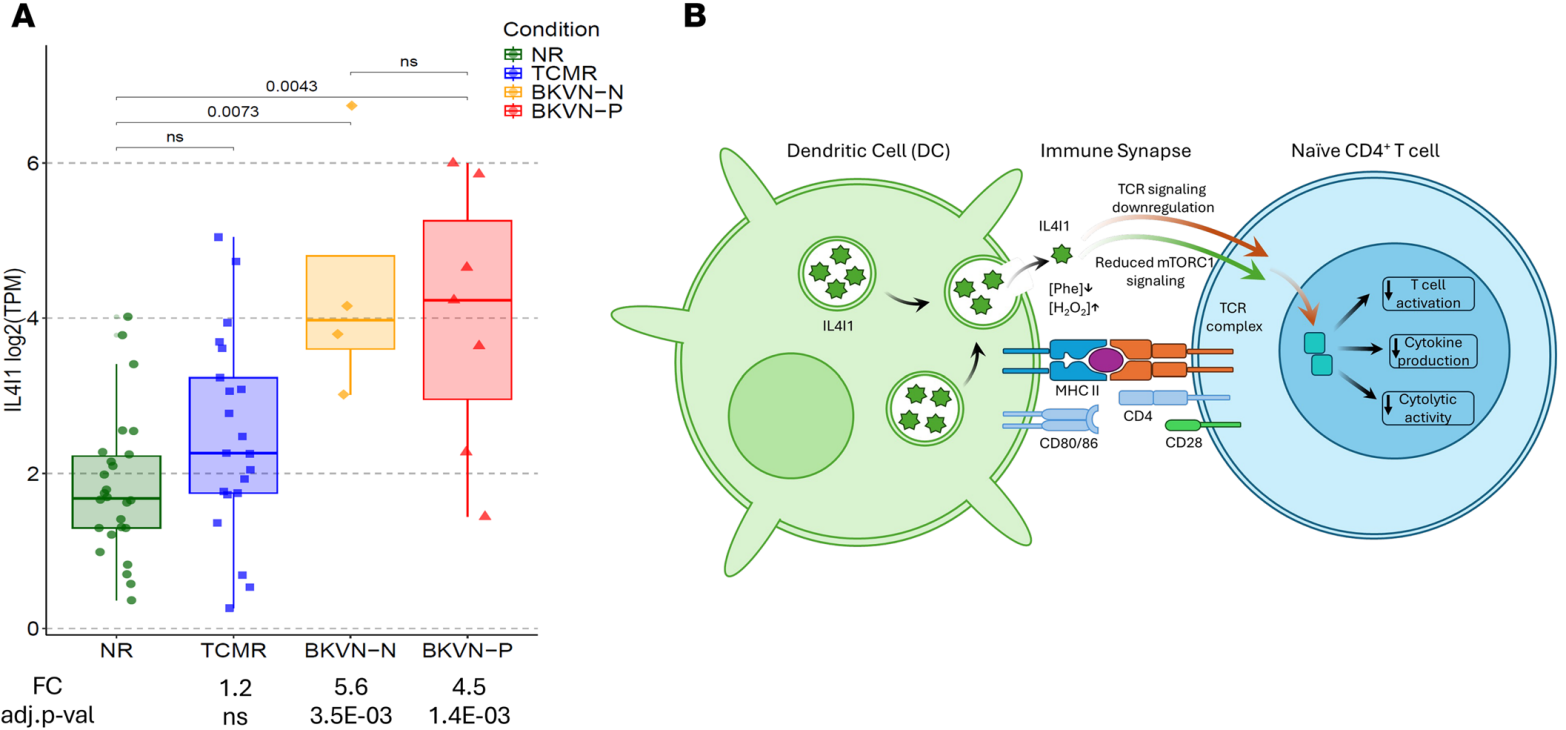
IL4I1 mRNA abundance in urinary cells. (**A**) Box-and-whisker plots display IL-4–induced gene 1 (IL4I1) mRNA abundance (log_2_ TPM) in urine samples matched to kidney allograft biopsies: 26 NR, 21 TCMR, 4 BKVN-N, and 7 urines matched to 6 BKVN-P biopsies. Each point represents IL4I1 mRNA abundance in individual samples. Boxes denote the 25th–75th percentiles, the horizontal line marks the median, and the whiskers represent the 10th–90th percentiles. Fold change values and adjusted *P* values are shown. Multigroup comparison used Kruskal-Wallis test followed by pairwise Dunn’s test, with Dunn’s test *P* values displayed at the top. (**B**) Schema for IL4I1 immunosuppressive activity. IL4I1, secreted at the immune synapse between activated dendritic cells and T cells, downregulates TCR signaling and T cell effector function by cleaving aromatic amino acids into keto acids and generating H_2_O_2_ and NH_3_. Through binding to TMPRSS13, IL4I1 also exerts non-enzymatic effects, including promoting Treg differentiation and reducing antibody production via T follicular helper cell–germinal B cell interactions (76).

Notably, IL-4–induced 1 (IL4I1), a myeloid-derived immunosuppressive enzyme, was significantly overexpressed in BKVN-N (*P* = 3.45 × 10^–3^) and BKVN-P samples (*P* = 1.43 × 10^–3^) versus NR (Figure 5A). IL4I1 may impair cytotoxic CD4^+^ T cell function via reduced TCR and mTORC1 signaling, potentially aiding viral immune evasion (Figure 5B).

### CD4^+^ T cell subset signatures in BKVN.

Prior RNA-seq analyses revealed enrichment of Th1, Th2, Th17, and Treg gene signatures in TCMR biopsies versus NR biopsies (38, 47, 48). We investigated these subsets in urines matched to BKVN biopsies.

### Th1 signature.

**Figure 6:**
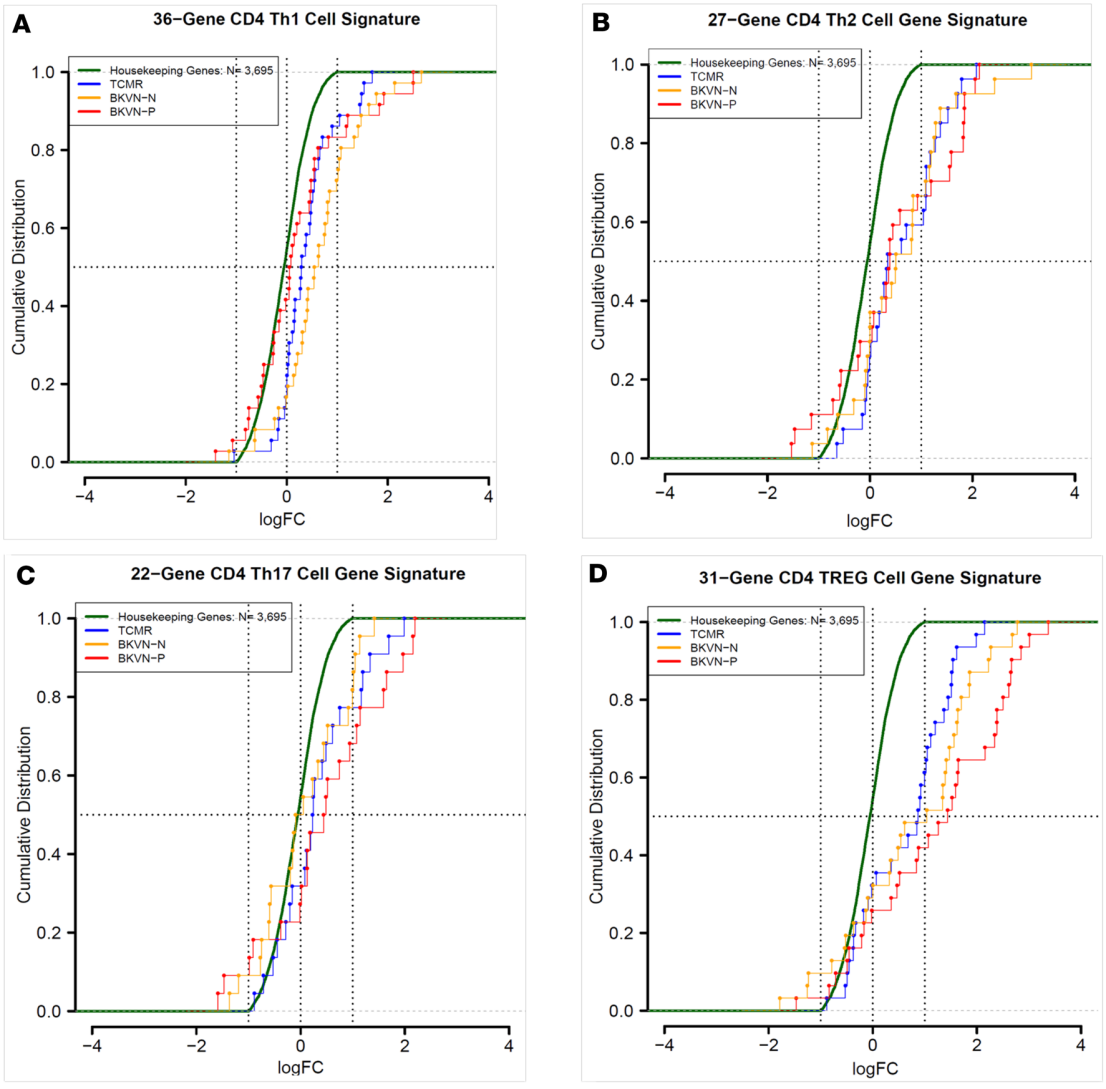
CDF plots of CD4^+^ Th1, Th2, Th17, and Treg cell signatures. Gene lists and gene set analyses for the 36-gene CD4^+^ Th1 signature (**A**), 27-gene CD4^+^ Th2-gene signature (**B**), 22-gene CD4^+^ Th17-gene signature (**C**), and 31-gene Treg signature (**D**) are provided in Supplemental Tables 14, 15, 16, and 17, respectively. The green curves represent the expression ratios (TCMR vs. NR) of 3,695 housekeeping genes. The blue, orange, and red curves correspond to expression ratios for TCMR/NR, BKVN-N/NR, and BKVN-P/NR, respectively. Significant rightward shifts of the gene expression curves, relative to housekeeping genes, were assessed using the Kolmogorov-Smirnov test, with *D* and *P* values reported in the Results section.

The Th1 signature (36 genes; Supplemental Table 14, panel A; ref. 48) was significantly upregulated in BKVN-N (K-S test, *D =* 0.670, *P* = 2.59 × 10^–14^), BKVN-P (*D =* 0.384, *P* = 5.31 × 10^–5^), and TCMR (*D =* 0.603, *P* = 1.08 × 10^–11^) versus NR (Figure 6A). BKVN-N showed stronger enrichment than BKVN-P (*D =* 0.361, *P* = 0.0176). ROAST and CAMERA confirmed robust enrichment in BKVN-N and TCMR (Supplemental Table 14, panel B).

### Th2 signature.

The Th2 signature (27 genes; Supplemental Table 15, panel A; ref. 48) was upregulated in BKVN-N (K-S test, *D =* 0.558, *P* = 1.14 × 10^–7^), BKVN-P (*D =* 0.568, *P* = 6.001 × 10^–8^), and TCMR (*D =* 0.568, *P* = 6.32 × 10^–8^) versus NR (Figure 6B). ROAST and CAMERA showed robust enrichment in TCMR and modest enrichment in BKVN subtypes (Supplemental Table 15, panel B).

### Th17 signature.

The Th17 signature (22 genes; Supplemental Table 16, panel A; ref. 48) was significantly enriched in BKVN-P (K-S test, *D =* 0.541, *P* = 5.65 × 10^–6^), BKVN-N (*D* = 0.361, *P* = 6.66 × 10^–3^), and TCMR (*D* = 0.508, *P* = 2.54 × 10^–5^) versus NR (Figure 6C). ROAST and CAMERA confirmed the statistically enriched Th17 signature in BKVN-P (and TCMR) compared with enrichment in BKVN-P and TCMR; BKVN-N showed non-significant enrichment (Supplemental Table 16, panel B).

### Treg signature.

The Treg signature (31 genes; Supplemental Table 17, panel A; ref. 47) was upregulated in BKVN-P (K-S test, *D* = 0.690, *P* = 3.75 × 10^–13^), BKVN-N (*D* = 0.619, *P* = 1.15 × 10^–10^), and TCMR (*D* = 0.594, *P* = 7.49 × 10^–10^) versus NR (Figure 6D). BKVN-P showed stronger enrichment than TCMR (*D* = 0.355, *P* = 3.96 × 10^–2^), but BKVN-N did not.

ROAST and CAMERA confirmed strong enrichment of Tregs in BKVN-P, and modest enrichment in BKVN-N (Supplemental Table 17, panel B).

Our investigation of CD4^+^ T cell subsets identified Th1 signature predominance in BKVN-N, and Treg signature being most enriched in BKVN-P. Altogether, our data suggest distinct CD4^+^ T cell subset dynamics across BKVN subtypes.

### CD8^+^ memory and effector T cell signatures in BKVN.

We assessed enrichment of CD8^+^ memory (45 genes; Supplemental Table 18, panel A; ref. 49) and effector (24 genes; Supplemental Table 19, panel A) T cell signatures in urines matched to BKVN or TCMR biopsies versus urines matched to NR biopsies.

### CD8^+^ memory T cell gene signature.

**Figure 7:**
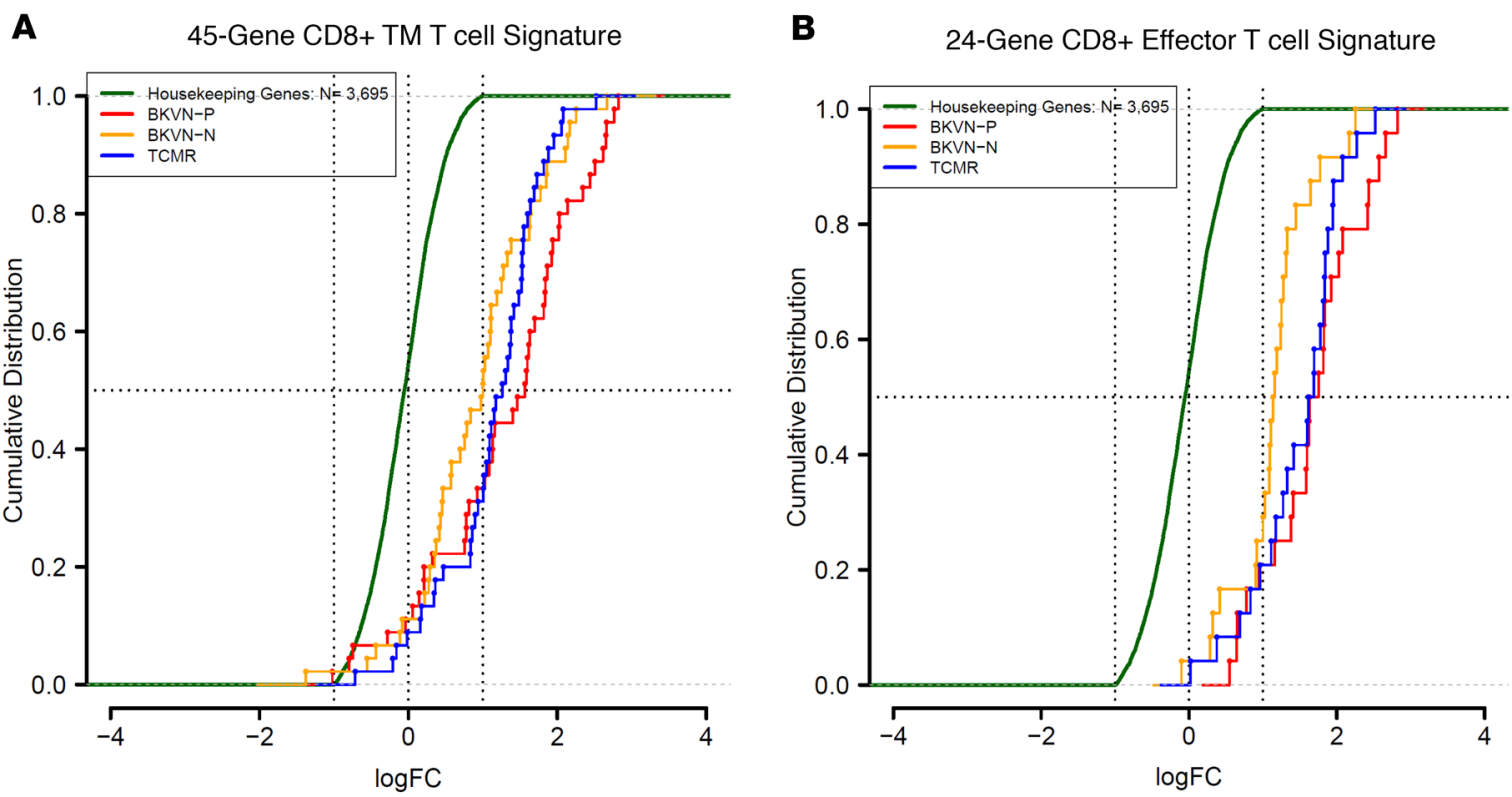
CDF plots of CD8^+^ memory and effector T cell gene signatures. (**A**) Gene list and gene set analyses of the 45-gene CD8^+^ memory T cell gene signature are provided in Supplemental Table 18, panels A and B, respectively. (**B**) Gene list and gene set analyses of the 24-gene CD8^+^ T cell gene signature are provided in Supplemental Table 19, panels A and B, respectively. The green curves represent the expression ratios (TCMR vs. NR) of 3,695 housekeeping genes. The blue, orange, and red curves correspond to expression ratios for TCMR/NR, BKVN-N/NR, and BKVN-P /NR, respectively. Significant rightward shifts of the gene expression curves, relative to housekeeping genes, were assessed using the Kolmogorov-Smirnov test, with *D* and *P* values reported in the Results section.

The CD8^+^ memory T cell gene signature was significantly upregulated in BKVN-P (K-S test, *D* = 0.756, *P* = 2.20 × 10^–16^), BKVN-N (*D* = 0.647, *P* = 2.20 × 10^–16^), and TCMR (*D* = 0.788, *P* = 2.20 × 10^–16^) versus NR (Figure 7A). Differences between BKVN-P and BKVN-N (*D* = 0.311, *P* = 0.025) and between BKVN-P and TCMR (*D* = 0.289, *P* = 0.046) were significant. ROAST and CAMERA confirmed the strong enrichment across all groups (Supplemental Table 18, panel B).

### CD8^+^ effector T cell gene signature.

The CD8^+^ effector T cell gene signature was upregulated in BKVN-P (K-S test, *D* = 0.934, *P* = 2.20 × 10^–16^), BKVN-N (*D* = 0.827, *P* = 1.39 × 10^–14^), and TCMR (*D* = 0.881, *P* = 2.20 × 10^–16^) versus NR (Figure 7B). Significant differences were observed between BKVN-P and BKVN-N (*D* = 0.5417, *P* = 0.001) and between BKVN-N and TCMR (*D* = 0.456, *P* = 0.011); the difference between BKVN-P and TCMR was not significant (*D* = 0.167, *P* = 0.902). ROAST and CAMERA confirmed strong enrichment in BKVN-P and TCMR samples (Supplemental Table 19, panel B).

### B cell signaling in BKVN.

Beyond antibody production, B cells may contribute to BKVN via antigen presentation and inflammation (50). Of 79 B cell signaling genes (Supplemental Table 20A), 31 were differentially expressed across BKVN-N, BKVN-P, or TCMR versus NR (Supplemental Table 20B).

### BKVN-N.

Upregulation of inhibitory receptors (LILRB1/4/5, CD72) and downregulation of KRAS, BCL10, and MAPK3 suggest a regulatory B cell phenotype with suppressed B cell signaling.

### BKVN-P.

Gene expression indicated active cell signaling, with upregulation of BTK, BLK, CD79A/B, NFATC1, JUN, NFKB1, RASGRP3, IFITM1, and LILRB1/4 alongside AKT2 downregulation, reflecting activation with regulatory feedback.

### TCMR.

Enhanced B cell activation and signaling were evident via increased LILRB1, IFITM, CD72, RAC2, PRKCB, PTPN6 (SHP-1), CARD11, FCGR2B, INPP5D (SHIP1), NFATC1/3, PIK3CD, and PPP3CC, highlighting a role for B cells in TCMR pathogenesis.

### Mature dendritic cell gene signature in BKVN.

**Figure 8:**
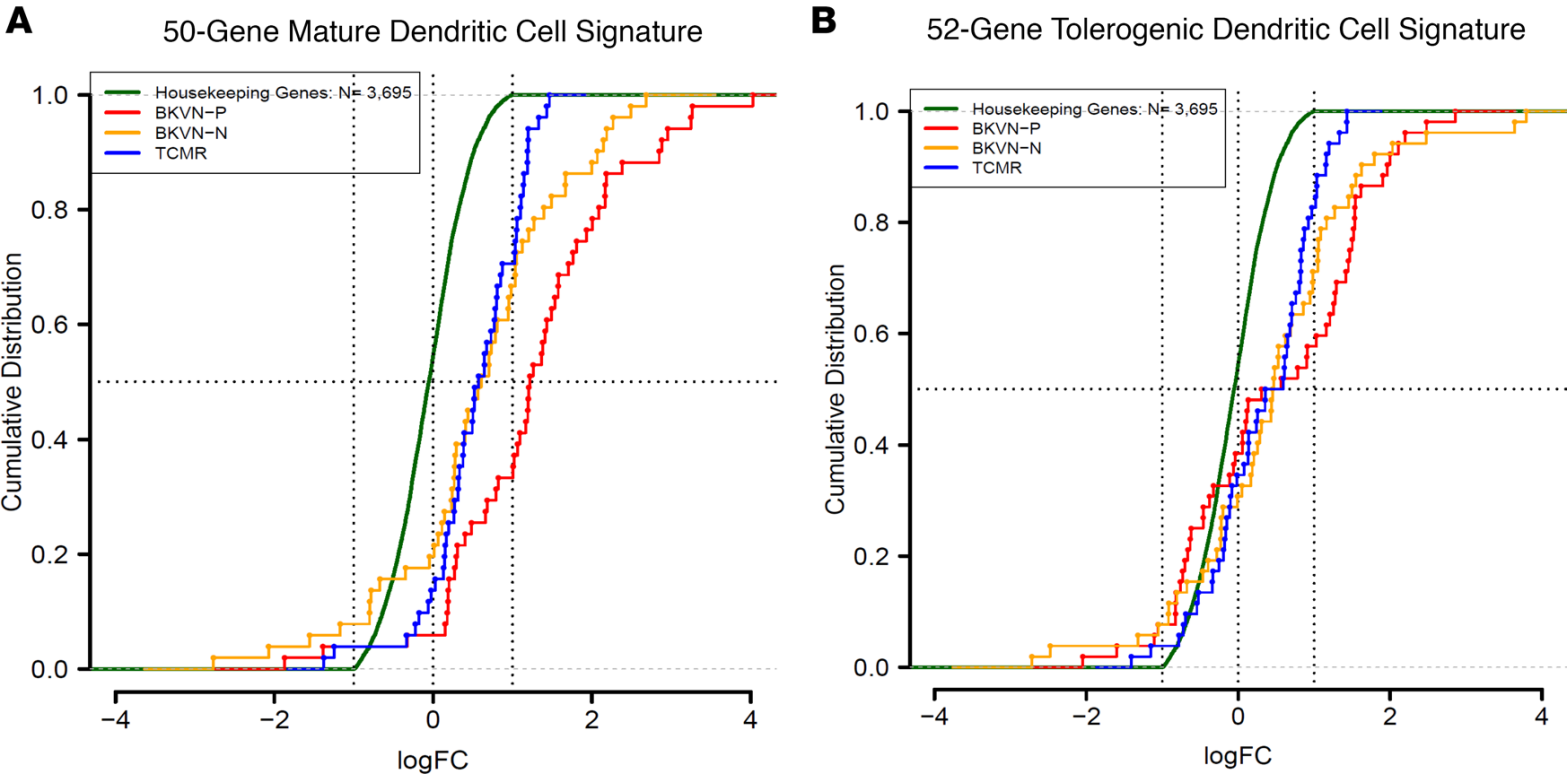
CDF plots of mature or tolerogenic dendritic cell gene signatures. (**A**) Gene list and gene set analyses of the 50-gene mature dendritic cell gene signature are provided in Supplemental Table 21, panels A and B, respectively. (**B**) Gene list and gene set analyses of the 52-gene tolerogenic dendritic cell gene signature are provided in Supplemental Table 22, panels A and B, respectively. The green curves represent the expression ratios (TCMR vs. NR) of 3,695 housekeeping genes. The blue, orange, and red curves correspond to expression ratios for TCMR/NR, BKVN-N/NR, and BKVN-P/NR, respectively. Significant rightward shifts of the gene expression curves, relative to housekeeping genes, were assessed using the Kolmogorov-Smirnov test, with *D* and *P* values reported in the Results section.

Dendritic cells (DCs) are central to shaping the immune landscape, acting as key initiators of antiviral defense (51). A mature DC signature (50 genes; Supplemental Table 21, panel A) was upregulated in BKVN-P (K-S test, *D* = 0.705, *P* = 2.20 × 10^–16^), BKVN-N (*D* = 0.499, *P* = 2.71 × 10^–11^), and TCMR (*D* = 0.541, *P* = 3.19 × 10^–13^) versus NR (Figure 8A). Pairwise comparisons revealed significant differences between BKVN-P and TCMR (*D* = 0.490, *P* = 6.33 × 10^–6^) and between BKVN-P and BKVN-N (*D* = 0.352, *P* = 0.003), while the difference between BKVN-N and TCMR was not significant (*D* = 0.196, *P* = 0.283). ROAST and CAMERA confirmed enrichment in BKVN-P and TCMR; BKVN-N versus NR was significant by CAMERA only (Supplemental Table 21, panel B).

### Tolerogenic DC gene signature in BKVN.

A tolerogenic DC gene signature (52 genes; Supplemental Table 22, panel A; ref. 52) was upregulated in BKVN-P (K-S test, *D* = 0.463, *P* = 5.83 × 10^–10^), BKVN-N (*D* = 0.448, *P* = 2.26 × 10^–09^), and TCMR (*D* = 0.446, *P* = 2.69 × 10^–9^) versus NR (Figure 8B). Only BKVN-P versus TCMR showed significant difference (*D* = 0.327, *P* = 0.007). ROAST and CAMERA confirmed enrichment across all groups versus NR (Supplemental Table 22, panel B).

In sum, mature DCs are strongly enriched in BKVN-P and TCMR, while tolerogenic DCs are upregulated in BKVN subtypes and TCMR, suggesting dual roles in antiviral defense and immune regulation (51, 52).

### Gene set enrichment analysis of KEGG and Reactome pathways in BKVN-P and BKVN-N relative to NR transcriptomes.

**Figure 9:**
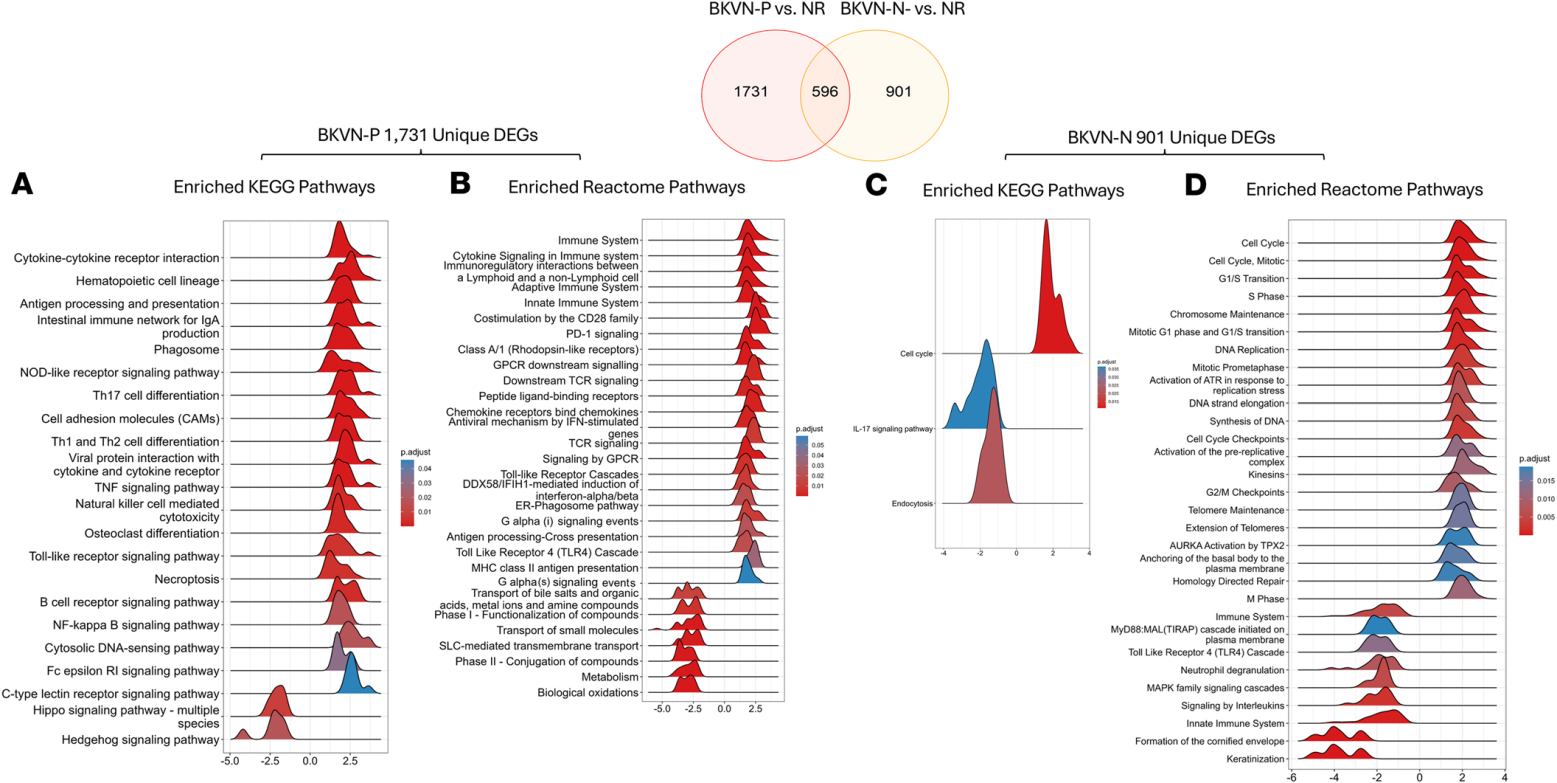
GSEA of KEGG and Reactome pathways uniquely enriched in BKVN-P and BKVN-N urines relative to NR urines. (**A** and **B**) Ridge plots showing enriched pathways based on the 1,731 DEGs unique to BKVN-P. (**A**) The 22 significantly enriched (adjusted *P* < 0.05) KEGG pathways (Supplemental Table 23). (**B**) The top 30 of 53 significantly enriched (adjusted *P* < 0.05) Reactome pathways (Supplemental Table 24). (**C** and **D**) Ridge plots of enriched pathways based on the 901 DEGs unique to BKVN-N. (**C**) The 3 significantly enriched (adjusted *P* < 0.05) KEGG pathways (Supplemental Table 25). (**D**) The top 30 of 62 significantly enriched (adjusted *P* < 0.05) Reactome pathways (Supplemental Table 26).

To provide functional context and mechanistic insights into the DEGs, we performed gene set enrichment analysis (GSEA) using both Kyoto Encyclopedia of Genes and Genomes (KEGG) and Reactome pathway databases. One key objective was to identify pathways uniquely enriched in the BKVN-P and BKVN-N groups. Among the 2,327 DEGs identified between BKVN-P and NR, 1,731 genes were unique to BKVN-P, and 596 overlapped with DEGs in BKVN-N versus NR. Conversely, among the 1,497 DEGs between BKVN-N and NR, 901 genes were unique to BKVN-N (Figure 9, Venn diagram).

Figure 9 presents ridge plot distributions of uniquely enriched pathways based on the DEGs unique to BKVN-P samples. Figure 9A displays 22 enriched KEGG pathways (adjusted *P* < 0.05; Supplemental Table 23), while Figure 9B shows the top 30 of 53 enriched Reactome pathways (adjusted *P* < 0.05; Supplemental Table 24). The positively enriched biological pathways reflected robust activation of immune responses and cytokine signaling, including IFN-mediated antiviral defense, B cell receptor signaling pathway, antigen presentation and costimulation, and proinflammatory chemokine activity. In contrast, the negatively enriched pathways suggested a loss of metabolic and solute transport functions of epithelial cells, impaired epithelial cell repair mechanisms, and compromised epithelial barrier integrity — likely consequences of viral cytopathic effects.

Figure 9 also displays ridge plot distributions of uniquely enriched pathways in BKVN-N. Figure 9C shows one positively enriched cell cycle pathway and two negatively enriched KEGG pathways, endocytosis and IL-17 signaling (adjusted *P* < 0.05; Supplemental Table 25), and Figure 9D presents the top 30 of 62 enriched Reactome pathways (adjusted *P* < 0.05; Supplemental Table 26). DNA damage response and repair, centrosome and microtubule organization, telomere maintenance, AURKA activation, and Golgi-to-ER retrograde transport features are consistent with BK polyomavirus–induced reprogramming of renal tubular epithelial cells. The negatively enriched pathways reflected suppression of host defense and epithelial cell differentiation, including downregulation of innate immune signaling, attenuated inflammatory responses, tubular injury, and loss of epithelial integrity.

**Figure 10:**
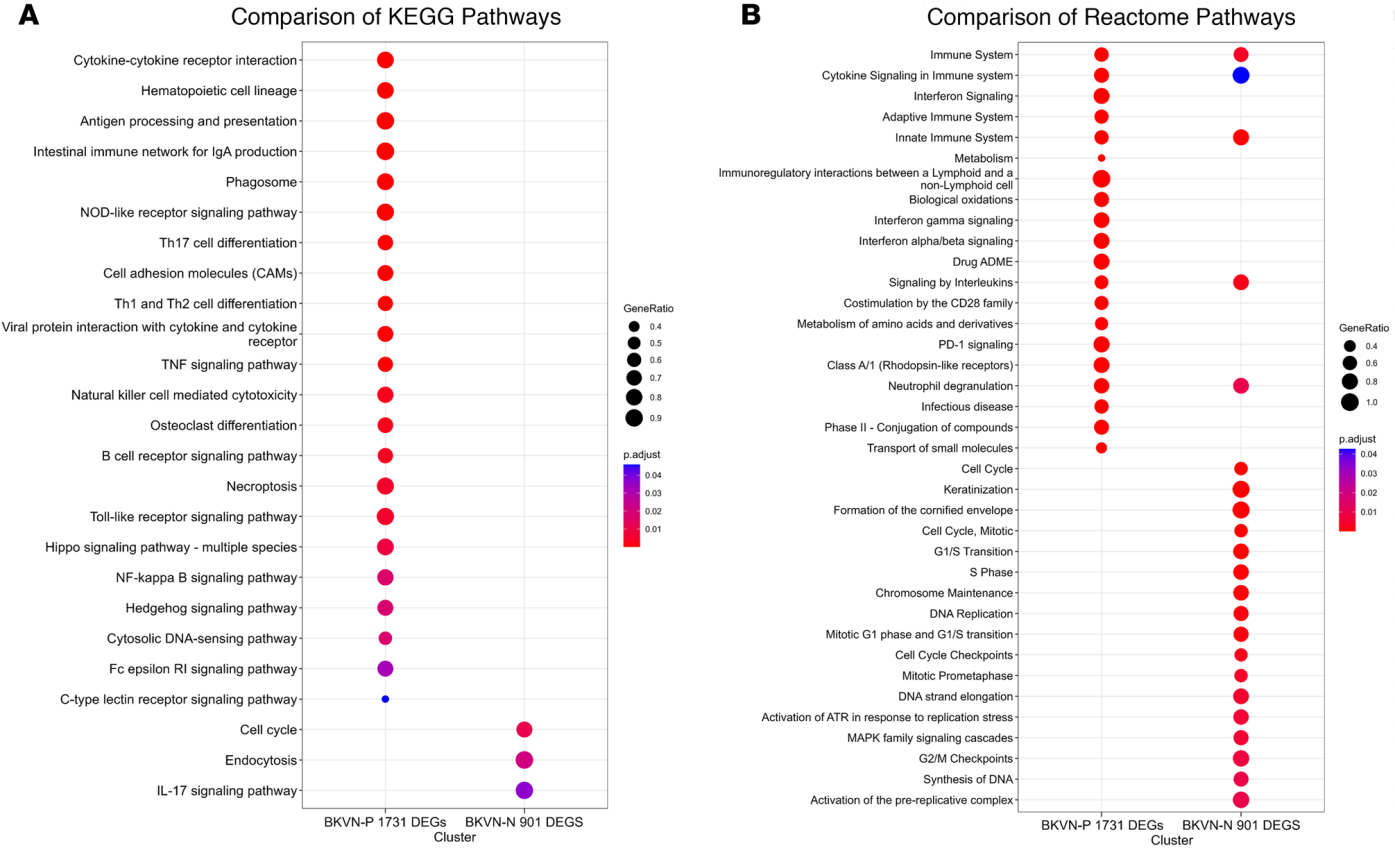
clusterProfiler comparison of KEGG and Reactome pathways enriched in BKVN-P and BKVN-N by GSEA. (**A**) clusterProfiler comparisons of enriched KEGG pathways by GSEA. (**B**) clusterProfiler comparisons of enriched Reactome pathways by GSEA.

The contrasts in pathway enrichment between BKVN-P and BKVN-N samples are illustrated through cluster profiling. Figure 10A compares GSEA results for KEGG pathways, while Figure 10B presents a similar comparison for Reactome pathways. These visualizations highlight distinct biological programs activated in each subgroup, underscoring the divergent molecular mechanisms underlying BKVN-P and BKVN-N subtypes.

### GSEA of KEGG and Reactome pathways uniquely enriched in BKVN-P and BKVN-N samples relative to TCMR transcriptomes.

**Figure 11:**
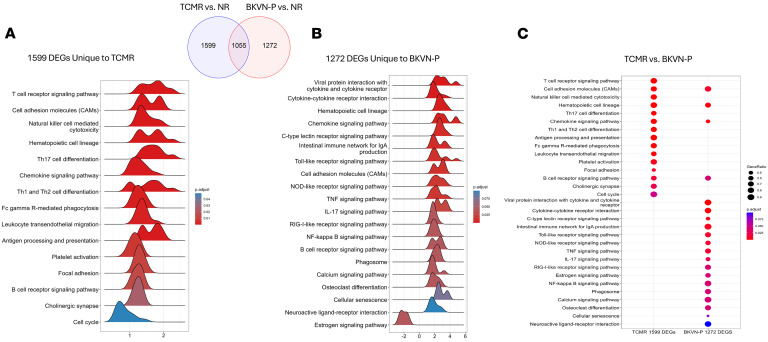
GSEA of KEGG pathways uniquely enriched in BKVN-P urines relative to TCMR urines. (**A**) Ridge plot showing enriched KEGG pathways (adjusted *P* < 0.05) based on the 1,599 DEGs unique to TCMR (Supplemental Table 27). (**B**) Ridge plot showing enriched KEGG pathways (adjusted *P* < 0.05) based on 1,272 DEGs unique to BKVN-P (Supplemental Table 28). (**C**) clusterProfiler comparison of KEGG pathways enriched in TCMR and BKVN-P by GSEA.

Given that the histologic features of BKVN-P are largely indistinguishable from those of TCMR, we investigated whether GSEA of KEGG and Reactome pathways could provide insights discriminating BKVN-P from TCMR. To this end, we first identified 2,654 DEGs by comparing urinary cell transcriptomes of urines matched to TCMR biopsies with those matched to NR biopsies. We then compared these DEGs with the 2,327 DEGs identified by comparison of urinary cell transcriptomes of BKVN-P versus NR. A comparison of 2,654 DEGs from TCMR versus NR with the 2,327 DEGs from BKVN-P versus NR revealed 1,599 DEGs unique to TCMR, 1,272 DEGs unique to BKVN-P, and 1,055 shared DEGs (Figure 11, Venn diagram).

**Figure 12:**
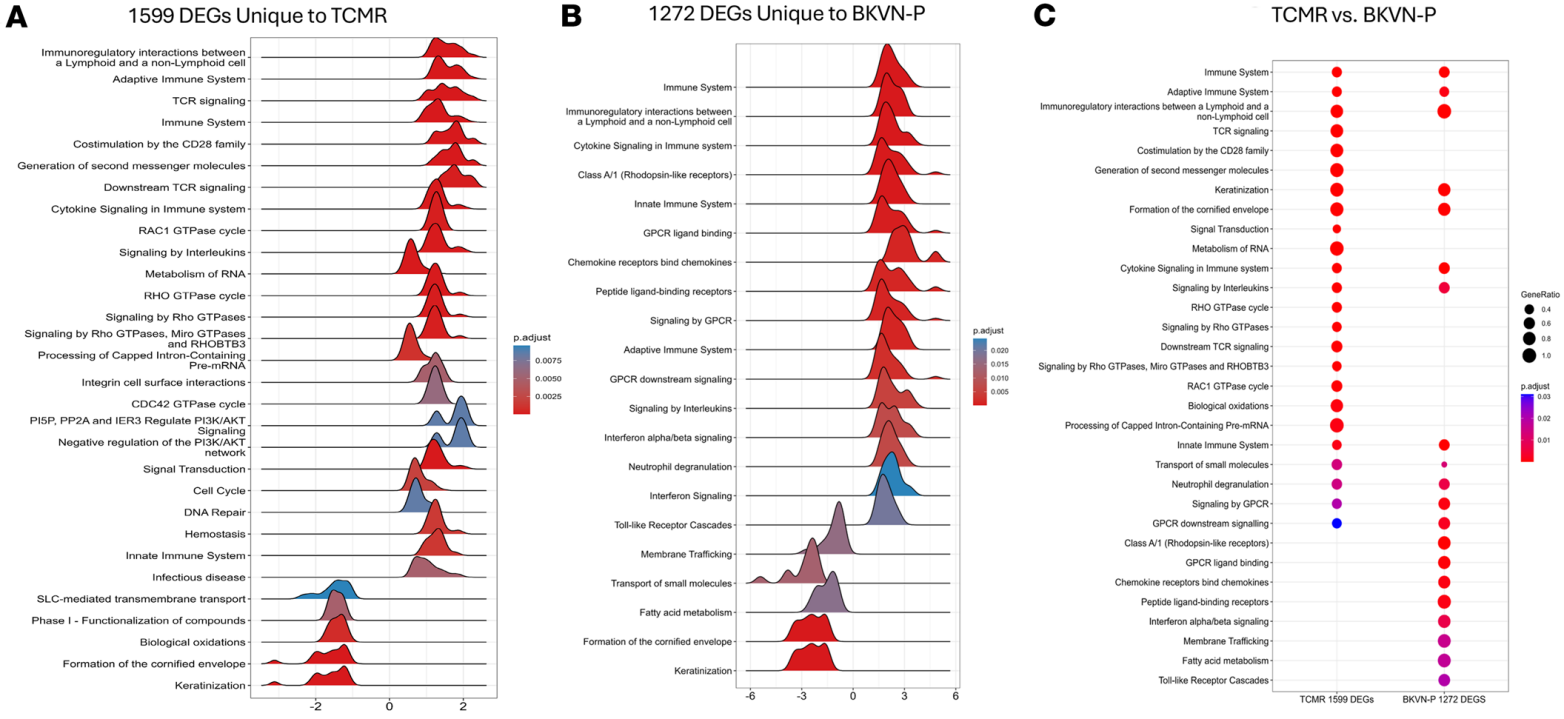
GSEA of Reactome pathways uniquely enriched in BKVN-P samples compared with TCMR transcriptomes. (**A**) Ridge plot of enriched Reactome pathways (adjusted *P* < 0.05) based on the 1,599 DEGs unique to TCMR (Supplemental Table 29). (**B**) Ridge plot of enriched Reactome pathways (adjusted *P* < 0.05) based on 1,272 DEGs unique to BKVN-P (Supplemental Table 30). (**C**) clusterProfiler comparison of Reactome pathways enriched in TCMR and BKVN-P by GSEA.

Figure 11A presents ridge plot distributions of enriched KEGG pathways based on the 1,599 DEGs unique to TCMR samples (Supplemental Table 27). Figure 11B displays enriched KEGG pathways based on the 1,272 DEGs unique to BKVN-P samples (Supplemental Table 28). Figure 11C compares enriched KEGG pathways of TCMR with those of BKVN-P. Figure 12A presents enriched Reactome pathways based on the 1,599 DEGs unique to TCMR (Supplemental Table 29). Figure 12B presents enriched Reactome pathways based on the 1,272 DEGs unique to BKVN-P (Supplemental Table 30). Figure 12C compares enriched Reactome pathways of TCMR with those of BKVN-P.

Altogether, TCMR samples showed robust enrichment of TCR signaling, CD28 costimulation, innate immune response, GTPase-mediated signaling cascade, integrin-mediated interactions, immune cell infiltration, and extracellular matrix remodeling. Negatively enriched pathways suggested downregulation of homeostatic transport and epithelial barrier maintenance. BKVN-P samples revealed strong innate immune responses, chemokine-driven immune cell recruitment, IFN and cytokine signaling characteristic of viral infections, and suppression of epithelial and metabolic homeostasis — likely reflecting inflammation and virus-induced tissue damage.

**Figure 13:**
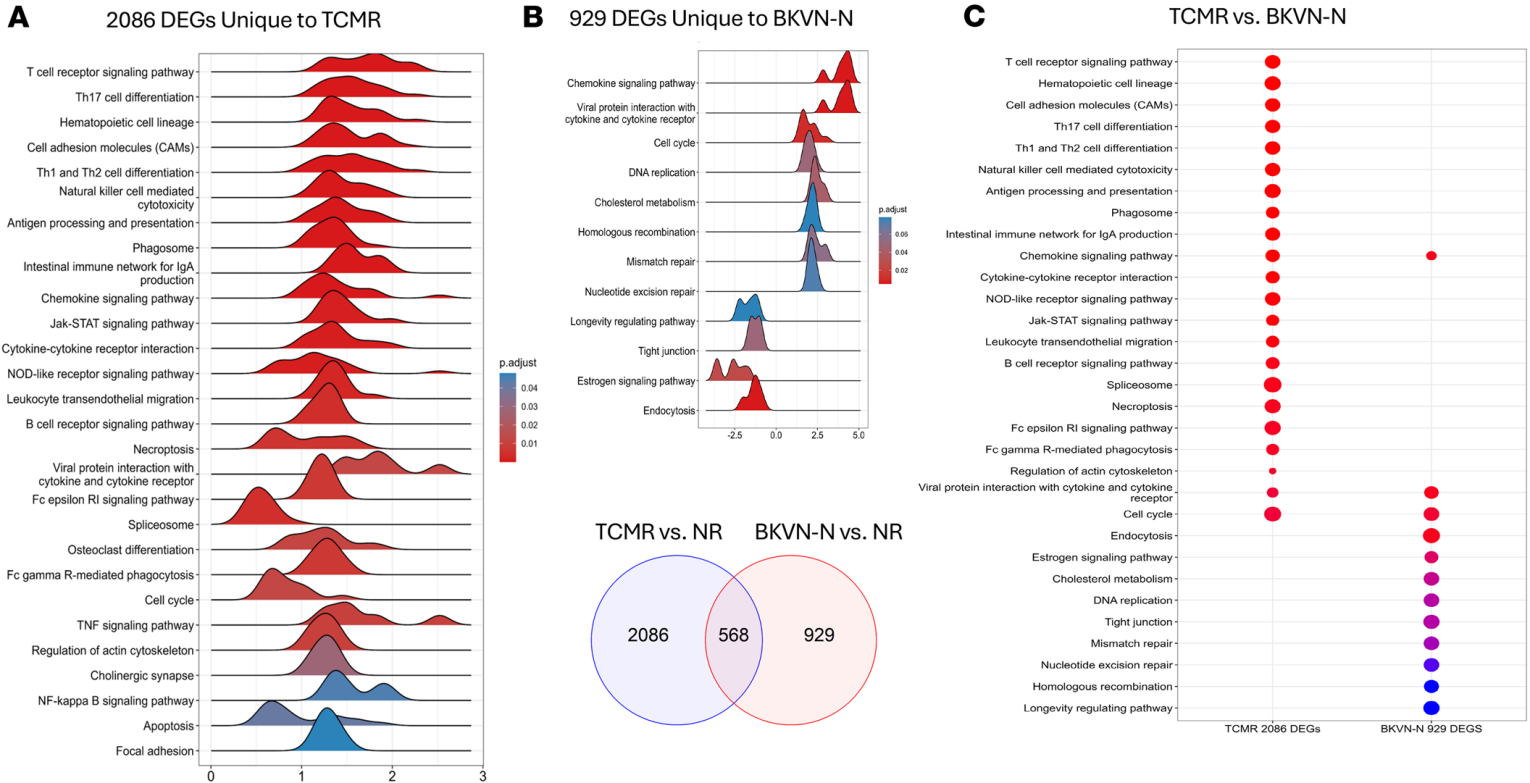
GSEA of KEGG pathways uniquely enriched in BKVN-N urines relative to TCMR urines. (**A**) Ridge plot showing enriched KEGG pathways (adjusted *P* < 0.05) based on the 2,086 DEGs unique to TCMR (Supplemental Table 34). (**B**) Ridge plot showing enriched KEGG pathways (adjusted *P* < 0.05) based on 929 DEGs unique to BKVN-N (Supplemental Table 35). (**C**) clusterProfiler comparison of Reactome pathways profile enriched in TCMR and BKVN-N by GSEA.

We also compared the 2,654 DEGs identified by comparison of TCMR versus NR with the 1,497 DEGs identified by comparison of BKVN-N versus NR samples. This revealed 568 shared DEGs (Supplemental Table 31), 2,086 DEGs unique to TCMR (Supplemental Table 32), and 929 DEGs unique to BKVN-N (Figure 13, Venn diagram; and Supplemental Table 33).

**Figure 14:**
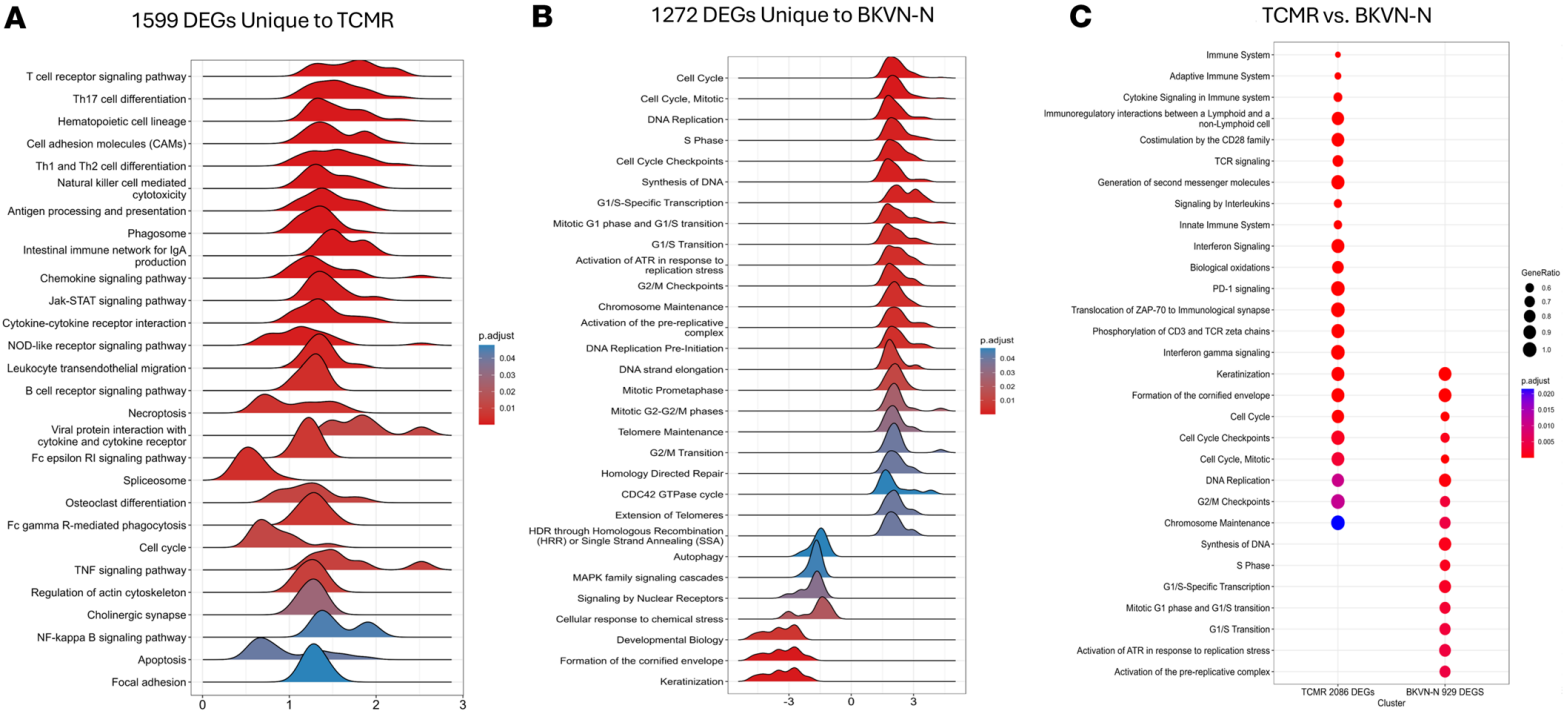
GSEA of Reactome pathways uniquely enriched in BKVN-N samples compared with TCMR transcriptomes. (**A**) Ridge plot of enriched Reactome pathways (adjusted *P* < 0.05) based on the 2,086 DEGs unique to TCMR (Supplemental Table 36). (**B**) Ridge plot of enriched Reactome pathways (adjusted *P* < 0.05) based on 929 DEGs unique to BKVN-N (Supplemental Table 37). (**C**) clusterProfiler comparison of Reactome pathways enriched in TCMR and BKVN-N by GSEA.

Figure 13A presents ridge plot distributions of enriched KEGG pathways based on the 2,086 DEGs unique to TCMR (Supplemental Table 34). Figure 13B displays enriched KEGG pathways based on the DEGs unique to BKVN-N (Supplemental Table 35). Figure 13C compares the enriched KEGG pathways. Figure 14A presents enriched Reactome pathways based on the DEGs unique to TCMR (Supplemental Table 36). Figure 14B presents enriched Reactome pathways based on the DEGs unique to BKVN-N (Supplemental Table 37). Figure 14C compares the enriched Reactome pathways.

The positively enriched pathways in TCMR samples reflected strong T cell activation and proliferation, cytokine signaling (including IFN signaling), innate immune cell recruitment, and inflammation. Negatively enriched pathways indicated suppression of tissue-specific metabolic and structural functions.

The positively enriched pathways in BKVN-N samples were consistent with viral hijacking of host replication machinery, activation of DNA damage response and repair, replication stress, suppression of epithelial cell differentiation and signaling, loss of epithelial barrier integrity, and altered lipid and hormone signaling.

The comparative analysis showed that CD28 costimulation was dominant in TCMR and virtually absent in BKVN-N. Conversely, BKVN-N samples showed stronger enrichment of viral protein interaction pathways, virus-driven host cell proliferation, replication stress, and checkpoint activation and suppression of epithelial differentiation and signaling pathways.

### Noninvasive biomarker for discriminating BKVN from TCMR.

Urinary cell transcriptome analyses identified that several chemokine mRNAs were markedly overexpressed in BKVN samples compared with both NR and TCMR samples (Supplemental Table 9A). Conversely, expression profiles of mRNA for T cell signaling proteins, overexpressed in TCMR, were not overexpressed in either BKVN-N or BKVN-P (Supplemental Table 10C). The contrasting patterns — T cell dominance in TCMR and chemokine enrichment in BKVN — led us to investigate whether a ratio of a chemokine such as CXCL10 mRNA to T cell CD3E mRNA could distinguish BKVN from TCMR.

### Urinary cell CXCL10 mRNA abundance is diagnostic of TCMR, and the CXCL10 mRNA/CD3E mRNA ratio differentiates BKVN from TCMR.

We quantified CXCL10 mRNA and CD3E mRNA levels in urines matched to kidney allograft biopsies, calculated the ratio of CXCL10 mRNA to CD3E mRNA, and assessed the ability of these measures to distinguish diagnostic categories. Specifically, we assessed their performance as biomarkers of TCMR and their utility in differentiating TCMR from BKVN.

### Discovery cohort (58 urine samples matched to 57 biopsies), RNA-seq quantification.

**Figure 15:**
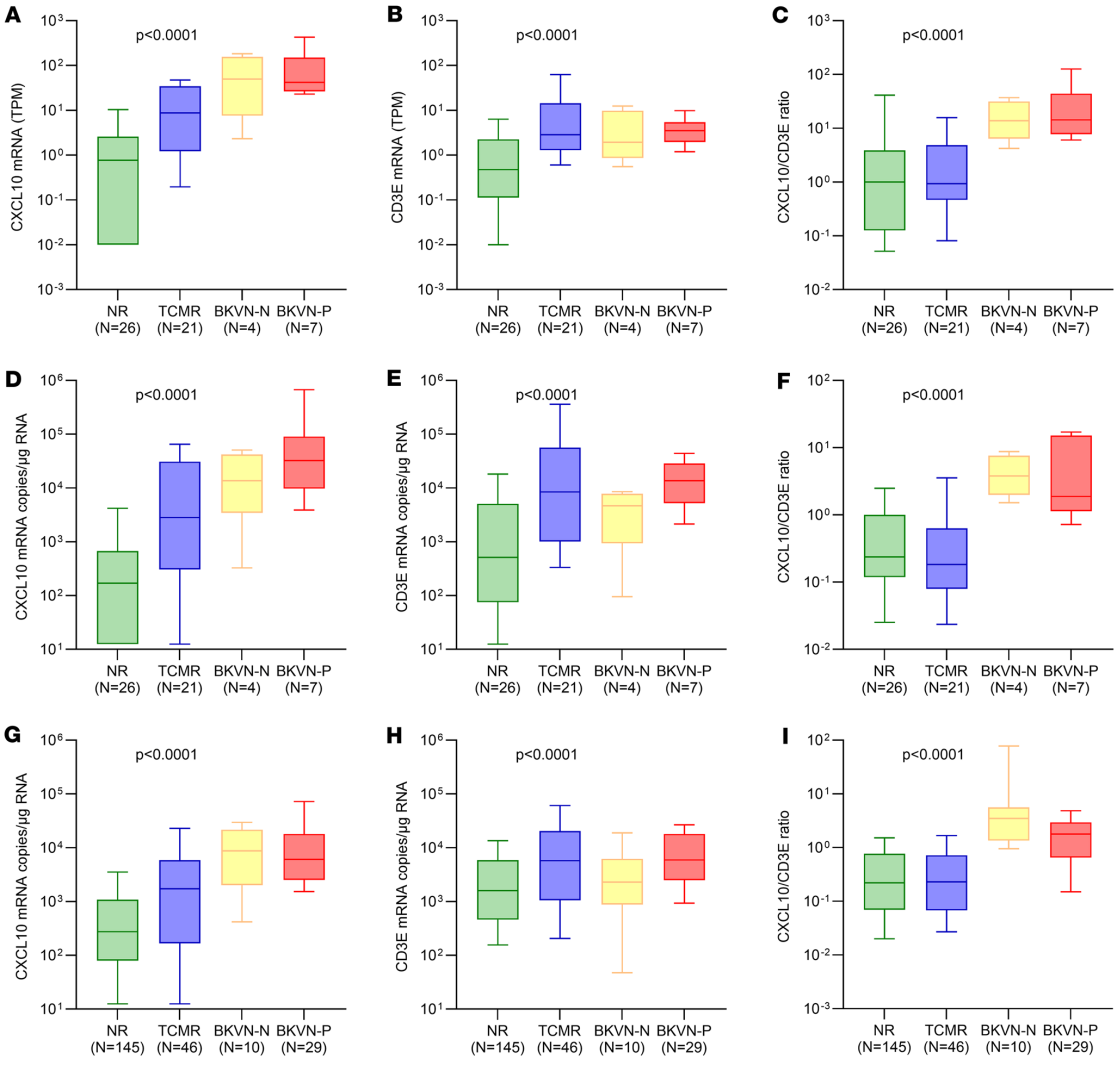
Box-and-whisker plots of CXCL10 mRNA, CD3E mRNA, and CXCL10 mRNA to CD3E mRNA ratios across diagnostic categories and across discovery and external validation cohorts. (**A**–**C**) Discovery cohort (*n* = 58 urine samples matched to 57 biopsies), RNA-seq quantification. Box-and-whisker plots show urinary cell CXCL10 mRNA (**A**) and CD3E mRNA (**B**) expression (TPM), and the CXCL10 mRNA/CD3E mRNA ratio (**C**). Boxes denote the 25th–75th percentiles, the horizontal line marks the median, and whiskers represent the 10th–90th percentiles. Each point represents the mRNA values (**A** and **B**) or the ratio (**C**) for each sample. (**D**–**F**) Discovery cohort (*n* = 58 urine samples matched to 57 biopsies), customized RT-qPCR quantification. Box-and-whisker plots show absolute copy numbers of urinary cell CXCL10 mRNA (**D**) and CD3E mRNA (**E**) per microgram of total RNA, and the CXCL10 mRNA to CD3E mRNA ratio (**F**). Each point represents the mRNA values (**D** and **E**) or the ratio (**F**) for each sample. (**G**–**I**) External validation cohort (*n* = 230 urine samples matched to 230 biopsies), customized RT-qPCR quantification. Box-and-whisker plots show absolute copy numbers of urinary cell CXCL10 mRNA (**G**) and CD3E mRNA (**H**) per microgram of total RNA, and the ratio of CXCL10 mRNA to CD3E mRNA (**I**). Each point represents the mRNA values (**D** and **E**) or the ratio (**F**) for each sample. Kruskal-Wallis test *P* values are shown at the top of each panel. Dunn’s test was used for pairwise comparisons. Numbers in parentheses indicate the number of matched urine-biopsy pairs except for BKVN-P (7 urines matched to 6 biopsies).

The median [IQR] of CXCL10 transcripts per million (TPM) was 0.8 [2] in the 26 NR biopsy-matched urines, 8.8 [30] in 21 TCMR biopsy-matched urines, 50.0 [85] in 4 BKVN-N biopsy-matched urines, and 42.0 [69] in 7 BKVN-P urines matched to 6 BKVN-P biopsies (Kruskal-Wallis [KW] test, χ^2^ = 26.3, df = 3, *P* < 0.0001) (post hoc Dunn’s test *P*: NR vs. TCMR, *P* = 0.0069; NR vs. BKVN-N, *P* = 0.009; NR vs. BKVN-P, *P* < 0.0001; TCMR vs. BKVN-N, *P* = 0.5993; TCMR vs. BKVN-P, *P* = 0.0737; and BKVN-N vs. BKVN-P, *P* = 1) (Figure 15A).

CD3E TPM was 0.48 [1.75] in NR urines, 2.85 [7.92] in TCMR urines, 1.94 [3.17] in BKVN-N urines, and 3.53 [2.36] in BKVN-P urines (KW test, χ^2^ = 16.2, df = 3, *P* < 0.0001) (Dunn’s test *P*: NR vs. TCMR, *P* = 0.0006; NR vs. BKVN-N, *P* = 0.5591; NR vs. BKVN-P, *P* = 0.0318; TCMR vs. BKVN-N, *P* = 1; TCMR vs. BKVN-P, *P* = 1; and BKVN-N vs. BKVN-P, *P* = 1) (Figure 15B).

CXCL10 TPM/CD3E TPM ratio was 1.0 [2.71] in NR urines, 0.93 [2.77] in TCMR urines, 13.78 [9.50] in BKVN-N urines, and 14.30 [22.29] in BKVN-P urines (KW test, χ^2^ = 13.57, df = 3, *P* < 0.0001) (Dunn’s test *P*: NR vs. TCMR, *P* = 1; NR vs. BKVN-N, *P* = 0.094; NR vs. BKVN-P, *P* = 0.0093; TCMR vs. BKVN-N, *P* = 0.0918; TCMR vs. BKVN-P, *P* = 0.0098; and BKVN-N vs. BKVN-P, *P* = 1) (Figure 15C).

### Discovery cohort (58 urine samples matched to 57 biopsies), customized RT-qPCR quantification.

We used an orthogonal quantitative reverse transcription PCR (RT-qPCR) assay to measure absolute copy numbers of CXCL10 mRNA and CD3E mRNA in biopsy-matched urine samples. In the RNA-seq cohort (discovery set), the median [IQR] CXCL10 mRNA copy number was 170 [636] per microgram of total RNA in 26 NR urines, 2,830 [27,236] in 21 TCMR urines, 13,650 [13,793] in 4 BKVN-N urines, and 32,500 [52,350] in 7 BKVN-P urines matched to 6 BKVN-P biopsies (KW test, χ^2^ = 22.2, df = 3, *P* < 0.0001) (Dunn’s test *P*: NR vs. TCMR, *P* = 0.0102; NR vs. BKVN-N, *P* = 0.0452; NR vs. BKVN-P, *P* = 0.0001; TCMR vs. BKVN-N, *P* = 1; TCMR vs. BKVN-P, *P* = 0.1109; and BKVN-N vs. BKVN-P, *P* = 1) (Figure 15D).

CD3E mRNA copy number was 512 [4,968] in NR urines, 8,450 [50,910] in TCMR urines, 4,655 [3,884] in BKVN-N urines, and 13,600 [21,620] in BKVN-P urines (KW test, χ^2^ = 15.3, df = 3, *P* < 0.0001) (Dunn’s test *P*: NR vs. TCMR, *P* = 0.0017; NR vs. BKVN-N, *P* = 1; NR vs. BKVN-P, *P* = 0.0128; TCMR vs. BKVN-N, *P* = 0.982; TCMR vs. BKVN-P, *P* = 1; and BKVN-N vs. BKVN-P, *P* = 0.7169) (Figure 15E).

The CXCL10 mRNA copies/CD3E mRNA copies ratio was 0.24 [0.87] in NR urines, 0.18 [0.42] in TCMR urines, 3.79 [2.35] in BKVN-N urines, and 1.88 [8.31] in BKVN-P urines (KW test, χ^2^ = 18.49, df = 3, *P* < 0.0001) (Dunn’s test *P*: NR vs. TCMR, *P* = 1; NR vs. BKVN-N, *P* = 0.0224; NR vs. BKVN-P, *P* = 0.0081; TCMR vs. BKVN-N, *P* = 0.0086; TCMR vs. BKVN-P, *P* = 0.0023; and BKVN-N vs. BKVN-P, *P* = 1) (Figure 15F).

### External validation cohort (230 urine samples matched to 230 biopsies), customized RT-qPCR quantification.

Demographics, biopsy findings, and total RNA characteristics of the external validation cohort are summarized in Supplemental Table 38, A–C, respectively.

In the external validation set, the median [IQR] CXCL10 mRNA copy number per microgram of total RNA was 274 [950] in 145 NR urines, 1,734 [5,492] in 46 TCMR urines, 8,785 [17,768] in 10 BKVN-N urines, and 6,097 [14,961] in 29 BKVN-P urines (KW test, χ^2^ = 62.8, df = 3, *P* < 0.0001) (Dunn’s test *P*: NR vs. TCMR, *P* = 0.0013; NR vs. BKVN-N, *P* = 0.0001; NR vs. BKVN-P, *P* < 0.0001; TCMR vs. BKVN-N, *P* = 0.0849; TCMR vs. BKVN-P, *P* = 0.002; and BKVN-N vs. BKVN-P, *P* = 1) (Figure 15G).

CD3E mRNA copy number was 1,594 [5,292] in NR urines, 5,772 [17,514] in TCMR urines, 2,301 [4,202] in BKVN-N urines, and 5,927 [14,936] in BKVN-P urines (KW test, χ^2^ = 19.9, df = 3, *P* < 0.0001) (Dunn’s test *P*: NR vs. TCMR, *P* = 0.0015; NR vs. BKVN-N, *P* = 1; NR vs. BKVN-P, *P* = 0.0019; TCMR vs. BKVN-N, *P* = 1; TCMR vs. BKVN-P, *P* = 1; and BKVN-N vs. BKVN-P, *P* = 1) (Figure 15H).

The CXCL10 mRNA to CD3E mRNA ratio was 0.22 [0.69] in NR urines, 0.23 [0.59] in TCMR urines, 3.49 [3.67] in BKVN-N urines, and 1.79 [2.14] in BKVN-P urines (KW test, χ^2^ = 48.33, df = 3, *P* < 0.0001) (Dunn’s test *P*: NR vs. TCMR, *P* > 0.9999; NR vs. BKVN-N, *P* < 0.0001; NR vs. BKVN-P, *P* < 0.0001; TCMR vs. BKVN-N, *P* < 0.0001; TCMR vs. BKVN-P, *P* < 0.0001; and BKVN-N vs. BKVN-P, *P* = 1) (Figure 15I).

Altogether, our data demonstrate that the expression levels of CXCL10 mRNA (and CD3E mRNA) discriminate TCMR from NR, consistent with earlier reports that CXCL10 mRNA or protein discriminates NR from TCMR (53–55). The current study identified that the ratio of CXCL10 mRNA to CD3E mRNA discriminates accurately BKVN-N from TCMR, BKVN-P from TCMR, and BKVN from TCMR.

### Receiver operating characteristic curves for discriminating BKVN from TCMR.

We performed receiver operating characteristic (ROC) curve analyses across the discovery set, the external validation set, and the combined data set to evaluate the diagnostic performance of the ratio of CXCL10 mRNA to CD3E mRNA for discriminating BKVN-N from TCMR, BKVN-P from TCMR, and BKVN from TCMR.

**Figure 16:**
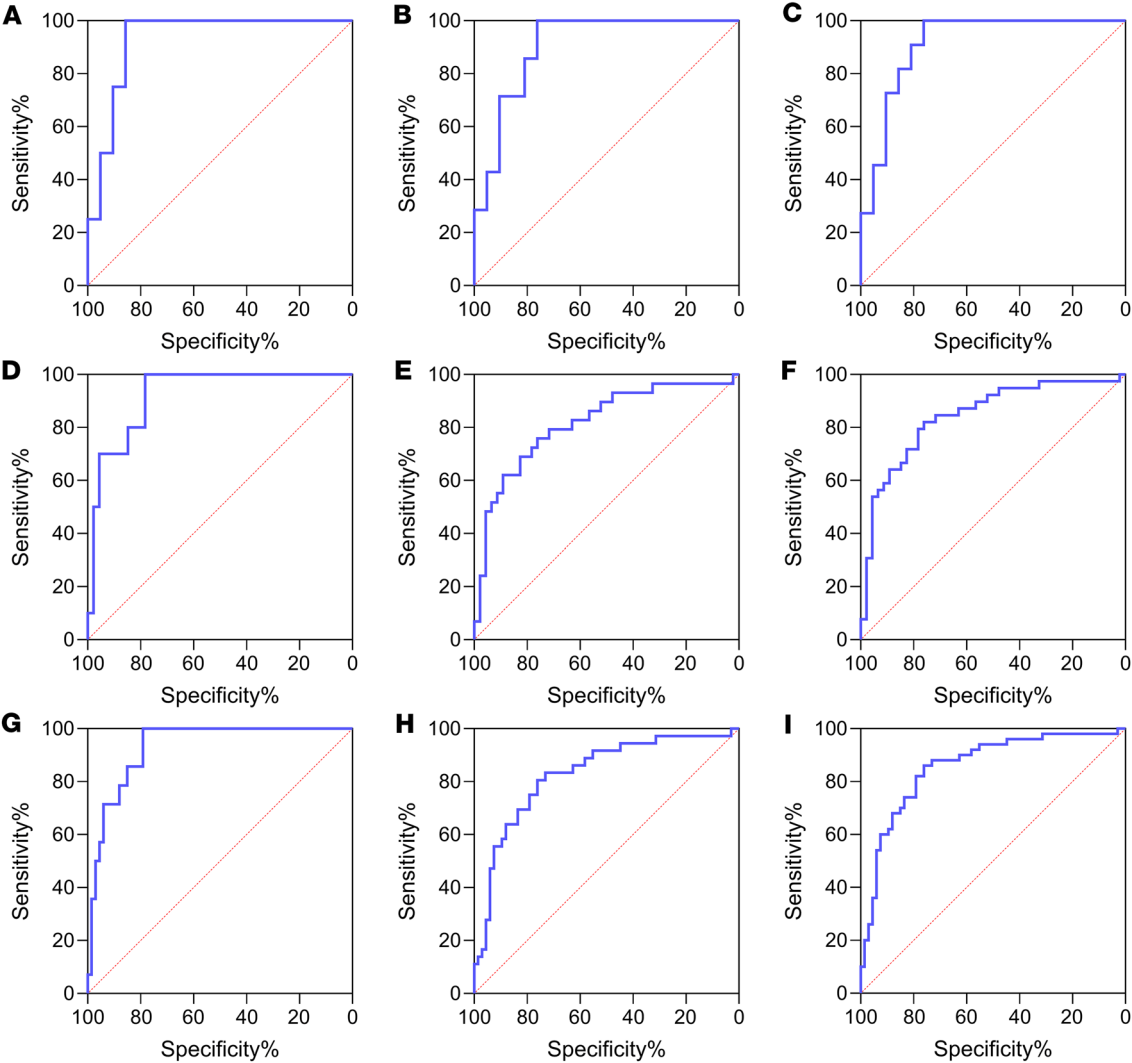
ROC curves for distinguishing BKVN-N from TCMR, BKVN-P from TCMR, and BKVN from TCMR across discovery, external validation, and combined cohorts. (**A**–**C**) Discovery cohort (*n* = 32 urines matched to biopsies). (**A**) BKVN-N (*n* = 4) vs. TCMR (*n* = 21), AUROC = 0.93. (**B**) BKVN-P (*n* = 7) vs. TCMR (*n* = 21), AUROC = 0.9. (**C**) BKVN (*n* = 11) vs. TCMR (*n* = 21), AUROC = 0.91. (**D**–**F**) External validation set (*n* = 85 urine samples matched to biopsies). (**D**) BKVN-N (*n* = 10) vs. TCMR (*n* = 46), AUROC = 0.92. (**E**) BKVN-P (*n* = 29) vs. TCMR (*n* = 46), AUROC = 0.82. (**F**) BKVN (*n* = 39) vs. TCMR (*n* = 46), AUROC = 0.84. (**G**–**I**) Combined cohort (*n* = 117 urine samples matched to biopsies). (**G**) BKVN-N (*n* = 14) vs. TCMR (*n* = 67), AUROC = 0.93. (**H**) BKVN-P (*n* = 36) vs. TCMR (*n* = 67), AUROC = 0.83. (**I**) BKVN (*n* = 50) vs. TCMR (*n* = 67), AUROC = 0.85. ROC curves illustrate sensitivity and specificity. An AUROC of 1.0 indicates perfect classification, whereas 0.5 reflects random performance.

Figure 16 illustrates ROC curves for BKVN-N versus TCMR, BKVN-P versus TCMR, and BKVN versus TCMR within each data set. In the discovery set, discrimination between BKVN-N and TCMR achieved an area under the ROC curve (AUROC) of 0.93 (95% confidence interval [CI], 0.83–1, *P* = 0.002; Figure 16A), while BKVN-P versus TCMR yielded an AUROC of 0.9 (95% CI, 0.79–1, *P* = 0.0004; Figure 16B) and all BKVN cases versus TCMR an AUROC of 0.91 (95% CI, 0.82–1, *P* = 1.96 × 10^–5^; Figure 16C).

In the external validation set, AUROC values were 0.92 (95% CI, 0.85–1, *P* = 1.42 × 10^–6^) for BKVN-N versus TCMR (Figure 16D), 0.82 (95% CI, 0.71–0.92, *P* = 7.8 × 10^–7^) for BKVN-P versus TCMR (Figure 16E), and 0.84 (95% CI, 0.76–0.93, *P* = 4.05 × 10^–9^) for all BKVN cases versus TCMR (Figure 16F).

In the combined set, the AUROC values remained robust: 0.93 (95% CI, 0.88–0.99, *P* = 2.3 × 10^–7^) for BKVN-N versus TCMR (Figure 16G), 0.83 (95% CI, 0.74–0.92, *P* = 2.03 × 10^–8^) for BKVN-P versus TCMR (Figure 16H), and 0.85 (95% CI, 0.78–0.92, *P* = 1.52 × 10^–10^) for all cases of BKVN versus TCMR (Figure 16I). The optimal cut point, which maximized the combined sensitivity and specificity, was 0.67. At this threshold, the CXCL10 mRNA/CD3E mRNA ratio distinguished BKVN from TCMR with a sensitivity of 85% and a specificity of 76%.

Collectively, our findings support the utility of the ratio of CXCL10 mRNA to CD3E mRNA as a noninvasive biomarker capable of distinguishing BKVN-N (without intragraft inflammation) and BKVN-P (with intragraft inflammation) from TCMR, which is characterized by prominent intragraft inflammation.

### Transcriptomic signature discriminating TCMR from BKVN.

**Figure 17:**
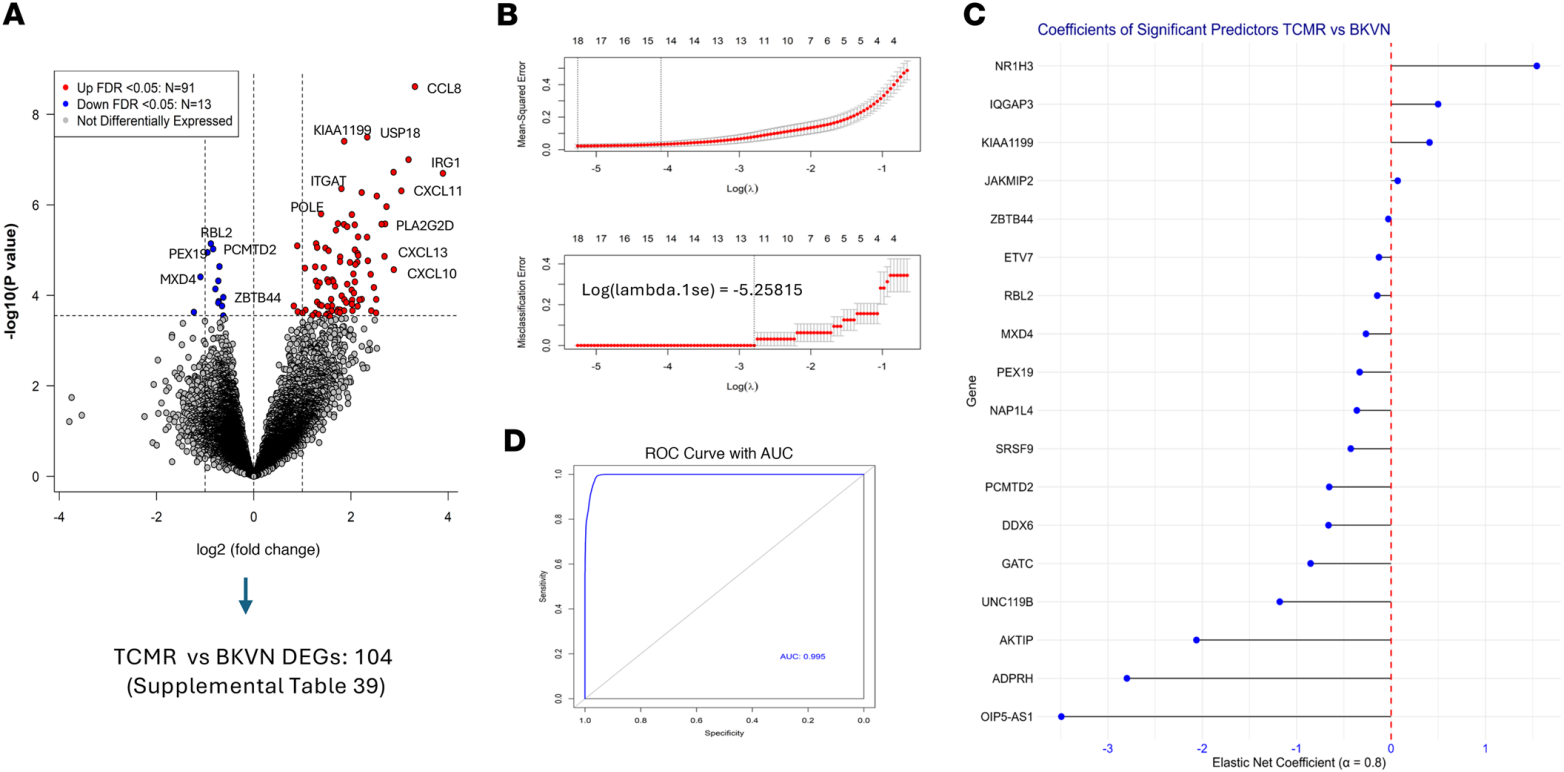
Transcriptomic signature discriminating TCMR from BKVN. (**A**) Twenty-one urinary cell transcriptomes matched to 21 TCMR biopsies were compared with 11 urinary cell transcriptomes matched to 10 BKVN biopsies (4 BKVN-N and 6 BKVN-P). The volcano plots show DEGs (log_2_ fold change vs. log_10_ *P*). Supplemental Table 39 lists 104 DEGs. (**B**) glmnet cross-validation plots display mean-squared and misclassification error across λ values, with vertical lines indicating the optimal penalties (minimum error and more parsimonious 1-SE solution). (**C**) Elastic net coefficient plot for each of the 18 predictor genes shows coefficient direction and magnitude in the model that perfectly separates TCMR from BKVN (AUROC = 1.0). Supplemental Table 40 lists the 18 genes and their elastic net coefficients, random forest validation metrics, and TCMR versus BKVN fold changes and adjusted *P* values. (**D**) ROC curve for a bootstrapped random forest classifier trained on the selected genes (*N* = 1,000 bootstraps; caret with optimal mtry = 2) shows high performance accuracy of 0.973 (95% CI, 0.970–0.975) and AUROC of 0.995.

To define a transcriptome-wide signature that distinguishes TCMR from BKVN, 21 urinary cell transcriptomes matched to 21 TCMR biopsies were compared with 11 urinary cell transcriptomes matched to 10 BKVN biopsies (4 BKVN-N urine samples matched to 4 BKVN-N biopsies and 7 urine samples matched 6 BKVN-P biopsies). Figure 17A shows a volcano plot summarizing DEGs. This analysis identified 104 DEGs, with 91 overexpressed (adjusted *P* < 0.05) and 13 underexpressed (adjusted *P* < 0.05) (Supplemental Table 39).

Elastic net regression was then used for robust feature selection. The cv.glmnet function (https://cran.r-project.org/web/packages/glmnet/index.html) identified the optimal λ by cross-validation, selecting the model with the minimum error of λ at α = 0.8. Figure 17B displays the glmnet cross-validation plot, which shows mean-squared and misclassification error across λ values; vertical lines indicate the optimal penalties (minimum error and more parsimonious 1-SE solution).

Shrinkage of the 104 DEGs identified an 18-gene panel that perfectly discriminated TCMR from BKVN with an accuracy of 100% (95% CI, 0.89–1.0) and an AUROC of 1.0. A bootstrap evaluation of the 18-gene model with 1,000 iterations confirmed the high performance, yielding an accuracy of 0.987 (95% CI, 0.984–0.989) and an AUROC of 0.999.

Figure 17C shows the elastic net coefficient plot, which depicts the estimated coefficients for each of the 18 gene predictors, illustrating their direction and relative contribution to the model that separates TCMR from BKVN (AUROC = 1.0). Supplemental Table 40 lists the 18 predictor genes and their elastic net coefficients, random forest validation, and TCMR versus BKVN fold changes for the 18 predictors, and the adjusted *P* values for the fold changes.

To evaluate model robustness, a bootstrapped random forest was fitted using the selected genes. Using *N* = 1,000 bootstrap samples and the caret package train function with the optimal mtry = 2 (https://cran.r-project.org/web/packages/caret/index.html), this model achieved similarly high performance, with an accuracy of 0.973 (95% CI, 0.970–0.975) and an AUROC of 0.995 (Figure 17D).

## Discussion

This study employed transcriptomic profiling of urinary cells from kidney allograft recipients representing three key diagnostic groups: BKVN, TCMR, and NR. Ensemble RNA-seq and curated pathway analyses delineated distinct programs in BKVN with intragraft inflammation (BKVN-P) and BKVN without intragraft inflammation (BKVN-N) and contrasted to TCMR.

BK polyoma virus exploits host DNA replication, repair, and cell cycle machinery to propagate (29). Our transcriptomic data identified in vivo upregulation of genes essential for BKVN replication (27, 56). Concomitant downregulation of cell cycle inhibitors indicated impaired host regulation. Mitochondrial transcript upregulation and viral agnoprotein disruption of mitochondrial energy gradient were notable (57). BKVN samples displayed microtubule activation and cytoskeletal remodeling; genes for intracellular transport and vesicular trafficking were underexpressed in both BKVN subtypes.

Antigen presentation and innate immune pathways marked BKVN versus TCMR. Both BKVN-N and BKVN-P showed robust pattern recognition receptor and HLA class II activation, with BKVN-P more expansive. These immune differences manifested at the chemokine/cytokine level, with BKVN-P upregulating proinflammatory mediators while downregulating certain homeostatic chemokines, indicating loss of tissue repair programs.

BKVN urinary cell transcriptomes highlighted T cell exhaustion signatures but not anergy (58, 59). T cell exhaustion was consistent across BKVN subtypes, unlike TCMR’s activation-centric signature. Cytolytic CD4^+^ T cell activation and immunosuppressive mediators like IL4I1, regulating immunopathology and viral persistence, were elevated in BKVN-P, while subset analysis showed antiviral Th1 response in BKVN-N and T regulatory/cytotoxic activity in BKVN-P.

B cell activation programs were found in BKVN-P, and anergy/exhaustion profiles in BKVN-N, shaping immune dynamics. Both mature and tolerogenic cell signatures were increased in BKVN, especially BKVN-P, indicating simultaneous immune activation and tolerance.

Integrative pathway analysis distinguished BKVN from TCMR at the molecular level. TCMR transcriptomes were marked by TCR and CD28 proinflammatory signaling, whereas BKVN-P featured innate and viral response modules and epithelial dysfunction.

Urinary transcriptomes enabled noninvasive discrimination of BKVN subtypes from TCMR. Our discovery of a ratiometric transcript-based classifier — the CXCL10 mRNA/CD3E mRNA ratio — represents a distinct advance over existing approaches for differentiating BKVN from TCMR. By pairing a chemokine signal disproportionately amplified in BKVN with a T cell–lineage signal enriched in TCMR, this strategy mitigates confounding from global inflammation and intersample variability, thus enhancing analytical stability and clinical interpretability across platforms. The ratios’ replication by RNA-seq and customized RT-qPCR in the same sample, together with external validation in an independent, biopsy-matched urine cohort, underscores technical robustness and translational readiness. Importantly, its ability to distinguish both BKVN without intragraft inflammation (BKVN-N) and BKVN with inflammation (BKVN-P) from TCMR, while not separating BKVN subtypes, indicates specificity for etiologic discrimination rather than histologic gradation — an attribute desirable for guiding therapy. These features position the CXCL10 mRNA/CD3E mRNA ratio to reduce misclassification-driven management errors, such as unwanted intensification of immunosuppression in BKVN, and to serve as a noninvasive adjunct or, in select instances, an alternative to biopsy for real-time decision-making in kidney transplant care.

In the current investigation, we confirm and extend prior observations that CXCL10 abundance discriminates NR from TCMR (54, 55). A plausible mechanistic basis for the pronounced increase in CXCL10 mRNA (and other chemokines) is activation of innate immune pathways triggered by BK viral DNA.

Urinary cell transcriptomics sharply discriminated TCMR from BKVN, and yielded a concise, mechanistically plausible gene signature, capturing the molecular divergence between alloimmune and BK virus–driven injury. Discovery analysis identified 104 DEGs between TCMR and BKVN, from which an 18-gene elastic net panel achieved perfect separation of the 2 diagnoses (AUROC = 1.0) with internal cross-validation support. A bootstrapped random forest using these genes showed similarly high accuracy (AUROC = 0.995), indicating that the signal is not model specific. Collectively, these data support a robust, low-dimensional urinary transcriptomic signature that may enable noninvasive discrimination of TCMR from BKVN, and merits refinement and validation in larger prospective cohorts.

Potential limitations include single-center design, batch effects, and modest sample size. Notably, no sequencing batch was unique to any diagnostic group (Supplemental Table 41), with multidimensional scaling plots and *K*-function analysis confirming minimal batch confounding (Supplemental Figures 2 and 3). BKVN-N samples exceeded RNA-seq replication standards (60), with mean Pearson’s correlations of 0.9257 (Supplemental Table 42) confirming technical and biological reproducibility and surpassing the ENCODE benchmark (61) of 0.90 for biological replicability. Our cross-sectional design precluded temporal insights. Prospective, multicenter studies are required for further refinement and clinical translation.

In sum, our in-depth transcriptomic analyses reveal profound and divergent reprogramming of host and immune pathways in urinary cells of kidney allograft recipients with BKVN, offering mechanistic insights into viral pathogenesis, immune dysregulation, and potential diagnostic signatures to differentiate BKVN from allograft rejection. These findings set the stage for new molecular approaches to noninvasive diagnosis, risk stratification, and tailored therapy in the care of kidney transplant recipients.

## Methods

Additional details are provided in Supplemental Methods and Results.

### Sex as a biological variable.

We studied 53 kidney transplant recipients (RNA-seq cohort) — 30 females and 23 males, given transplants and followed at our center, New York-Presbyterian/Weill Cornell Medicine. We also studied 238 kidney transplant recipients (external validation cohort) — 74 females and 152 males, given transplants and followed at our center.

### Urine collection, RNA isolation, and sequencing.

About 50 mL of urine per patient was collected and processed, as previously described (18). Urinary cell RNA was rRNA-depleted (13); libraries were pooled and sequenced (Illumina HiSeq4000, paired-end 100-cycle reads).

Supplemental Table 1, A–C, is a summary of the RNA-seq cohort, and Supplemental Table 38, A–C, is a summary of the external validation cohort.

### Timing of urine collection relative to kidney allograft biopsy.

Supplemental Table 43, A and B, summarizes the timing of collection for the 58 urines matched to 57 biopsies in the RNA-seq cohort, and for 230 urine-biopsy pairs in the external validation cohort, respectively.

### Timing of plasma collection relative to kidney allograft biopsy.

Supplemental Table 44, A and B, provides timing of plasma collection relative to the timing of biopsy in the RNA-seq cohort and the external validation cohort, respectively.

### Kidney allograft biopsies.

Biopsies were evaluated by a senior renal pathologist using the Banff 2019 update (62) of the Banff 1997 classification of allograft pathology (63).

### Differential gene expression analysis.

Reads were aligned to GRCh38/hg38 using the STAR aligner (v2.4.2) (64) and quantified via CuffLinks (v2.2.1) (65) and HTSeq (66); Fragments per kilobase of transcript per million mapped reads (FPKM) values were converted to transcripts per million (TPM). Limma-voom in R (23, 67) was used for differential expression analysis. DEGs were annotated via clusterProfiler (68), KEGG 2016 (69), Enrichr (70), and Reactome (71), with FDR < 0.05 for significance.

GSEA (72) and pathway analysis (R package clusterProfiler, version 4.14.6) (73) compared gene expression profiles across diagnostic categories and to identify enriched biological functions. In addition to identifying functions of DEGs, using *t*-statistics and pre-ranked GSEA, enrichment score indicated maximum deviation from zero for enrichment (72).

### Batch effect and biological reproducibility.

Samples were processed in 3 batches; no batch was associated with any diagnostic category (Supplemental Table 41). Replicability was assessed with Pearson’s correlations (Supplemental Table 42).

### Customized RT-qPCR assays.

Total RNA was reverse-transcribed to cDNA (TaqMan, Applied Biosystems). Primers/probes were designed using Primer Express software (Thermo Fisher Scientific) for target mRNAs and 18S rRNA (18). BK polyomavirus VP1 mRNA was quantified as previously described (43). A 2-step RT-qPCR assay measured CXCL10 mRNA and CD3E mRNA, with quantification on QuantStudio 6 Flex (Thermo Fisher Scientific) (17).

### Statistics.

The limma package in R (http://bioconductor.org/packages/release/bioc/html/limma.html), applied to voom counts, was used for differential expression. FDR was controlled for by Benjamini-Hochberg adjustment. Kolmogorov-Smirnov test compared CDFs across groups and gene sets. Gene-set-level changes were analyzed using ROAST (45) and CAMERA (46) in limma R. ROC curve analysis evaluated diagnostic performance of ratio of CXCL10 mRNA to CD3E mRNA. Pairwise comparisons were performed using the Mann–Whitney U test. Multiple-group comparisons were evaluated using Kruskal-Wallis test and Dunn’s test for post hoc analysis. *P* < 0.05 was considered significant.

### Study approval.

Recipients gave written informed consent prior to inclusion. The IRB at Weill Cornell Medicine approved the study. Our clinical and research activities aligned with the Declaration of Istanbul (74) and the World Medical Association Declaration of Helsinki (75).

### Data availability.

We have deposited the urinary cell RNA sequence data in the NCBI’s Gene Expression Omnibus (accession numbers GSE142667 and GSE312597).

## Author contributions

FBM and MS designed research studies. CL, JZX, and VKS conducted experiments. FBM, CL, DMD, SVS, TS, and VKS acquired data. FBM, CL, and MS analyzed data. VKS and JZX provided reagents. FBM, HHH, TM, and MS wrote the manuscript.

## Conflict of interest

The authors have declared that no conflict of interest exists.

## Funding support

This work is the result of NIH funding, in whole or in part, and is subject to the NIH Public Access Policy. Through acceptance of this federal funding, the NIH has been given a right to make the work publicly available in PubMed Central.

- MERIT Award, National Institute of Allergy and Infectious Diseases, NIH, R37AI051652, to MS.
- K08 Award, National Institute of Diabetes and Digestive and Kidney Diseases, NIH, DK087824, to TM.
- Clinical and Translational Science Center Award, NIH, UL1TR000457, to Weill Cornell Medical College.

## Supplementary Material

Supplemental data

Supplemental tables 1-44

Supporting data values

## Version 1 06/08/2026

Electronic publication
