# Supplementary material for: Benchmarking urinary cell transcriptomes for noninvasive differentiation of BK polyomavirus–associated nephropathy from T cell–mediated rejection
Source: JCI Insight. 2026 Jun 8;11(11):e201060. doi: 10.1172/jci.insight.201060 (PMC13313547; doi:10.1172/jci.insight.201060)
Supplement: Supplemental data [file jciinsight-11-201060-s338.pdf]

Benchmarking urinary cell transcriptomes for noninvasive differentiation of BK polyomavirus-associated nephropathy from T cell-mediated rejection

Franco B. Mueller,[1] Carol Li,[1] Darshana M. Dadhania,[1,2]
Surya V. Seshan,[3] Thalia Salinas,[1,2] Vijay K. Sharma,[1] Jenny Z. Xiang,[4]
Hans H. Hirsch,[5] Thangamani Muthukumar,[1,2] Manikkam Suthanthiran[1,2]

[1]Division of Nephrology and Hypertension, Department of Medicine, Weill Cornell Medicine, New York, NY, USA.
[2]Department of Transplantation Medicine, NewYork Presbyterian Hospital, New York, NY, USA.

[3]Division of Renal Pathology, Department of Pathology and Laboratory Medicine, Weill Cornell Medicine, New York, NY, USA.

[4]Genomics Resources Core Facility, Department of Microbiology and Immunology, Weill Cornell Medical College, New York, NY, USA.

[5]Transplantation & Clinical Virology, Department of Biomedicine, University of Basel, Basel, Switzerland.

SUPPLEMENTAL MATERIALS

Table of Contents

	Page
**Supplemental Methods and Results**	**6 - 44**
Urine collection, RNA isolation, and sequencing----------------------------	6
Timing of urine collection relative to kidney allograft biopsy--------------	6 - 8
Quality control--	8
Timing of plasma collection for BK viral load measurement relative to kidney allograft biopsy--	8 - 10
Kidney allograft biopsies--	10
Differential gene expression analysis--	10 - 11
PCA plots and t-SNE visualization of urinary cell transcriptomes across diagnostic categories--	11 - 13
Assessment of Batch Effect--	13 - 15
Biological replicability--	15 - 17
Biological replicability of BKVN-N samples------------------------------------	17
Customized RT-qPCR assays---	17 - 18
Statistics--	19
Study approval---	20

Host cell genes involved in replication-- 20

Host cell genes involved in cell cycle progression--------------------------- 21

Host cell genes involved in DNA replication---------------------------------- 21

Host cell genes involved in repair pathways---------------------------------- 21 - 22

Host cell genes involved in microtubule dynamics-------------------------- 22 - 24

Mitochondrial gene expression--- 24

Antigen presentation in BKVN--- 25

Pattern recognition receptors (PRRs) in BKVN------------------------------ 25

Proinflammatory cytokines and chemokines in BKVN---------------------- 26

T Cell signaling in BKVN--- 27

Expression of T Cell exhaustion and anergy genes in BKVN------------ 27 - 28

CD4 T Cells in BKVN-- 28 - 29

CD4 T cell subset signatures in BKVN-- 29 - 30

CD8 memory and effector T cell signatures in BKVN---------------------- 30 - 31

B cell signaling in BKVN-- 31

Gene Set Enrichment Analysis (GSEA) of KEGG and

Reactome Pathways--- 32

GSEA of KEGG and Reactome Pathways uniquely enriched in

BKVN-P and BKVN-N relative to NR transcriptomes----------------------- 32 - 33

GSEA of KEGG and Reactome pathways uniquely enriched in

BKVN-P samples relative to TCMR transcriptomes------------------------ 34 - 35

GSEA of KEGG and Reactome pathways uniquely enriched in

BKVN-N samples relative to TCMR transcriptomes------------------------ 35 - 37

Noninvasive biomarker for discriminating BKVN from TCMR------------- 37 - 41

Receiver Operating Characteristic curves (ROC) for
discriminating BKVN from TCMR-- 42 - 43

Transcriptomic signature discriminating TCMR from BKVN-------------- 43 - 44

Supplemental References **45 - 51**

Supplemental References--- 45 - 51

Supplemental Figures (N=4) **52 - 57**

Supplemental Figure 1. PCA and t-SNE Visualization of Urinary
Cell transcriptomes Across Diagnostic Categories-------------------------- 52 - 53

Supplemental Figure 2. MDS Plot of Urinary RNA-Seq Profiles Based
on Leading Log-fold Changes-- 54

Supplemental Figure 3. MDS Plot of Batch Sample Distribution
Using Weighted TMM-Limma Normalized Counts with K-function for
Spatial Point Pattern Assessment-- 55 - 56

Supplemental Figure 4. Agreement of Urinary RNA-Seq Counts Between Pre-and Post-biopsy Samples in a Patient with BKVN-P Biopsy: (A) Pearson Correlation; (B) Bland-Altman Plot--------------------- 57

Supplemental Tables (n=44)

Excel file

Supplemental Methods and Results

Urine collection, RNA isolation, and sequencing. A total of 58 urine samples, matched to 57 kidney allograft biopsies from 53 patients, were subjected to RNA sequencing. These samples, selected from our biorepository, represent three major diagnostic categories: Banff Category 1, normal biopsy or nonspecific changes (NR); Banff Category 4, acute T -cell mediated rejection (TCMR); or BK polyomavirus nephropathy (BKVN). Among the 58 urine samples selected, 26 NR biopsy-matched urine samples were from 24 patients, 21 TCMR biopsy-matched urine samples were from 19 patients, and 11 urine samples matched to 10 BKVN biopsies were from 10 patients. Among the 10 BKVN biopsies, 4 showed minimal intragraft inflammation (BKVN-N), and the remaining six displayed intragraft inflammation that met the Banff criteria for TCMR (BKVN-P).

Supplemental Table 1A is a summary of demographics and clinical characteristics of the study cohort. Supplemental Table 1B is a summary of biopsy findings, and Supplemental Table 1C shows the characteristics of the total RNA isolated from the urine samples matched to kidney allograft biopsies.

Timing of urine collection relative to kidney allograft biopsy. Approximately 50 mL of urine was collected from each patient. RNA-Seq cohort (n=58 urine samples matched to 57 biopsies): 55 of the 58 (94.8%) urine samples were collected on the day of the biopsy. BKVN-N group (n=4): All 4 urine samples collected on the day of biopsy. BKVN-P group (n=7): 1 sample was collected 8 days before the biopsy, 5 collected on the biopsy-day, prior to the biopsy, and 1 within 6 hours after the biopsy. TCMR group

(n= 21): Twenty urine samples collected on the day of biopsy; 1 sample within 24 hours after biopsy. No patient received anti-rejection therapy prior to urine collection. No Rejection group (n=26): 25 urine samples collected on the day of the biopsy; 1 sample collected 7 days after the biopsy.

Supplemental Table 43A indicates the timing of urine collection in the RNA-Seq cohort relative to kidney allograft biopsy and shows that all BKVN patients were positive for intragraft SV40 immunostaining, whereas all 21 TCMR biopsies and 26 NR biopsies were negative for intragraft SV40 staining.

External validation cohort (n=230 urine samples matched to 230 biopsies): 208 of 230 (90.4%) urine samples were collected on the day of biopsy. BKVN-N (n=10): Seven urine samples were collected on the day of biopsy; 2 samples 1 day before biopsy, and 1 sample 2 days before biopsy. BKVN-P (n=29): 25 urine samples collected on the day of biopsy; 1 sample 1 day before biopsy, 1 sample 6 days before biopsy, 1 sample 1 day after biopsy and 1 sample 2 days after biopsy. TCMR group (n=46): 40 urine samples collected on the day of TCMR biopsy; 4 samples 1 day after biopsy, and two samples 3 days after the biopsy. NR group (n=145): 136 samples collected on the day of biopsy; 8 samples 1 day after and one sample 2 days after biopsy.

Supplemental Table 43B indicates the timing of urine collection relative to kidney allograft biopsy and shows that all BKVN patients were positive for intragraft SV40 immunostaining, whereas all 46 TCMR biopsies and 145 NR biopsies were negative for intragraft SV40 staining.

Urine samples were centrifuged at 1250x g for 30 minutes at room temperature, and total RNA was isolated from urinary cells using the RNeasy mini kit (Qiagen) followed

by DNase I (Qiagen) treatment. RNA quantity and purity were assessed using the NanoDrop ND-1000 spectrophotometer (Thermo Fisher Scientific), and RNA integrity was evaluated with Agilent 2100 Bioanalyzer (Agilent Technologies Inc.). The quantity, purity, and RNA integrity number (RIN) for the total RNA, isolated from biopsy-matched urine samples, are provided in Supplemental Table 1C.

RNA library preparation and sequencing were performed by the Genomics Core Laboratory at Weill Cornell Medicine. Ribosomal RNA (rRNA) was depleted using the Illumina Ribo Zero Gold human/mouse/rat kit(1). Using Illumina TruSeq Sample Library Preparation v2 kit, mRNA was fragmented into small pieces using divalent cations under elevated temperature. First-strand cDNA synthesis was performed using reverse transcriptase and random primers, followed by second-strand synthesis with DNA Polymerase I and RNAse H. The resulting cDNA fragments underwent end repair, A-tailing, adapter ligation, and PCR enrichment to generate the final libraries. Normalized libraries were pooled and sequenced on the Illumina HiSeq4000platform using paired-end 100-cycle reads.

Quality control. Urine samples were included for downstream analysis if they met two minimum quality thresholds: 20% read alignment to the human GRCh38/hg38 Reference genome and a RIN score of 2 (1).

Timing of plasma collection for BK viral load measurement relative to kidney allograft biopsy. Plasma was collected for clinical indications, and BK viral load was measured using quantitative PCR assays in CLIA-certified laboratories- either

commercial (Quest Diagnostics or Roche Diagnostics) or the clinical laboratory at NewYork-Presbyterian Hospital, the teaching hospital Weill Cornell Medicine. Because assay platforms differ among laboratories, plasma viral loads are not comparable across sites. Nevertheless, in all cases with intragraft SV40 positivity, plasma BK viral load exceeded 10,000 copies per mL, meeting or surpassing the threshold considered diagnostic for BKVN. RNA-Seq cohort (n=10 BKVN biopsies). BKVN-N (n=4): Plasma collected 2 days before biopsy (1 patient), on the day of biopsy (1 patient), 7 days after the biopsy (1 patient) and 22 days after biopsy (1 patient). BKVN-P (n=6): Plasma collected 7 days before biopsy (1 patient), 5 days before biopsy (1 patient), 2 days before biopsy (1 patient), on the day of biopsy (2 patients), and 1 day after biopsy (1 patient).

Supplemental Table 44A provides timing of plasma collection relative to the timing of biopsy, BK viral load values, PCR assay site, and confirms that all BKVN biopsies were positive for intragraft SV40 immunostaining, whereas all 26 NR biopsies and all 21 TCMR biopsies were negative for SV40.

External validation cohort (n=230 biopsies: 39 BKVN biopsies, 46 TCMR biopsies and 145 No Rejection biopsies). BKVN-N (n=10): Plasma collected 13 days before biopsy (1 patient), 4 days before (1 patient), 3 days before (1 patient), 2 days before (1 patient), 1 day before (2 patients), on the day of before (3 patients), and 15 days after biopsy (1 patient). BKVN-P (n=29): Plasma for BKV viral load was collected ranging from -34 days to plus 28 days relative BKVN-P biopsies. Supplemental Table 44B provides timing of plasma collection relative to the timing of biopsy, BK viral load values, PCR assay site, and confirms that all BKVN biopsies were positive for intragraft SV40

immunostaining. All 145 NR biopsies and all 46 TCMR biopsies were negative for SV40 and plasma BKV viral load was not measured in these patients with TCMR biopsies or NR biopsies.

Kidney allograft biopsies. Biopsies were performed under ultrasound guidance and independently evaluated by a senior renal pathologist at our center, based on the Banff 2019 update (2) of the Banff 1997 (3) classification of allograft pathology. Tissue sections were stained with hematoxylin and eosin (H&E), periodic acid–Schiff (PAS), and Masson's trichrome. Additional staining was performed for Simian virus 40 (SV40)/BKV large T antigen and C4d to assess viral infection and complement activation, respectively. Mouse monoclonal IgG2a antibody PAb416 (Abcam, Waltham, MA) was used to detect intragraft BK polyomavirus. This mAb reacts with the SV40 large T antigen and the BK polyomavirus large T antigen because of 75% sharing of amino acid sequence between SV40 and BK polyomavirus large T antigen (4).

Differential gene expression analysis. Sequenced reads were aligned to the human reference genome (GRCh38/hg38) using the STAR aligner (Version 2.4.2) (5). Gene-level quantification was performed using CuffLinks (v 2.2.1) (6) and HTSeq for raw counts (7), to obtain FPKM, and FPKM values were converted to TPM. Genes with counts per million (CPM) greater than 1 in at least two samples were retained for downstream analysis.

Differential gene expression analysis was conducted using the limma-voom package in R (8, 9). which applies weighted linear modeling and empirical Bayes moderation to

estimate log-fold changes (FC) and standard errors. Genes with an FDR < 0.05 were considered significantly differentially expressed and included in supervised analysis.

Functional annotation of DEGs was conducted using clusterProfiler (10), ReactomePA (11), and ENRICHR (12) together with the KEGG 2016 Human database (13) and Reactome database (14), with pathways considered significantly enriched at FDR <0.05.

GSEA (15) and pathway analysis were performed using GSEA software from the clusterProfiler (16) to compare urinary cell gene expression profiles across diagnostic categories and to identify enriched biological functions. Genes were ranked by the t-statistic values, and the pre-ranked GSEA tool was used to identify significantly enriched pathways. The enrichment score (ES) reflects the degree to which a gene set is overrepresented at the extremes of the ranked list, calculated by a running-sum statistic that increases when a gene is in the set and decreases when it is not. The ES represents maximum deviation from zero, indicating whether a predetermined gene is significantly enriched among upregulated or downregulated genes (15).

PCA plots and t-SNE visualization of urinary cell transcriptomes across diagnostic categories. Principal Component Analysis (PCA) was performed to visualize global transcriptomic variation across the four biopsy categories-NR, TCMR, BKVN-N and BKVN-P. The PCA plot, complemented by 95% confidence ellipses for each diagnostic category, illustrates clustering patterns that reflect underlying biological differences. PCA is advantageous for reducing high-dimensional RNA-Seq data into a few principal components, facilitating interpretation of major sources of variance.

However, PCA assumes linear relationships, and may not fully capture complex, nonlinear structures in transcriptomic data. To address this limitation, we applied t-distributed Stochastic Neighbor Embedding (t-SNE), a nonlinear dimensionality reduction technique that preserves local neighborhood relationships and provides a complementary view of sample segregation. The t-SNE plot facilitates visualization of complex RNA-Seq gene expression data by mapping similarities between samples to spatial proximity in low-dimensional scatter plots. Each point in the t-SNE plot represents a sample; points positioned close together indicate similar gene expression profiles, whereas distant points reflect greater dissimilarity. Distinct clusters in the t-SNE plot typically correspond to biologically relevant groups or diagnostic categories. Together, PCA and t-SNA analyses offer a robust framework for exploring both global and local transcriptomic patterns among the diagnostic categories.

Supplemental Figure 1A-C presents the PCA plots derived from a comprehensive analysis of 17,444 genes analyzed across the 4 diagnostic categories—NR, BKVN-N, BKVN-P, or TCMR. Panel A illustrates Principal Component 1 (PC1), which accounts for 25% of the total variance, alongside Principal Component 2 (PC2), which explains 18% of the variance. PC1 25% variance is a substantial amount, suggesting that significant differences in gene expression patterns among the diagnostic categories. PC2 18% variance further contributes to understanding of data structure. Combined, PC1 and PC2 explain 43% of the variance, allowing for a meaningful interpretation of the gene expression profiles, and the relatively high percentage of variance explained by the first two components suggest there are indeed significant differences in gene expression patterns among the four diagnostic categories. The distribution of data

points highlights the underlying structure and patterns within the gene expression profiles. Panel B mirrors the representation in Panel A, with the enhancement of ellipses indicating multivariate t- distributions. The ellipses encompass a 95% confidence region for each diagnostic category, providing a visual representation of the variability and clustering of the gene expression data by different diagnostic conditions. Panel C (within Panel B), the axes of the four ellipses are displayed, delineating the distinct groups by condition. This representation allows for a clearer understanding of how each diagnostic condition is positioned within the principal component space, signifying the relationship and differences among the diagnostic categories.

Supplemental Figure 1, Panel D displays t-SNE analysis using a perplexity of 4; theta 0.5, and a single output dimension to represent the 4 diagnostic categories (color coded): TCMR, acute T cell mediated rejection; BKVN-P, BK polyomavirus-associated nephropathy with intragraft inflammation; BKVN-N, BK polyomavirus-associated nephropathy without intragraft inflammation; NR, no histological features of acute or chronic rejection. High-dimensional RNA expression data were projected onto dimension 1, with each sample represented as a point along a single axis. Samples that are close together in the original high-dimension space cluster tightly along the line, preserving local structure and reflecting similarities among the conditions.

Assessment of Batch Effect. RNA sequencing performed across multiple batches can introduce technical variability, potentially mask true biological signals or generate

spurious results. Rigorous evaluation and, where warranted, correction for batch effects is essential to ensure the integrity of RNA-Seq analysis; however, excessive correction may also artificially diminish genuine biological differences and distort sample relationships. Thus, careful assessment of batch effects-and their potential impact on data interpretation is fundamental.

In this study, 58 samples were processed in three sequencing batches: Batch 1 (black, n=11) comprised of 3 urine samples matched to 3 NR biopsies, 1 to a BKVN-N biopsy, 3 to 3 BKVN-P biopsies, and 4 to 4 TCMR biopsies; Batch 2 (red, n=20) included 12 urine samples matched to 12 NR biopsies, 1 to a BKVN-N biopsy, 2 to 2 BKVN-P biopsies, and 5 to 5 TCMR biopsies; and Batch 3, (green, n=27) contained 11 urine samples matched to 11 NR biopsies, 12 to 12 TCMR biopsies, 2 to 2 BKVN-N biopsies and 2 urine samples matched to 1 BKVN-P biopsy. Importantly, no batch was uniquely associated with any diagnostic category, minimizing risk of confounding between technical batches and biological conditions (see Supplemental Table 41 for batch-by-condition distribution).

To detect potential batch effects, we generated multidimensional scaling (MDS) plot of urinary RNA-sequencing profiles based on leading log-fold (logFC) distances (Supplemental Figure 2)**.** Sample batches were visualized in MDS space using weighted TMM limma normalized counts. Each point in the plot represented one urine sample matched to a kidney allograft biopsy, with colors indicating batch assignment and independent of 4 diagnostic categories, NR, TCMR, BKVN-N and BKVN-P. Distances between points reflected differences in global gene expression profiles, with samples closer together are more similar. The first dimension explained 23% of leading

logFC variance, and the second dimension explained 18%. Points did not cluster by batch, supporting minimal or no observable batch effect.

Visual inspection of Supplemental Figure 2 revealed that samples were generally distributed at random within the MDS space, with no pronounced clustering by batch, except for the suggestion of partial clustering for batch 2 (red) among lcpm values.

We performed a formal statistical assessment using spatial K-function analysis. The K-function quantifies the expected number of neighboring points with a radius r, normalizing for overall density, to statistically evaluate the spatial distribution of batch labels in MDS space (17). In Supplemental Figure 3, each batch's sample distribution is depicted, along with K-function curves: Kest (observed, corrected for edge effects), Kpois (r) (theoretical, assuming complete spatial randomness under a homogenous Poisson distribution process), and a 95% simulation-based confidence band under Complete Spatial Randomness (CSR). If the observed curve remains within the confidence interval, batch distribution is consistent with randomness; curves above suggest clustering, while curves below suggest dispersion.

Collectively, these analyses (Supplemental Figures 2 and 3) indicate that meaningful batch effects are unlikely to compromise the biological interpretation of our RNA -Seq data.

Biological replicability. Biological replicability is a cornerstone of RNA-Seq gene expression studies and refers to the consistency of expression profiles observed across independently collected biological samples of the same condition. High replicability

ensures that the measured transcriptomic differences reflect true biological phenomenon rather than technical noise or artifacts.

In this study, biological replicability was assessed under two experimental conditions. First, a urine sample collected within 4 hours prior to the BKVN-P biopsy was compared with the urine sample collected within 6 hours post-BKVN-P biopsy. Second, all 4 urine samples matched to BKVN-N biopsies were analyzed for inter-sample correlation to confirm biological reproducibility. This was considered necessary since the sample size was small, and biological reproducibility among the limited sample size would increase confidence in the observed biopsy-specific transcriptional differences. Pearson correlation coefficients were calculated for each comparison to quantify the linear agreement between gene expression values across replicates. Values above correlation coefficient (r) 0.90 are considered indicative of robust biological replicability, supporting the reliability of the data.

Supplemental Figure 4, Panel A demonstrates the correlation between RNA-Seq count measurements of 17,765 genes in paired urine sample collected prior to the BKVN-P biopsy and the urine sample collected within 6 hours following the biopsy procedure. This analysis yielded Pierson correlation of r=0.964, which reflects exceptionally strong replicability between the pre-and post-biopsy samples.

To complement correlation analysis, and further assess agreement, a Bland-Altman Plot was generated (18). In this plot, each data point represents the difference in RNA-Seq counts between paired samples (Y-axis), versus their average (X-axis); the horizontal lines indicate the mean difference (bias) and the limits of agreement, typically

defined as the mean and plus or minus 1.96 standard deviations. This technique reveals systemic bias, random error, and helps identify potential outliers.

Supplemental Figure 4, Panel B shows the Bland-Altman plot, confirming that almost all RNA-Seq count differences of 17,563 genes fall within the limits of agreement, with minimal bias. This provides clear evidence for excellent agreement between pre-and post-biopsy urine samples from the same individual patient with BKVN-P biopsy and supports reproducibility of RNA-Seq measurements.

Biological replicability of BKVN-N samples. A minimum 3 biological replicates per condition/diagnostic category is generally required for RNA-Seq measurements (19, 20). When sample sizes are small, stringent statistical methods and careful methodological choices are essential to mitigate sample size related limitations. According to the ENCODE consortium guidelines, Pearson correlation coefficients between biological replicates in RNA-Seq experiments exceeding 0.9 is preferable (19).

In this study, the four BKVN-N samples were analyzed. To assess both technical reproducibility and biological similarity, we calculated the inter-sample Pearson correlation coefficient among these 4 urine samples matched to BKVN-N biopsy. As shown in Supplemental Table 42, the mean Pearson correlation coefficient value was 0.9257, surpassing the ENCODE benchmark of 0.90. These findings confirm both technical reproducibility and biological consistency of the data.

Customized RT-qPCR assays. Total RNA (1.0 µg of total RNA in 100 µL) was reverse transcribed to cDNA using TaqMan Reverse Transcription Reagents (Applied

Biosystems). Gene-specific oligonucleotide primers and fluorogenic probes were designed using Primer Express software to quantify target mRNAs and the reference gene 18S rRNA (21). Probes were labeled with 6-carboxy-fluorescein (FAM) at the 5' end and either 6-carboxy-tetramethylrodamine (TAMRA) or a minor groove binder (MGB), dihydrocyclopyrroloindole tripeptide, at the 3' end. FAM functioned as the reporter dye, and TAMRA or MGB as the quencher.

We designed and amplified the BKV VP1 mRNA with the following primers and probe (BKV GenBank accession no. J02038): BKV sense primer 5’-TGCTGATATTTGTGGCCTGTTTACTA-3’ [2355-2380] and anti-sense primer 5’-CTCAGGCGGATCTTAAAATATCTTG- 3’ [2438–2414]. The amplified BKV PCR product was detected in the customized RT-qPCR assay using the TaqMan Probe, 5’-FAM AGCTCTGGAACACAACAGTGGAGAGGCC TAMRA- 3’ [2383-2410], as previously described (22).

A two-step RT-qPCR assay was performed to measure absolute levels of CXCL10 mRNA and CD3E mRNA, using a preamplification step followed by quantification on the Applied Biosystems Quant Studio 6 Flex Real-Time PCR System (Thermo Fisher Scientific) (22).

The standard curve copy numbers in our customized RT-qPCR assays ranged from 25–2.5 million copies (22). RNA adequacy was defined as 18S rRNA $\geq 5.0 \times 10^7$ copies/μg of total RNA and TGF-B1 mRNA $\geq 1.0 \times 10^2$ copies/μg of total RNA, as described.

Statistics. Differential expression analysis of RNA-Seq data across diagnostic categories was performed using the limma package in R (http://bioconductor.org/packages/release/bioc/html/limma.html), applied to voom-normalized raw counts. P-values were adjusted for multiple comparisons using the Benjamini-Hochberg method to control the FDR.

To assess distribution shifts in gene expression, the Kolmogorov-Smirnov (K-S) test was used to compare the cumulative distribution functions (CDFs) of expression values across diagnostic groups or predefined gene sets. The test calculated the maximum vertical distance between CDF curves and determined statistical significance using P values.

A comprehensive assessment of gene-set level changes was performed using both ROAST (Rotation Gene Testing) and CAMERA (Correlation Adjusted Rank gene set test), implemented in the limma R package. ROAST, a self-contained test, evaluated whether predefined gene sets exhibited coordinated expression changes across directionality (upregulation, downregulation, or mixed pattern) (23). CAMERA, a competitive test, assessed whether a predefined gene set was more differently expressed than genes outside the set, while accounting for inter-gene correlation (24). Together, these complementary approaches provided robust insights into pathway-level transcriptional changes.

Receiver operating characteristic (ROC) curve analysis was performed to evaluate the diagnostic performance of the CXCL10 mRNA/CD3E mRNA ratio. The ROC curve plotted true positive vs. false positive rates across thresholds, and the area under the ROC (AUROC) quantified classification accuracy. The Youden index

(Sensitivity+Specificity-1) was used to identify the optimal threshold to distinguish BKVN from TCMR. Comparisons of mRNA abundance across diagnostic groups were performed using the Kruskal-Wallis test for overall group differences. Pairwise comparisons were conducted using the Mann-Whitney U test, and multiple group comparisons were evaluated using Dunn's test for post-hoc analysis. P <0.05 was considered statistically significant.

Study approval. Kidney transplant recipients reported herein provided written informed consent to participate in the study, and the informed consent was obtained before their inclusion in the study. The IRB at Weill Cornell Medicine approved the study. The clinical and research activities that we report here are consistent with the principles of the "Declaration of Istanbul on Organ Trafficking and Transplant Tourism" (25) and the "World Medical Association Declaration of Helsinki on Ethical Principles for Medical Research Involving Human Subjects" (26).

Host cell genes involved in replication. Of the 22 SV40-related genes (27, 28), 14 genes were significantly overexpressed in BKVN-N, 5 in BKVN-P, and 4 in TCMR compared to NR samples (Supplemental Table 6A, Panel A). These in vivo findings are consistent with prior in vitro data showing SV-40 induced gene upregulation of these genes in kidney epithelial cells (28, 29). Pairwise comparison of fold-change revealed significantly higher expression in BKVN-N compared to BKVN-P (P=0.0008) and TCMR (P<0.0001) with no significant difference observed between BKVN-P and TCMR (Supplemental Table 6A, Panel B).

Host cell genes involved in cell cycle progression. BK polyomavirus hijacks host cell cycle pathways to enhance its replication (30). Genes promoting cell cycle progression were significantly upregulated in both BKVN-N and BKVN-P compared to NR and were elevated in TCMR samples also (Supplemental Table 6B, Panel A). In BKVN-N, key inhibitors - RBL2, CDKN1C, CDKN2A, CDKN2B- were down regulated consistent with the loss of p53-mediated G1 arrest. This pattern aligns with the known effects of viral large T-antigen on pRB and TP53 (29, 31). Additional downregulation of WEE1 and CDC14B was observed in BKVN-P. Pairwise comparisons showed significantly higher fold change in BKVN-N compared with BKVN-P (P<0.0004) and with TCMR (P<0.0001), whereas no significant difference was observed between BKVN-P and TCMR (Supplemental Table 6B, Panel B).

Host cell genes involved in DNA replication. Cell cycle gene activation during viral infection is tightly linked to dysregulation of host DNA replication (32). Compared to NR, 11 of 12 DNA replication genes were upregulated in BKVN-N, 5 were upregulated in BKVN-P, and only one in TCMR (Supplemental Table 6C, Panel A). PPP2CA was uniquely under expressed in BKVN-N compared to NR (P=3.49E-02).

Fold change analysis showed significantly higher in BKVN-N compared to BKVN-P (P=0.0453) and with TCMR (P<0.001). In addition, BKVN-P showed a higher fold change than TCMR (P=0.0397) (Supplemental Table 6C, Panel B).

Host cell genes involved in repair pathways. Both BKVN groups showed increased expression of homologous-recombination repair genes, reflecting

persistent DNA damage from viral replication and oxidative stress (33) (Supplemental Table 6D, Panel A). Overexpression of homologous-recombination repair genes was also observed in TCMR samples.

Fold change analysis demonstrated higher expression in BKVN-N compared with BKVN-P (P=0.0093) and with TCMR (P<0.0001). BKVN-P showed higher fold change than TCMR (P=0.0056) (Supplemental Table 6D, Panel B).

Conversely, POLR1D, POLR2M, and CULB were significantly downregulated in BKVN-N, suggesting transcriptional shutdown, viral manipulation of host transcriptional machinery, and tubular epithelial cell injury or dedifferentiation (Supplemental Table 6D, Panel C). In BKVN-P, reduced ERCC2, POLR2M, and EME2 expression points to impaired DNA repair and transcriptional fidelity, and potential cell cycle arrest or apoptosis (Supplemental Table 6D, Panel C). ERCC2 was the only gene under expressed in TCMR. However, fold change differences among BKVN-N, BKVN-P and TCMR samples were not different (Supplemental Table 6D, Panel D).

Host cell genes involved in microtubule dynamics. In BKVN-N, upregulated mitotic kinesins (KIFC1, KIF18B, KIF15, KIF4A, KIF11, KIF22) together with gamma-tubulin complex genes (TUBGCP3/6), TUBE1, TUBA1B, TUBB) (Supplemental Table 6E, Panel A) indicated a proliferative epithelial program with reinforced centrosomes and mitotic spindles while down regulated RAB21, RAB11A, RAB11FIP5, HOOK3 and TRUBB6 (Supplemental Table 6E, Panel B) suggested attenuation of RAB11-medited apical recycling and long-range endosomal transport. This shift redirects microtubule

usage away from polarity maintenance toward spindle assembly and perinuclear viral trafficking (34). Overall, BKVN-N appeared dominated by virally infected, rapidly cycling tubular epithelial cells that repurpose microtubules and motor proteins to facilitate efficient BK polyomavirus entry and nuclear trafficking at the expense of epithelial polarity and receptor recycling.

In BKVN-P, unique overexpression of DNAH1—axonemal dynein heavy chain—indicated activation of a motile cilia/axonemal transcriptional module in injured proximal tubules (Supplemental Table 6E, Panel A), whereas downregulation of multiple cytoplasmic dynein-dynactin adaptors (ACTR1A, BICD2, DCTN5, DYNLL2, DNAL1, KLC3) and Rab11 axis genes (RAB11A, RAB11FIP3/5, KIF1C, KIF21A, TUBG2) (Supplemental Table 6E, Panel B) suggested weakened cytoplasmic dynein cargo transport and impaired RAB11-dependent apical recycling. This pattern was consistent with dedifferentiated or regenerating tubular cells following severe injury and loss of epithelial polarity, and reengagement of repair pathways.

In TCMR, upregulation of KIFC2, KIFC3, KIF1C, HOOK1, and dynamic tubulin isotypes (TUBA1C, TUBB, TUBB6) (Supplemental Table 6. Panels A and B) suggested highly reconfigurable microtubules optimized for vesicle transport, cell migration, and immune synapse formation rather than ciliary or mitotic structures Concurrent downregulation of DNAH1, other axonemal/dynein components, and RAB11A, RAB11FIP5, KIF21A and DYNC1LI1, indicated loss of ciliary programs and weakened RAB11 driven epithelial polarity. Collectively, TCMR was characterized by immune cell-centric cytoskeletal remodeling: dynamic microtubules supporting T cell motility, granule secretion, and tubulitis, accompanied by generalized loss of tubular

epithelial polarity transcripts typical of acute injury.

Across all three diagnostic categories (BKVN-N, BKVN-P and TCMR), compared with NR, suppression of RAB11-dependent apical recycling was a shared feature; however, the dominant microtubule motor programs differed markedly - mitotic motors in BKVN-N, axonemal/ciliary motors in BKVN-P, and immune cell motility/vesicle transport motors in TCMR.

Supplemental Table 6E, Panel C shows that fold change is significantly higher in BKVN-N compared to BKVN-P (P=0.0017) and with TCMR (P<0.0001). Supplemental Table 6E, Panel D shows that fold change is significantly higher in TCMR than in BKVN-P.

***Mitochondrial gene expression**.* BK polyomavirus may exploit mitochondrial pathways to support replication and immune evasion (35). Viral replication disrupts mitochondrial networks, leading to a stress signature marked by discordant regulation of mitochondria-and nucleus-encoded genes in renal tubular epithelial cells (29).

Differential expression analysis of 13 mitochondrial protein-coding genes and 2 rRNA genes (Supplemental Table 6F, Panel A) showed significant overexpression in urine samples matched to BKVN-N biopsies compared to BKVN-P (Dunn's P<0.0001) and TCMR (P<0.001). No significant difference between BKVN-P and TCMR samples was observed (Supplemental Table 6F, Panel B).

These findings support the presence of a BK polyomavirus -associated mitochondrial transcriptome stress pattern.

Antigen presentation in BKVN. HLA class II genes, key to CD4 T cell activation (36). were significantly upregulated in both BKVN-P and TCMR, indicating active antigen presentation (Supplemental Table 7). HLA-DOA gene was uniquely overexpressed in BKVN-P versus NR, suggesting a role in viral antigen processing. In BKVN-N, only HLA-DMB, which facilitates peptide loading by removing CLIP from MHC class II groove (37), was significantly upregulated compared to NR, indicating a more targeted antigen presentation response.

Pattern recognition receptors (PRRs) in BKVN. PRRs detect viral components and initiate innate immune responses (38, 39). The expression in BKVN and TCMR is detailed in Supplemental Table 8. BKVN-P showed broad upregulation of multiple PRR families—Toll-like receptors (TLRs), RIG-I-like receptors (RLRs), Cytosolic DNA-sensors, C-type lectin receptors (CLRs), and the NOD-like receptor (NLRs), indicating strong immune activation, more extensive than TCMR

In BKVN-N, fewer PRR genes were overexpressed compared to NR, consistent with viral inactivation of the mitochondrial innate signaling. Notably, DHX58 (a cytosolic RNA helicase that plays a regulatory role in the innate immune response to viral infections), MB21D1 (cGAS, a cytosolic DNA sensor in the innate immune system), CD209 (C-type lectin receptor expressed on dendritic cells and a participant in immune modulation) and NOD1 (production of proinflammatory cytokines via RIPK2) were overexpressed (Supplemental Table 8), suggesting a targeted immune response despite minimal inflammation.

Proinflammatory cytokines and chemokines in BKVN**.** The upregulation of HLA-class II and PRRs is expected to drive expression of genes for chemokines and cytokines. This expectation is strongly supported by gene expression data shown in Supplemental Table 9.

Chemokine and chemokine receptors. Multiple chemokine and receptor genes were overexpressed in BKVN-P, indicating active immune cell recruitment (Supplemental Table 9A). Conversely, CCL17 (Treg recruiter) and CXCL14 (antiviral defense and tissue homeostasis) were downregulated in BKVN-P vs. NR, suggesting impaired immune regulation and homeostasis. CXC3CL1 was markedly overexpressed in BKVN-N compared to BKVN-P and TCMR, highlighting indicating a distinct cytotoxic inflammatory profile. These findings reflect heightened inflammation with reduced immune regulation and disrupted homeostasis.

Cytokines and cytokine receptors. Cytokine and receptor genes were elevated in BKVN-P than in BKVN-N (Supplemental Table 9B). IL-18 downregulation in BKVN-N may hinder antiviral responses. Reduced LIFR (leukemia inhibitory factor Receptor) and CTF1 (Cardiotrophin-1) in BKVN-P suggest compromised tissue repair and increased injury.

IFNs and TNF/Receptor superfamily. Genes in these families were predominantly expressed in BKVN-P versus BKVN-N, reinforcing heightened inflammation in BKVN-P (Supplemental Table 9C). Concurrent downregulation of ACVR2, BMPR2, and EGF in BKVN-P indicates a shift away from repair and regulation toward a proinflammatory, injury-prone state.

T Cell signaling in BKVN. T cells play a central role in immune responses (40). Of 115 T cell signaling genes (Supplemental Table 10A), 51 were differentially expressed in BKVN or TCMR or NR (Supplemental Table 10B). In BKVN-N samples, the inhibitory molecules CTLA4 and IL10 mRNA were upregulated, alongside downregulation of CDC42, NCK2, AKT2, PDPK1, KRAS, MAPK3 (ERK1), MAPK8 (JNK1), and BCL10, suggest a suppressed or exhausted T cell phenotype driven by chronic BK polyomavirus stimulation.

BKVN-P showed dysregulated T cell responses, with increased of IFNG, TNF, NFATC1, and PDCD1 and CTLA4, indicating activation and potential exhaustion (41, 42). Downregulation of AKT2, PDPK1, DLG1, and phosphatases points to impaired T cell signaling.

Counter-receptors CD80 and CD86 were significantly upregulated in BKVN-N (FC 4.0, P=3.92E-02; FC 5.28, P=1.33E-02) and BKVN-P (FC 9.48, P=1.60E-05; FC 4.08, P=8.85E-03). CD40 was also upregulated in both BKVN-N (FC 4.23, P=1.76E-03) and BKVN-P (FC 4.04, P=2.16E-04).

TCMR samples showed upregulation of genes associated with T cell activation - CD3D/E/G, CD247, CD4, CD8A/B, CD28, CTLA4, PTPRC, ICOS, and ZAP 70 (Supplemental Table 10C). In contrast, BKVN-N showed increased CTLA4 alone, while BKVN-P showed elevated CTLA4, PDCD1, and CD4, consistent with initial activation followed by exhaustion, potentially facilitating viral persistence.

Expression of T Cell exhaustion and anergy genes in BKVN. T cell exhaustion may underlie BK polyoma virus persistence and progression to BKVN. A 40 gene exhaustion

signature (Supplemental Table 11A, (43-45) was significantly upregulated in BKVN-P (Kolmogorov-Smirnov (K-S) test, D=0.634, P=3.15E-14), BKVN-N (D=0.640, P=1.60E-14), and TCMR (D=0.658, P=2.55E-15) versus NR (Main text, Figure 4A), indicating robust enrichment.

In contrast, the 32-gene anergy signature (46) (Supplemental Table 12A, showed modest downregulation in BKVN-N (K-S test, D=0.264, P=2.41E-02), with non-significant shifts in BKVN-P (D=0.224, P=8.33E-02) and TCMR (D=0.229, P=7.25E-02) (Main text, Figure 4B).

ROAST (Rotation Gene Set Testing) (23) and CAMERA (Correlation Adjusted MEan RAnk Gene Set Test) (24), confirmed strong enrichment of exhaustion genes in BKVN subtypes and TCMR (Supplemental Table 11B), while anergy genes showed limited enrichment in BKVN-N and none in BKVN-P (Supplemental Table 12B). These findings support a model of chronic antigen-driven T cell exhaustion, contributing to immune evasion in BKVN.

CD4 T cells in BKVN. Given CD4 over expression in BKVN-P (Supplemental Table 10C), we examined cytotoxic CD4 T cell-associated genes. Several, including CRTAM, IL12RB1/2, IL15RA. ILRA/B, TNFSF4, CCL8, CCR5 were upregulated in BKVN-P (Supplemental Table 13), indicating enhanced cytolytic and trafficking potential.

Notably, IL4I1 (interleukin-4-inducing 1)—a myeloid-derived immunosuppressive enzyme—was significantly overexpressed in BKVN-N (P=0.0073) and BKVN-P samples (P=0.0043) versus NR (Main text, Figure 5A). IL4I1 may impair cytotoxic CD4 T cell

function via reduced TCR and mTORC1 signaling, potentially aiding viral immune evasion (Main text, Figure 5B).

CD4 T cell subset signatures in BKVN. RNA-Seq analysis has revealed enrichment of Th1, Th2, Th17, and Treg gene signatures in TCMR biopsies versus NR biopsies (40, 47, 48). We investigated these subsets in urine matched to BKVN biopsies.

Th1 signature (36 genes, Supplemental Table 14A, (48)**)**. Significant upregulation in BKVN-N (K-S test, D=0.670, P=2.59E-14), BKVN-P (D=0.384, P=5.31E-05), and TCMR (D=0.603, P=1.08E-11) versus NR (Main text, Figure 6A). BKVN-N showed stronger enrichment than BKVN-P (D=0.361, P=0.0176). ROAST and CAMERA confirmed robust enrichment in BKVN-N and TCMR (*Supplemental Table 14B)*.

Th2 signature (27 genes, Supplemental Table 15A, (48)**).** Upregulated in BKVN-N (K-S test, D=0.558, P=1.14E-07), BKVN-P (D=0.568, P=6.001E-08), and TCMR (D=0.568, P=6.32E-08) versus NR (Main text, Figure 6B). ROAST and CAMERA showed robust enrichment in TCMR and modest enrichment in BKVN subtypes *(Supplemental Table 15B)*.

Th17 signature (22 genes, Supplemental Table 16A, (48)**)**. Significantly enriched in BKVN-P (K-S test, D=0.541, P=5.65E-06), BKVN-N (D=0.361, P=6.66E-03), and TCMR D=0.508, P=2.54E-05) versus NR (Main text, Figure 6C). ROAST and CAMERA confirmed the statistically enriched Th17 signature in BKVN-P (and TCMR) compared to enrichment in BKVN-P and TCMR; BKVN-N showed non-significant enrichment (*Supplemental Table 16B)*.

Treg signature (31 genes, Supplemental Table 17A, (47)**).** Upregulated in BKVN-P (K-S test, D=0.690, P=3.75E-13), BKVN-N (D=0.619, P=1.15E-10) and TCMR (D=0.594, P=7.49E-10) versus NR (Main text, Figure 6D). BKVN-P showed stronger enrichment than TCMR (D=0.355, P=3.96E-02); no significant difference between BKVN-P and BKVN-N.

ROAST and CAMERA confirmed strong enrichment of Treg in BKVN-P, and modest enrichment in BKVN-N (*Supplemental Table 17B)*.

Our investigation of CD4 T cell subsets in urine matched to biopsies identified Th1 signature predominance in BKVN-N, and Treg signature being most enriched in BKVN-P. Altogether, our data suggest distinct CD4 T cell subset dynamics across BKVN subtypes.

CD8 memory and effector T cell signatures in BKVN. We assessed enrichment of CD8 memory (45 genes, Supplemental Table 18A, (49)) and effector (24 genes, Supplemental Table 19A) T cell signatures in urines matched to BKVN and TCMR biopsies versus urines matched to NR biopsies,

CD8 memory signature. Significantly upregulated in BKVN-P (K-S test, D=0.756, P=2.20E-16), BKVN-N (D=0.647, P=2.20E-16), and TCMR (D=0.788, P=2.20E-16) versus NR (Main text, Figure 7 A). Differences between BKVN-P versus BKVN-N (D=0.311, P=0.025) and BKVN-P versus TCMR (D=0.289, P=0.046) were significant. ROAST and CAMERA confirmed the strong enrichment across all groups *Supplemental Table 18B)*.

CD8 effector T cell signature. Upregulated in BKVN-P (K-S test, D=0.934, P=2.20E-16), BKVN-N (D=0.827, P=1.39E-14), and TCMR (D=0.881, P=2.20E-16) versus NR (Main text, Figure 7B). Significant differences were observed between BKVN-P versus BKVN-N (D=0.5417, P=0.001); BKVN-N versus TCMR (D=0.456, P=0.011); BKVN-P versus TCMR was not significant (D=0.167, P=0.902). ROAST and CAMERA confirmed strong enrichment of in BKVN-P and TCMR, but not BKVN-N *(Supplemental Table 19B)*.

B cell signaling in BKVN. Beyond antibody production, B cells may contribute to BKVN via antigen presentation and inflammation (50). Of 79 B cell signaling genes (Supplemental Table 20A), 31 were differentially expressed across BKVN-N, BKVN-P and TCMR versus NR (Supplemental Table 20B).

BKVN-N. Upregulation of inhibitory receptors (LILRB1/4/5, CD72) and down regulation of KRAS, BCL10, and MAPK3 suggest a regulatory B cell phenotype with suppressed B cell signaling.

BKVN-P. Gene expression indicated active cell signaling, with upregulation of BTK, BLK, CD79A/B, NFATC1, JUN, NFKB1, RASGRP3, IFITM1, LILRB1/4) alongside AKT2 downregulation, reflecting activation with regulatory feedback.

TCMR. Enhanced B cell activation and signaling were evident via increased LILRB1, IFITM, CD72, RAC2, PRKCB, PTPN6 [SHP-1], CARD11, FCGR2B, INPP5D (SHIP1), NFATC1/3, PIK3CD, PPP3CC, highlighting a role for B cells in TCMR pathogenesis.

Gene Set Enrichment Analysis (GSEA) of KEGG and Reactome Pathways. To provide functional context and mechanistic insights into the DEGs, we performed GSEA using both KEGG and Reactome pathway databases. GSEA evaluates predefined gene sets involved in specific biological processes, thereby enhancing the interpretability of the DEG lists. While KEGG pathways offer broad biological coverage, Reactome pathways often provide more detailed and curated insights, which can be particularly informative in immunological and signaling contexts.

GSEA of KEGG and Reactome Pathways uniquely enriched in BKVN-P and BKVN-N relative to NR transcriptomes. One key objective was to identify pathways uniquely enriched in the BKVN-P and BKVN-N groups. Among the 2,327 DEGs identified between BKVN-P and NR samples, 1,731 genes were unique to BKVN-P, while 596 genes overlapped with those differentially expressed in BKVN-N. Conversely, of the 1,497 DEGs between BKVN-N and NR samples, 901 genes were unique to BKVN-N (Main text, Venn Diagram in Figure 9)

Figure 9, in main text, presents ridge plot distributions of enriched pathways based on the 1,731 DEGs unique to BKVN-P samples. Panel A displays 22 significantly enriched KEGG pathways (adjusted P <0.05, Supplemental Table 23), while Panel B shows the top 30 of 53 significantly enriched Reactome pathways (adjusted P <0.05, Supplemental Table 24).

The positively enriched biological pathways in BKVN-P samples reflect robust activation of immune responses, cytokine signaling, including interferon-mediated antiviral defense, B cell receptor signaling pathway, antigen presentation and

costimulation, and pro-inflammatory chemokine activity. In contrast, the negatively enriched pathways suggest a loss of metabolic and solute transport functions of epithelial cells, impaired epithelial cell repair mechanisms, and compromised epithelial barrier integrity—likely consequences of viral cytopathic effects.

Figure 9, in main text, also displays ridge plot distributions of enriched pathways based on GSEA of the 901 DEGs unique to BKVN-N samples. Panel C shows one positively enriched cell cycle pathway and 2 negatively enriched - endocytosis and IL-17 signaling- KEGG pathways (adjusted P<0.05, Supplemental Table 25), and Panel D presents the top 30 of 62 significantly enriched Reactome pathways (adjusted P <0.05, Supplemental Table 26). DNA damage response and repair, centrosome and microtubule organization, telomere maintenance, AURKA activation, and Golgi-to-ER retrograde transport features are consistent with BK polyomavirus-induced reprogramming of renal tubular epithelial cells. The negatively enriched pathways reflected suppression of host defense and epithelial cell differentiation, including downregulation of innate immune signaling, attenuated inflammatory responses, tubular injury, and loss of epithelial integrity.

The contrasts in pathway enrichment between BKVN-P and BKVN-N samples, based on the 1,731 and 901 DEGs, respectively, are vividly illustrated through cluster profiling. Figure 10A, in main text, compares GSEA results for KEGG pathways between the two groups, while Figure 10B, in main text, presents a similar comparison for Reactome pathways. These visualizations highlight distinct biological programs activated in each subgroup, underscoring the divergent molecular mechanisms underlying BKVN-P and BKVN-N subtypes.

GSEA of KEGG and Reactome pathways uniquely enriched in BKVN-P samples relative to TCMR transcriptomes. Given that Banff acute lesion scores in BKVN-P samples met diagnostic thresholds for TCMR, and that the histological features of BKVN-P are largely indistinguishable from those of TCMR, we investigated whether GSEA of KEGG and Reactome pathways could provide insights discriminating BKVN-P from TCMR. To this end, we first identified DEGs by comparing transcriptomes of urinary cells from 21 urine samples matched to 21 TCMR biopsies with transcriptomes of urinary cells from 26 urine samples matched to 26 NR biopsies. This analysis yielded 2,654 DEGs in the TCMR group. We then compared these DEGs with the 2,327 DEGs identified by comparing the transcriptomes of BKVN-P samples and NR samples. The comparison of 2,654 DEGs with the 2,327 DEGs revealed 1,599 DEGs unique to TCMR, 1,272 DEGs unique to BKVN-P, and 1,055 shared DEGs (Main text, Venn diagram in Figure 11).

Figure 11, in main text, presents GSEA results highlighting distinct biological pathways enriched in TCMR and BKVN-P urinary cell transcriptomes. Figure 11A presents ridge plot distributions of enriched KEGG pathways based on the 1,599 DEGs unique to TCMR samples (Supplemental Table 27). Figure 11B displays ridge plot distributions of enriched KEGG pathways based on the 1,272 DEGs unique to BKVN-P samples (Supplemental Table 28). Figure 11C compares KEGG pathway enrichment between TCMR and BKVN-P samples. Figure 12A presents ridge plot distributions of enriched Reactome pathways based on the 1,599 DEGs unique to TCMR samples (Supplemental Table 29). Figure 12B presents ridge plot distributions of enriched Reactome pathways based on the 1,272 DEGs unique to BKVN-P samples

(Supplemental Table 30). Figure 12C compares Reactome pathway enrichment between TCMR and BKVN-P samples

Altogether, TCMR samples show robust enrichment of TCR signaling, CD28 co-stimulation, innate immune response, GTPase-mediated signaling cascade, integrin-mediated interactions, immune cell infiltration, and extracellular matrix (ECM) remodeling. Negatively enriched pathways suggest downregulation of homeostatic transport and epithelial barrier maintenance. BKVN-P samples reveal strong immune activation of innate immune responses, chemokine-driven immune cell recruitment, interferon and cytokine signaling characteristic of viral infections, and suppression of epithelial and metabolic homeostasis- likely reflecting inflammation and virus-induced tissue damage.

The comparative analysis showed that while immune system activation is a shared feature of TCMR and BKVN-P, TCR signaling and CD28 co-stimulation are dominant in TCMR and largely absent in BKVN-P. Conversely, BKVN-P samples showed stronger enrichment of innate immune and GPCR-mediated pathways, along with more pronounced downregulation of transport, metabolic, and epithelial barrier functions compared to TCMR samples.

GSEA of KEGG and Reactome pathways uniquely enriched in BKVN-N samples relative to TCMR transcriptomes. We also compared the 2,654 DEGs identified in TCMR samples vs. NR with the 1,497 DEGs from the BKVN-N vs.NR samples. This comparison revealed 568 shared DEGs (Supplemental Table 31), 2,086 DEGs unique

to TCMR (Supplemental Table 32), and 929 DEGs unique to BKVN-N (Supplemental Table 33) (Main text, Venn diagram in Figure 13).

Figure 13A, in main text, presents ridge plot distributions of enriched KEGG pathways based on the 2,086 DEGs unique to TCMR samples (Supplemental Table 34). Figure 13B displays ridge plot distributions of enriched KEGG pathways based on the 929 DEGs unique to BKVN-N samples (Supplemental Table 35). Figure 13C compares KEGG pathway enrichment and between TCMR and BKVN-N samples. Figure 14A presents ridge plot distributions of enriched Reactome pathways based on the 2,086 DEGs unique to TCMR samples (Supplemental Table 36). Figure 14B presents ridge plot distributions of enriched Reactome pathways based on the 929 DEGs unique to BKVN-N samples (Supplemental Table 37). Figure 14C compares Reactome pathway enrichment between TCMR and BKVN-N samples

The positively enriched pathways in TCMR samples reflect again strong T cell activation and proliferation, cytokine signaling (including interferon signaling), innate immune cell recruitment, and inflammation. Negatively enriched pathways indicate suppression of tissue-specific metabolic and structural functions.

The positively enriched pathways in BKVN-N samples are consistent with viral hijacking of host replication machinery, activation of DNA damage response and repair, replication stress, suppression of epithelial cell differentiation and signaling, loss of epithelial barrier integrity, and altered lipid and hormone signaling.

The comparative analysis of TCMR and BKVN-N samples showed that while both groups shared chemokine signaling, TCR signaling, and CD28 co-stimulation were dominant in TCMR and virtually absent in BKVN-N. In contrast, BKVN-N samples

showed stronger enrichment of viral protein interaction pathways, viral-driven host cell proliferation, replication stress, and checkpoint activation and suppression of epithelial differentiation and signaling pathways.

***Noninvasive biomarker for discriminating BKVN from TCMR*.** Our investigation of urinary cell transcriptomes of TCMR, BKVN-P, and BKVN-N samples identified that several chemokine mRNAs were markedly overexpressed in BKVN samples compared to both NR and TCMR samples. Notably, CXCL10 mRNA showed an FC of 23.53 in BKVN-N vs. NR (adjusted P=2.17E-03), FC of 41.96 in BKVN-P vs. NR (adjusted P=5.07E-05), and FC of only 4.59 in TCMR vs. NR (adjusted P=2.39E-02) (Supplemental Table 9A).

Conversely, expression profiles of mRNA for T cell signaling proteins identified that CD3E mRNA is not significantly overexpressed, compared to NR samples, in either BKVN-N (FC, 2.23, adjusted P=5.12E-01) or in BKVN-P (FC,2.61, adjusted P=1.88E-01) and significantly overexpressed in TCMR samples compared to NR samples (4.82, adjusted P=8.00E-03) (Supplemental Table 10C).

The contrasting patterns—T cell dominance in TCMR and chemokine enrichment in BKVN—led us to investigate whether a ratio of CXCL10 mRNA to CD3E mRNA could serve as a discriminatory biomarker between BKVN and TCMR. Accordingly, we measured CXCL10 mRNA and CD3E mRNA using and calculated the ratio of CXCL10 mRNA to CD3E mRNA in the RNA-Seq cohort and the external validation cohort. In addition to RNA-Seq, we used an orthogonal technology, customized RT-qPCR to measure absolute copy numbers of mRNA in urinary cells.

Urinary cell CXCL10 mRNA abundance is diagnostic of TCMR, and the CXCL10 mRNA/CD3E mRNA ratio differentiates BKVN from TCMR. In urine samples matched to kidney allograft biopsies, we quantified CXCL10 mRNA and CD3E mRNA levels, calculated the ratio of CXCL10 mRNA to CD3E mRNA, and assessed the ability of these measures to distinguish diagnostic categories. Specifically, we assessed their performance as biomarkers of TCMR and their utility in differentiating TCMR from BKVN.

Discovery cohort (58 urine samples matched to 57 biopsies), RNA-Seq quantification (Main text, Figure 15A-C). The median (IQR) of CXCL10 transcripts per million (TPM) was 0.8 (2) in the 26 NR biopsy-matched urine samples, 8.8 (30) in 21 TCMR biopsy-matched samples, 50.0 (85) in 4 BKVN-N biopsy-matched urine samples, and 42.0 (69) in 7 BKVN-P urines matched to 6 BKVN-P biopsies (Figure 15A). A Kruskal Wallis [KW] test indicated significant differences among the diagnostic categories (Chi-squared = 26.3, df=3, P <0.0001). Post-hoc Dunn's test results were as follows: NR vs. TCMR, P=0.0069; NR vs. BKVN-N, P=0.009; NR vs. BKVN-P, P<0.0001; TCMR vs. BKVN-N, P=0.5993; TCMR vs. BKVN-P, P=0.0737; and BKVN-N vs. BKVN-P, P=1.

The median (IQR) CD3E TPM was 0.48 (1.75) in NR samples, 2.85 (7.92) in TCMR samples, 1.94 (3.17) in BKVN-N samples, and 3.53 (2.36) in BKVN-P samples (Figure 15B). A Kruskal Wallis test indicated significant differences among the diagnostic categories (Chi-squared = 16.2, df=3, P<0.0001). Post-hoc Dunn's test results were as follows: NR vs. TCMR, P=0.0006; NR vs. BKVN-N, P=0.5591; NR vs. BKVN-P,

P=0.0318; TCMR vs. BKVN-N, P=1; TCMR vs. BKVN-P, P=1; and BKVN-N vs. BKVN-P, P=1.

The median (IQR) CXCL10 TPM/CD3E TPM ratio was 1.0 (2.71) in NR, 0.93 (2.77) in TCMR, 13.78 (9.50) in BKVN-N and 14.30 (22.29) in BKVN-P (Figure 15C). A Kruskal Wallis test indicated significant differences among the diagnostic categories (Chi-squared = 13.57, df=3, P<0.0001). Post-hoc Dunn's test results were as follows: NR vs. TCMR, P=1; NR vs. BKVN-N, P=0.094; NR vs. BKVN-P, P 0.0093; TCMR vs. BKVN-N, P=0.0918; TCMR vs. BKVN-P, P=0.0098; and BKVN-N vs. BKVN-P, P=1.

Discovery cohort (58 urine samples matched to 57 biopsies), customized RT-qPCR quantification (Main text, Figure 15 D-F). We used an orthogonal RT-qPCR assay to measure absolute copy numbers of CXCL10 mRNA and CD3E mRNA in biopsy-matched urine samples. In the RNA-Seq cohort (Discovery Set), the median (IQR) CXCL10 mRNA copy number was 170 (636) per microgram of total RNA in 26 NR samples, 2830 (27,236) in 21 TCMR samples, 13,650 (13,793) in 4 BKVN-N samples, and 32,500 (52,350) in 7 BKVN-P urines matched to 6 BKVN-P biopsies (Figure 15D). A Kruskal Wallis test indicated significant differences among the diagnostic categories (Chi-squared = 22.2, df=3, P <0.0001). Post-hoc Dunn's test results were as follows: NR vs. TCMR, P=0.0102; NR vs. BKVN-N, P=0.0452; NR vs. BKVN-P, P=0.0001; TCMR vs. BKVN-N, P=1; TCMR vs. BKVN-P, P=0.1109; and BKVN-N vs. BKVN-P, P=1.

The median (IQR) CD3E mRNA copy number was 512 (4968) in NR samples, 8450 (50,910) in TCMR samples, 4655 (3884) in BKVN-N samples, and 13,600 (21,620) in BKVN-P samples (Figure 15E). A Kruskal Wallis test indicated significant differences among the diagnostic categories (Chi-squared = 15.3, df=3, P<0.0001). Post-hoc

Dunn's test results were as follows: NR vs. TCMR, P=0.0017; NR vs. BKVN-N, P=1; NR vs. BKVN-P, P=0.0128; TCMR vs. BKVN-N, P=0.982; TCMR vs. BKVN-P, P=1; and BKVN-N vs. BKVN-P, P=0.7169.

The median (IQR) CXCL10 mRNA copies/CD3E mRNA copies ratio was: 0.24 (0.87) in NR samples, 0.18 (0.42) in TCMR samples, 3.79 (2.35) in BKVN-N samples and 1.88 (8.31) in BKVN-P samples (Figure 15F). A Kruskal Wallis test indicated significant differences among the diagnostic categories (Chi-squared = 18.49, df=3, P<0.0001). Post-hoc Dunn's test results were as follows: NR vs. TCMR, P=1; NR vs. BKVN-N, P=0.0224; NR vs. BKVN-P, P=0.0081; TCMR vs. BKVN-N, P=0.0086; TCMR vs. BKVN-P, P=0.0023; and BKVN-N vs. BKVN-P, P=1.

External validation cohort (230 urine samples matched to 230 biopsies), customized RT-qPCR quantification (Main text, Figure 15 G-I). Demographic and clinical details, biopsy findings and total RNA characteristics are summarized in Supplemental Tables 38A-C, respectively; all participants were managed under a standardized immunosuppression protocol.

In the external validation set, the median (IQR) CXCL10 mRNA copy number per microgram of total RNA was 274 (950) in 145 NR biopsy- matched urine samples, 1734 (5492) in 46 TCMR biopsy- matched urine samples, 8785 (17,768) in 10 BKVN-N biopsy- matched urine samples, and 6097 copies (14,961) in 29 BKVN-P biopsy-matched urine samples (Figure 15G). A Kruskal Wallis test indicated significant differences among the diagnostic categories (Chi-squared = 62.8, df=3, P<0.0001). Post-hoc Dunn's test results were as follows: NR vs. TCMR, P=0.0013; NR vs. BKVN-

N, P=0.0001; NR vs. BKVN-P, P<0.0001; TCMR vs. BKVN-N, P=0.0849; TCMR vs. BKVN-P, P=0.002; and BKVN-N vs. BKVN-P, P=1.

The median (IQR) CD3E mRNA copy number was 1594 (5292) in NR samples, 5772 (17,514) in TCMR samples, 2301 (4202) in BKVN-N samples, and 5927 (14,936) in BKVN-P samples (Figure 15H). A Kruskal Wallis test indicated significant differences among the diagnostic categories (Chi-squared = 19.9, df=3, P <0.0001). Post-hoc Dunn's test results were as follows: NR vs. TCMR, P=0.0015; NR vs. BKVN-N, P=1; NR vs. BKVN-P, P=0.0019; TCMR vs. BKVN-N, P=1; TCMR vs. BKVN-P, P=1 and BKVN-N vs. BKVN-P, P=1.

The median (IQR) CXCL10 mRNA to CD3E mRNA ratio was: 0.22 (0.69) in NR samples, 0.23 (0.59) in TCMR samples, 3.49 (3.67) in BKVN-N samples and 1.79 (2.14) in BKVN-P samples (Figure 15I). A Kruskal Wallis test indicated significant differences among the diagnostic categories (Chi-squared = 48.33, df=3, P<0.0001). Post-hoc Dunn's test results were as follows: NR vs. TCMR, P>0.9999; NR vs. BKVN-N, P<0.0001; NR vs. BKVN-P, P<0.0001; TCMR vs. BKVN-N, P<0.0001; TCMR vs. BKVN-P, P<0.0001; and BKVN-N vs. BKVN-P, P=1.

Altogether, our data demonstrate that the expression levels of CXCL10 mRNA (and CD3E mRNA) discriminate TCMR from NR, consistent with earlier reports that CXCL10 mRNA or protein discriminate NR from TCMR (51-53). We identified in the current study that the ratio of CXCL10 mRNA to CD3E mRNA accurately discriminates BKVN-N from TCMR, BKVN-P from TCMR, and BKVN from TCMR.

Receiver Operating Characteristic curves (ROC) for discriminating BKVN from TCMR. We performed comprehensive ROC curve analyses across the discovery set, the external validation set and the combined data set to evaluate the diagnostic performance of the ratio of CXCL10 mRNA to CD3E mRNA for discriminating BKVN-N from TCMR, BKVN-P from TCMR, and BKVN from TCMR.

Figure 16, in main text, illustrates ROC curves for BKVN-N versus TCMR, BKVN-P versus TCMR, and BKVN versus TCMR within each data set. In the discovery set, discrimination between BKVN-N and TCMR achieved an area under the receiver operating characteristic curve (AUROC) of 0.93 (95% Confidence Interval [CI] 0.83-1, P=0.002, Figure 16A), while BKVN-P versus TCMR yielded an AUROC of 0.9 (95% CI, 0.79-1, P=0.0004, Figure 16B), and all BKVN cases versus TCMR an AUROC of 0.91(95% CI, 0.82-1, P=1.96E-05, Figure 16C).

In the external validation set, AUROC values were 0.92 (95% CI, 0.85-1, P=1.42E-06) for BKVN-N versus TCMR (Figure 16D), 0.82 (95% CI, 0.71-0.92, P=7.8E-07) for BKVN-P versus TCMR (Figure 16E), and 0.84 (95% CI, 0.76-0.93, P=4.05E-09) for all BKVN cases versus TCMR (Figure 16F).

In the combined set, the AUROC values remained robust: 0.93 (95% CI, 0.88-0.99, P=2.3E-07) for BKVN-N versus TCMR (Figure 16G), 0.83 (95% CI, 0.74-0.92, P=2.03E-08) for BKVN-P versus TCMR (Figure 16H) and 0.85 (95% CI, 0.78-0.92, P=1.52E-10) for all cases of BKVN versus TCMR (Figure 16I). The optimal cut point, which maximized the combined sensitivity and specificity, was 0.67. At this threshold, the ratio distinguished BKVN from TCMR with a sensitivity of 85% and a specificity of 76%.

Collectively, our findings support the utility of the ratio of CXCL10 mRNA to CD3E

mRNA as a noninvasive biomarker capable of distinguishing BKVN-N (without intragraft inflammation) and BKVN-P (with intragraft inflammation) from TCMR, which is characterized by prominent intragraft inflammation.

Transcriptomic signature discriminating TCMR from BKVN (Main text, Figure 17). To define a transcriptome-wide signature that distinguishes TCMR from BKVN, 21 urinary cell transcriptomes matched to 21 TCMR biopsies were compared with 11 urinary cell transcriptomes matched to 10 BKVN biopsies (4 BKVN-N urine sample matched to 4 BKVN-N biopsies and 7 urine samples matched 6 BKVN-P biopsies). Figure 17A shows a volcano plot summarizing differentially expressed genes (DEGs). This analysis identified 104 DEGs, with 91 significantly overexpressed (adjusted P<0.05) and 13 under expressed (adjusted P<0.05) in BKVN compared with TCMR; these DEGs are listed Supplemental Table 39.

Elastic net regression was then used for robust feature selection by penalizing less informative predictors, yielding a parsimonious gene set with maximal discriminatory power between the two diagnostic categories. The cv.glmnet function identified the optimal lambda by cross-validation, selecting the model with the minimum error of lambda at alpha=0.8. Figure 17B displays the glmnet cross-validation plot, which shows mean-squared and misclassification error across lambda values; vertical lines indicate the optimal penalties (minimum error and more parsimonious 1-SE solution).

Shrinkage of the 104 DEGs identified an 18-gene panel (PEX19, NAP1L4, AKTIP, GATC, OIP5-AS1, PCMTD2, MXD4, UNC119B, RBL2, ZBTB44, SRSF9, DDX6, NR1H3, IQGAP3, KIAA1199, ADPRH, ETV7, and JAKMIP2) that perfectly discriminated

TCMR from BKVN with an accuracy of 100% (95% CI, 0.89-1.0) and an AUROC of 1.0. A bootstrap evaluation of the 18-gene model with 1000 iterations confirmed the high performance, yielding an accuracy of 0.987 (95% CI, 0.984-0.989) and an AUROC of 0.999.

Figure 17C shows the elastic net coefficient plot, which depicts the estimated coefficients for each of the 18 gene predictors, illustrating their direction and relative contribution to the model that separates TCMR from BKVN (AUROC =1.0). Supplemental Table 40 lists the 18 predictor genes and their elastic net coefficients, random forest validation (mean decrease in accuracy and Gini), and TCMR vs. BKVN fold changes for the 18 predictors, and the adjusted P values for the fold changes.

To evaluate model robustness, a bootstrapped random forest was fitted using the selected genes. Using N=1000 bootstrap samples and the caret package train function with the optimal mtry=2, this model achieved similarly high performance, with an accuracy 0.973 (95% CI, 0.970-0.975) and an AUROC of 0.995 (Figure 17D).

Concluding Remarks

In sum, our in-depth transcriptomic analyses reveal profound and divergent reprogramming of host and immune pathways in urinary cells of kidney allograft recipients with BKVN, offering mechanistic insights into viral pathogenesis, immune dysregulation, and potential diagnostic signatures to differentiate BKVN from allograft rejection. These findings set the stage for new molecular approaches to noninvasive diagnosis, risk stratification, and tailored therapy in the care of kidney transplant recipients.

Supplemental References

1. Verma A, Muthukumar T, Yang H, Lubetzky M, Cassidy MF, Lee JR, et al. Urinary cell transcriptomics and acute rejection in human kidney allografts. *JCI Insight.* 2020;5(4).
2. Loupy A, Haas M, Roufosse C, Naesens M, Adam B, Afrouzian M, et al. The Banff 2019 Kidney Meeting Report (I): Updates on and clarification of criteria for T cell- and antibody-mediated rejection. *Am J Transplant.* 2020;20(9):2318–31.
3. Racusen LC, Solez K, Colvin RB, Bonsib SM, Castro MC, Cavallo T, et al. The Banff 97 working classification of renal allograft pathology. *Kidney Int.* 1999;55(2):713–23.
4. Mann RS, and Carroll RB. Cross-reaction of BK virus large T antigen with monoclonal antibodies directed against SV40 large T antigen. *Virology.* 1984;138(2):379–85.
5. Dobin A, Davis CA, Schlesinger F, Drenkow J, Zaleski C, Jha S, et al. STAR: ultrafast universal RNA-seq aligner. *Bioinformatics.* 2013;29(1):15–21.
6. Trapnell C, Williams BA, Pertea G, Mortazavi A, Kwan G, van Baren MJ, et al. Transcript assembly and quantification by RNA-Seq reveals unannotated transcripts and isoform switching during cell differentiation. *Nat Biotechnol.* 2010;28(5):511–5.
7. Anders S, Pyl PT, and Huber W. HTSeq--a Python framework to work with high-throughput sequencing data. *Bioinformatics.* 2015;31(2):166–9.

8. Ritchie ME, Phipson B, Wu D, Hu Y, Law CW, Shi W, et al. limma powers differential expression analyses for RNA-sequencing and microarray studies. *Nucleic Acids Res.* 2015;43(7):e47.

9. Law CW, Chen Y, Shi W, and Smyth GK. voom: Precision weights unlock linear model analysis tools for RNA-seq read counts. *Genome Biol.* 2014;15(2):R29.

10. Xu S, Hu E, Cai Y, Xie Z, Luo X, Zhan L, et al. Using clusterProfiler to characterize multiomics data. *Nat Protoc.* 2024;19(11):3292–320.

11. Yu G, and He QY. ReactomePA: an R/Bioconductor package for reactome pathway analysis and visualization. *Mol Biosyst.* 2016;12(2):477–9.

12. Kuleshov MV, Jones MR, Rouillard AD, Fernandez NF, Duan Q, Wang Z, et al. Enrichr: a comprehensive gene set enrichment analysis web server 2016 update. *Nucleic Acids Res.* 2016;44(W1):W90–7.

13. Kanehisa M, Sato Y, Kawashima M, Furumichi M, and Tanabe M. KEGG as a reference resource for gene and protein annotation. *Nucleic Acids Res.* 2016;44(D1):D457–62.

14. Fabregat A, Sidiropoulos K, Viteri G, Forner O, Marin-Garcia P, Arnau V, et al. Reactome pathway analysis: a high-performance in-memory approach. *BMC Bioinformatics.* 2017;18(1):142.

15. Subramanian A, Tamayo P, Mootha VK, Mukherjee S, Ebert BL, Gillette MA, et al. Gene set enrichment analysis: a knowledge-based approach for interpreting genome-wide expression profiles. *Proc Natl Acad Sci U S A.* 2005;102(43):15545–50.

16. Yu G, Wang LG, Han Y, and He QY. clusterProfiler: an R package for comparing biological themes among gene clusters. *OMICS.* 2012;16(5):284–7.

17. Baddeley A, Rubak E, Turner R, and Spatial Point Patterns: Methodology and Applications with R. Chapman and Hall/CRC Press, London. 2015.

18. Bland JM, and Altman DG. Statistical methods for assessing agreement between two methods of clinical measurement. *Lancet.* 1986;1(8476):307–10.

19. . ENCODE Guidelines and Best Practices for RNA-Seq. https://share.google/XqItbnLrA3wOXVJA1.

20. Schurch NJ, Schofield P, Gierlinski M, Cole C, Sherstnev A, Singh V, et al. How many biological replicates are needed in an RNA-seq experiment and which differential expression tool should you use? *RNA.* 2016;22(6):839–51.

21. Suthanthiran M, Schwartz JE, Ding R, Abecassis M, Dadhania D, Samstein B, et al. Urinary-cell mRNA profile and acute cellular rejection in kidney allografts. *N Engl J Med.* 2013;369(1):20–31.

22. Muthukumar T, Dadhania D, Ding R, Snopkowski C, Naqvi R, Lee JB, et al. Messenger RNA for FOXP3 in the urine of renal-allograft recipients. *N Engl J Med.* 2005;353(22):2342–51.

23. Wu D, Lim E, Vaillant F, Asselin-Labat ML, Visvader JE, and Smyth GK. ROAST: rotation gene set tests for complex microarray experiments. *Bioinformatics.* 2010;26(17):2176–82.

24. Wu D, and Smyth GK. Camera: a competitive gene set test accounting for inter-gene correlation. *Nucleic Acids Res.* 2012;40(17):e133.

25. International Summit on Transplant T, and Organ T. The Declaration of Istanbul on Organ Trafficking and Transplant Tourism. *Clin J Am Soc Nephrol.* 2008;3(5):1227–31.

26. World Medical A. World Medical Association Declaration of Helsinki: ethical principles for medical research involving human subjects. *JAMA.* 2013;310(20):2191–4.

27. Waga S, Bauer G, and Stillman B. Reconstitution of complete SV40 DNA replication with purified replication factors. *J Biol Chem.* 1994;269(14):10923–34.

28. Abend JR, Low JA, and Imperiale MJ. Global effects of BKV infection on gene expression in human primary kidney epithelial cells. *Virology.* 2010;397(1):73–9.

29. Weissbach FH, Follonier OM, Schmid S, Leuzinger K, Schmid M, and Hirsch HH. Single-cell RNA-sequencing of BK polyomavirus replication in primary human renal proximal tubular epithelial cells identifies specific transcriptome signatures and a novel mitochondrial stress pattern. *J Virol.* 2024;98(12):e0138224.

30. Moens U, Calvignac-Spencer S, Lauber C, Ramqvist T, Feltkamp MCW, Daugherty MD, et al. ICTV Virus Taxonomy Profile: Polyomaviridae. *J Gen Virol.* 2017;98(6):1159–60.

31. DeCaprio JA, and Garcea RL. A cornucopia of human polyomaviruses. *Nat Rev Microbiol.* 2013;11(4):264–76.

32. Kelly TJ, and Brown GW. Regulation of chromosome replication. *Annu Rev Biochem.* 2000;69:829–80.

33. Li X, and Heyer WD. Homologous recombination in DNA repair and DNA damage tolerance. *Cell Res.* 2008;18(1):99–113.

34. Seo D, and Gammon DB. Manipulation of Host Microtubule Networks by Viral Microtubule-Associated Proteins. *Viruses.* 2022;14(5).

35. Manzetti J, Weissbach FH, Graf FE, Unterstab G, Wernli M, Hopfer H, et al. BK Polyomavirus Evades Innate Immune Sensing by Disrupting the Mitochondrial Network and Promotes Mitophagy. *iScience.* 2020;23(7):101257.

36. Roche PA, and Furuta K. The ins and outs of MHC class II-mediated antigen processing and presentation. *Nat Rev Immunol.* 2015;15(4):203–16.

37. Denzin LK, and Cresswell P. HLA-DM induces CLIP dissociation from MHC class II alpha beta dimers and facilitates peptide loading. *Cell.* 1995;82(1):155–65.

38. Takeda K, and Akira S. Toll-like receptors in innate immunity. *Int Immunol.* 2005;17(1):1–14.

39. Takeuchi O, and Akira S. Pattern recognition receptors and inflammation. *Cell.* 2010;140(6):805–20.

40. Mueller FB, Yang H, Li C, Dadhania DM, Xiang JZ, Salvatore SP, et al. RNA-sequencing of Human Kidney Allografts and Delineation of T-Cell Genes, Gene Sets, and Pathways Associated With Acute T Cell-mediated Rejection. *Transplantation.* 2024;108(4):911–22.

41. Wilhelm M, Geng A, Schaub S, and Hirsch HH. Posoleucel for BK Polyomavirus in Kidney Transplant Recipients. *J Am Soc Nephrol.* 2024;35(12):1784–5.

42. Wilhelm M, Kaur A, Geng A, Wernli M, and Hirsch HH. Donor Variability and PD-1 Expression Limit BK Polyomavirus-specific T-cell Function and Therapy. *Transplantation.* 2025;109(9):1526–39.

43. Yi JS, Cox MA, and Zajac AJ. T-cell exhaustion: characteristics, causes and conversion. *Immunology.* 2010;129(4):474–81.

44. Wherry EJ, and Kurachi M. Molecular and cellular insights into T cell exhaustion. *Nat Rev Immunol.* 2015;15(8):486–99.

45. Angeletti A, Cantarelli C, Riella LV, Fribourg M, and Cravedi P. T-cell Exhaustion in Organ Transplantation. *Transplantation.* 2022;106(3):489–99.

46. Valdor R, and Macian F. Induction and stability of the anergic phenotype in T cells. *Semin Immunol.* 2013;25(4):313–20.

47. Pfoertner S, Jeron A, Probst-Kepper M, Guzman CA, Hansen W, Westendorf AM, et al. Signatures of human regulatory T cells: an encounter with old friends and new players. *Genome Biol.* 2006;7(7):R54.

48. Aran D, Hu Z, and Butte AJ. xCell: digitally portraying the tissue cellular heterogeneity landscape. *Genome Biol.* 2017;18(1):220.

49. Savas P, Virassamy B, Ye C, Salim A, Mintoff CP, Caramia F, et al. Single-cell profiling of breast cancer T cells reveals a tissue-resident memory subset associated with improved prognosis. *Nat Med.* 2018;24(7):986–93.

50. Dalianis T, and Hirsch HH. Human polyomaviruses in disease and cancer. *Virology.* 2013;437(2):63–72.

51. Tatapudi RR, Muthukumar T, Dadhania D, Ding R, Li B, Sharma VK, et al. Noninvasive detection of renal allograft inflammation by measurements of mRNA for IP-10 and CXCR3 in urine. *Kidney Int.* 2004;65(6):2390–7.

52. Ho J, Rush DN, Karpinski M, Storsley L, Gibson IW, Bestland J, et al. Validation of urinary CXCL10 as a marker of borderline, subclinical, and clinical tubulitis. *Transplantation.* 2011;92(8):878–82.

53. Tinel C, Sauvaget V, Aouni L, Lamarthee B, Terzi F, Legendre C, et al. Transforming kidney transplant monitoring with urine CXCL9 and CXCL10: practical clinical implementation. *Sci Rep.* 2024;14(1):20357.

Supplemental Figures

Supplemental Figure 1. PCA and t-SNE Visualization of Urinary Cell Transcriptomes Across Diagnostic Categories.

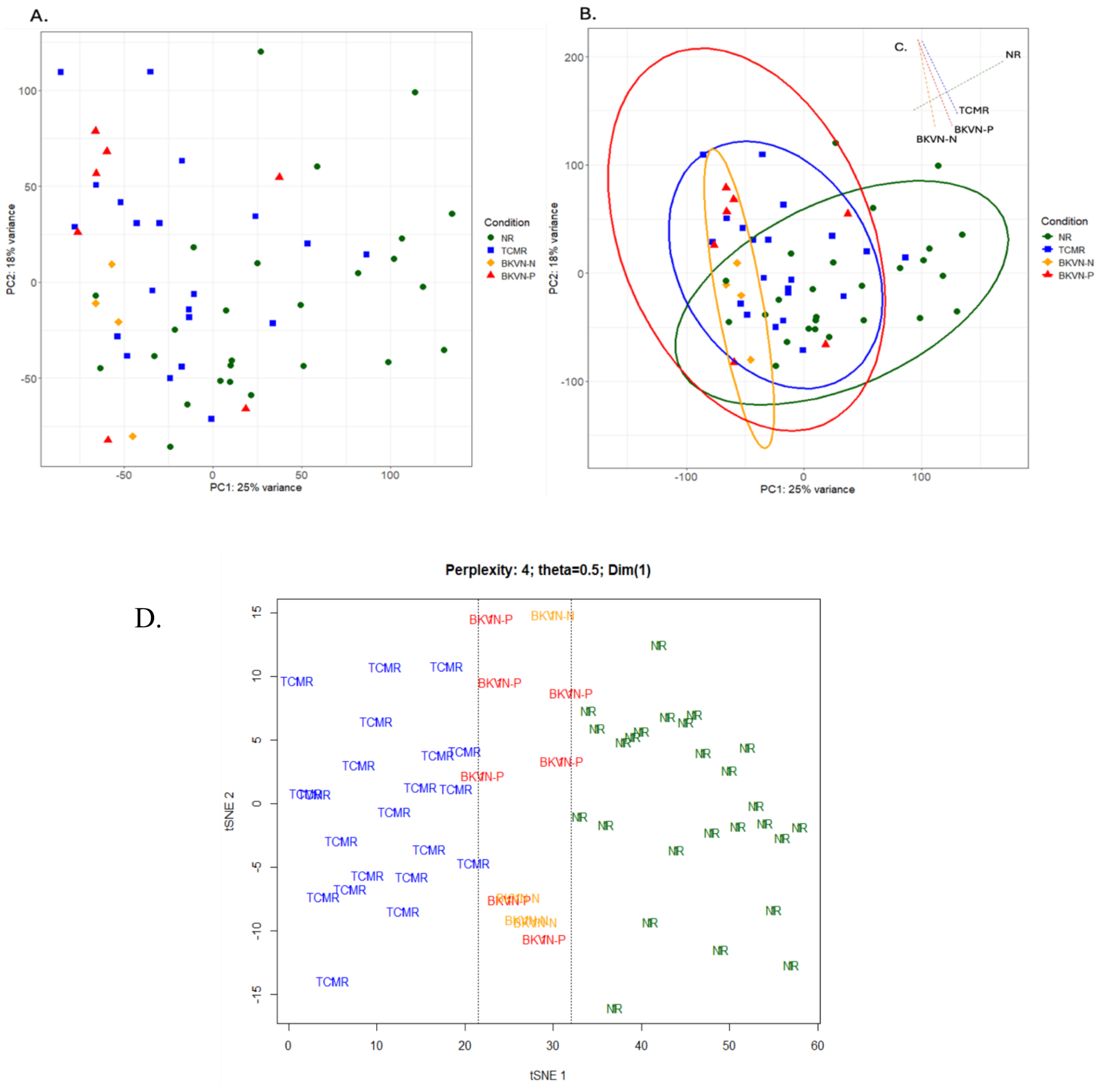

Panel A. PCA plots displaying the first two principal components (PC1 and PC2) derived from 17,444 genes, with 12 additional genes removed due to low counts following variance stabilization transformation. PC1 accounts for 25% of the total variance while PC2 explains 18%. Data points are color coded according to the four diagnostic categories, highlighting the distribution and clustering of patterns within the gene expression profiles.

Panel B. This panel presents the same PCA plots as in panel A, enhances with ellipses indicating multivariate t- distributions around the data points. The ellipses encompass a 95%

confidence region for each diagnostic category, providing a visual representation of the variability and clustering of the gene expression data. The overlap of ellipses suggests areas of shared expression patterns among conditions.

Panel C (within Panel B). The axes of the four ellipses are displayed, delineating the distinct gene expression patterns associated with each diagnostic category. The positioning of the ellipses along PC1 and PC2 indicates the relationship and differences among the diagnostic categories, offering insights into how gene expression varies across the four diagnostic categories.

Panel D. t-SNE analysis was performed using a perplexity of 4; theta 0.5, and a single output dimension to represent the 4 diagnostic categories (color coded): TCMR, acute T cell mediated rejection; BKVN-P, BK polyomavirus-associated nephropathy with intragraft inflammation; BKVN-N, BK polyomavirus- associated nephropathy without intragraft inflammation; NR, no histological features of acute or chronic rejection. High-dimensional RNA expression data were projected onto dimension 1, with each sample represented as a point along a single axis. Samples that are close together in the original high-dimension space cluster tightly along the line, preserving local structure and reflecting similarities among the conditions.

Supplemental Figure 2. MDS Plot of Urinary RNA-Seq Profiles Based on Leading Log-fold Changes.

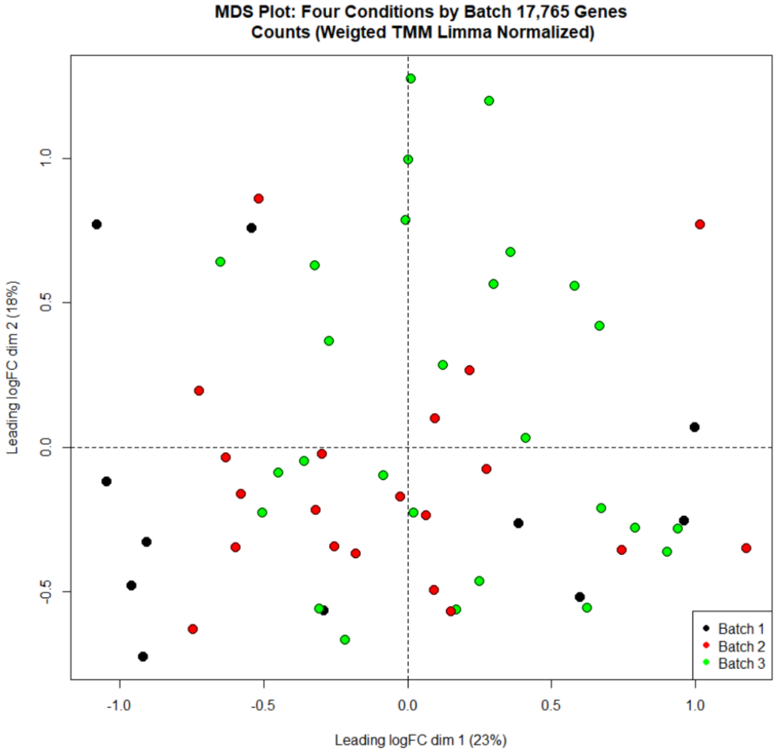

Sample batches visualized in MDS space using weighted TMM limma normalized counts. Each point in the plot represents one urine sample matched to a kidney allograft biopsy, with colors indicating batch assignment and independent of 4 diagnostic categories, TCMR, BKVN-P, BKVN-N or NR). Batch 1: Black, n=11, 3 urine samples matched to 3 NR biopsies, 1 to a BKVN-N biopsy, 3 to 3 BKVN-P biopsies, and 4 to 4 TCMR biopsies. Batch 2: Red, n=20, 12 urine samples matched to 12 NR biopsies, 1 to a BKVN-N biopsy, 2 to 2 BKVN-P biopsies, and 5 to 5 TCMR biopsies. Batch 3: Green, n=27, 11 urine samples matched to 11 NR biopsies, 12 to 12 TCMR biopsies, 2 to 2 BKVN-N biopsies and 2 urine samples matched to 1 BKVN-P biopsy. Distances between points reflect differences in global gene expression profiles, with samples closer together are more similar. The first dimension explains 23% of leading (log fold) logFC variance, and the second dimension explains 18%. Points do not cluster by batch, supporting minimal or no observable batch effect. See Supplemental Figure 3 for additional statistical confirmation of minimal or absence of a batch effect.

Supplemental Figure 3. MDS Plot of Batch Sample Distribution Using Weighted TMM-Limma Normalized Counts with K-function for Spatial Point Pattern Assessment.

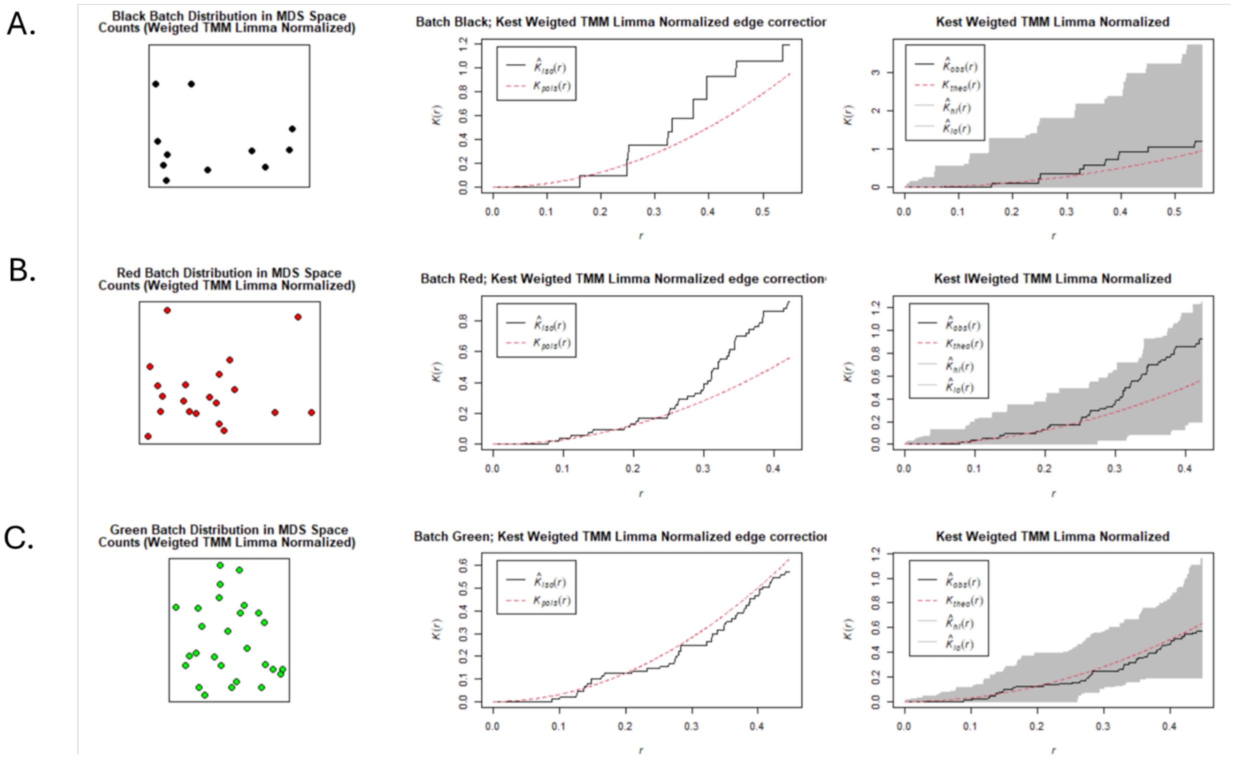

MDS distribution and K-function curves:

A: Batch 1, black, n=11, 3 urine samples matched to 3 NR biopsies, 1 to a BKVN-N biopsy, 3 to 3 BKVN-P biopsies, and 4 to 4 TCMR biopsies.

B: Batch 2, red, n=20, 12 urine samples matched to 12 NR biopsies, 1 to a BKVN-N biopsy, 2 to 2 BKVN-P biopsies, and 5 to 5 TCMR biopsies.

C: Batch 3, green, n=27, 11 urine samples matched to 11 NR biopsies, 12 to 12 TCMR biopsies, 2 to 2 BKVN-N biopsies and 2 urine samples matched to 1 BKVN-P biopsy.

Sample batches visualized in MDS space using weighted TMM limma normalized counts. Each batch displays two curves for the K-function: Kest estimates sing best edge correction; reflects the observed diversity of points at various spatial distances. Kpois[r] (red curve) reflects theoretical K-function for a perfectly random pattern, calculated under a homogenous Poisson process. The horizontal X-axis represents distance r from an arbitrary point in the pattern, and the vertical Y-axis reflects the value of the K-function at each distance. The third plot compares

observed K-function against a confidence band (shaded region) generated from simulations of Complete Spatial Randomness (CSR). If the observed values remain within the CSR confidence band, sample spatial distribution is consistent with randomness. If the observed curve is above the CSR band, point density at that distance exceeds randomness, indicating spatial clustering. If the observed curve is below the CSR band, there fewer points than expected, indicating dispersion or regularity (points repel). No significant batch effect was detected by statistical analysis, indicating similar spatial distribution patterns across batches.

Supplemental Figure 4. Agreement of Urinary RNA-Seq Counts Between Pre-and Post-biopsy Samples in a Patient with BKVN-P Biopsy: (A) Pearson Correlation; (B) Bland-Altman Plot.

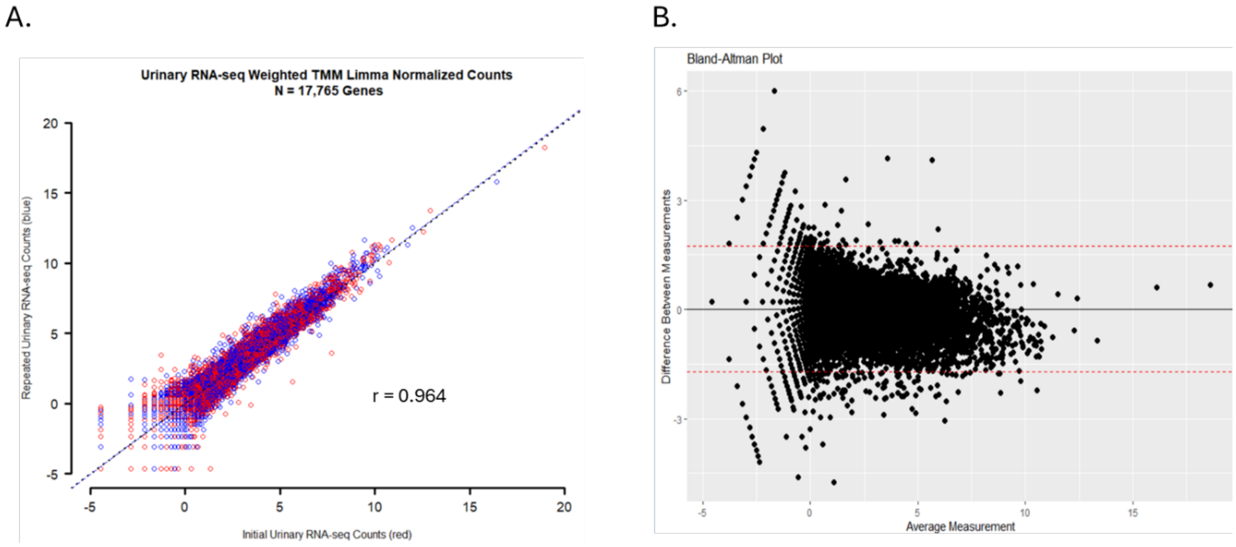

A: Pearson correlation analysis: Scatter plot depicting RNA-seq count measurements of 17,765 genes from urine matched pre-biopsy and post-biopsy urine sample collected within 6 hours of kidney biopsy. The Pearson correlation coefficient (r=0.964) indicates a high degree of concordance, surpassing the ENCODE consortium's benchmark for technical and biological replicability.

B: Bland-Altman analysis: Bland-Altman plot illustrating the agreement between pre-and post-biopsy urinary RNA-Seq measurements. The y-axis represents the difference between pre-and post-biopsy gene expression counts, while the x-axis shows the average. The horizontal lines denote the mean difference (bias) and the 95% limits of agreement, indicating the expected range within which most differences fall.
